# Zabedosertib, a novel interleukin-1 receptor-associated kinase-4 inhibitor, shows a favorable pharmacokinetic and safety profile across multiple phase 1 studies

**DOI:** 10.3389/fphar.2025.1521505

**Published:** 2025-05-30

**Authors:** Maximilian Feldmüller, Stefan J. Jodl, Bart Ploeger, Andrea Wagenfeld, Herbert Wiesinger, Frank S. Zollmann, Stefan Klein, Ruiping Zhang, Beate Rohde, Joachim Höchel

**Affiliations:** ^1^ Clinical Pharmacology, Research and Development, Pharmaceuticals, Bayer AG, Berlin, Germany; ^2^ Translational Medicine, Research and Development, Pharmaceuticals, Bayer AG, Berlin, Germany; ^3^ Model-Informed Drug Development, Research and Development, Pharmaceuticals, Bayer AG, Berlin, Germany; ^4^ Translational Science, Pharmaceuticals, Bayer AG, Berlin, Germany; ^5^ Pharma Consult, Berlin, Germany; ^6^ Research and Early Development Statistics, Research and Development, Pharmaceuticals, Bayer AG, Berlin, Germany; ^7^ Medical Affairs China, Pharmaceuticals, Bayer Healthcare Co., Ltd., Beijing, China; ^8^ Early Clinical Development, Research and Development, Pharmaceuticals, Bayer AG, Berlin, Germany

**Keywords:** safety, pharmacokinetics, pharmacodynamics IRAK4, IRAK4 inhibitor, immune-mediated inflammatory disorders, pharmacodynamics, food effect, absolute bioavailability

## Abstract

**Introduction:**

Zabedosertib, the interleukin-1 receptor-associated kinase-4 (IRAK4) inhibitor, is in clinical development as an oral therapeutic for immune-mediated inflammatory diseases and was thoroughly investigated in several phase 1 studies in healthy male volunteers.

**Methods:**

Pharmacokinetics, safety, and tolerability of zabedosertib were characterized in two clinical phase 1 studies with single oral doses up to 480 mg and multiple oral doses up to 200 mg twice daily over 10 consecutive days. The absolute oral bioavailability was determined in a third study using the intravenous microtracer methodology.

**Results:**

Zabedosertib showed good safety and tolerability without dose-limiting toxicities or severe infections. An under-proportional increase in exposure was observed with increasing dose. The observed mean accumulation ratios for the area under the concentration–time curve of 1.04–1.62 were lower than expected based on the dose-independent terminal half-life of 19–30 h. The absolute oral bioavailability was 74% at a dose of 120 mg. No food effect was observed. The pharmacokinetics could be described with a one-compartmental population-pharmacokinetic model with first-order elimination, dose-dependent bioavailability, and capacity-limited binding in plasma. The estimation of target occupancy, based on *in vitro* potency for IL-6 inhibition as a representative pro-inflammatory cytokine in a human whole-blood assay, target residence time, and unbound plasma pharmacokinetics, indicated ∼80% target occupancy over the dosing interval after the maximum feasible dose of 120 mg twice daily. This dose was the highest dose providing relevant exposure increases.

**Conclusion:**

Based on the projected target occupancy, favorable pharmacokinetics, and safety profile, as well as on distinct pharmacodynamic effects in a proof-of-mechanism study, zabedosertib 120 mg twice daily was selected for further clinical development in patient studies.

**Clinical trial registration:**

https://clinicaltrials.gov/, identifier SAD: NCT03054402, MAD: NCT03493269 (part 1), FE/abs.BA study NCT03244462 (EudraCT numbers: 2016-002668-15, 2017-001817-10, and 2016-004393-18).

## 1 Introduction

The activation of interleukin-1 receptor-associated kinase 4 (IRAK4) induces the production and activation of a variety of pro-inflammatory cytokines, such as tumor necrosis factor alpha (TNFα), interferon gamma, and the interleukins (ILs) IL-1β, IL-6, and IL-17. These cytokines play a critical role in the onset and progression of inflammatory conditions such as rheumatoid arthritis, lupus erythematodes, and hidradenitis suppurativa. The inhibition of these cytokines may therefore provide a promising approach for the treatment of such conditions (Zarrin et al., 2021), and there is, thus, growing interest in exploring the therapeutic potential of IRAK4 inhibitors, to address unmet medical needs in the treatment of immune-mediated inflammatory diseases.

One of the IRAK4 inhibitors currently in clinical development as an oral therapy for various immune-mediated diseases is zabedosertib (BAY 1834845) (Bothe et al., 2024⁠; Jodl et al., 2024). Zabedosertib shows *in vitro* IRAK4 inhibitory potential and strongly inhibits the secretion of TNFα in rat splenic cells stimulated with lipopolysaccharides (LPSs) (Bothe et al., 2024). Due to its high permeability in the Caco-2 cell assay and its low solubility in water and 0.1 M HCl (Bothe et al., 2024), it can be considered a Biopharmaceutics Classification System (BCS) class II compound (Amidon et al., 1995).

In this paper, we report on the safety and key pharmacokinetic (PK) and pharmacodynamic (PD) results of three early phase 1 studies of the novel selective IRAK4 inhibitor, zabedosertib:

• a first-in-human study of zabedosertib, a single ascending-dose (SAD) safety, tolerability, PK, and PD study (https://clinicaltrials.gov/; ID: NCT03054402),
• a multiple ascending-dose (MAD) safety, tolerability, PK, and PD study (https://clinicaltrials.gov/; ID: NCT03493269)^1^, and
• a combined food-effect and absolute bioavailability (FE/abs.BA) study (https://clinicaltrials.gov/; ID: NCT03244462)^2^.

In addition, the development of a population-pharmacokinetic model for zabedosertib and the estimation of target occupancy based on study data are described^3^.

## 2 Materials and methods

### 2.1 Overview

The three studies were conducted in compliance with the Declaration of Helsinki and ICH Good Clinical Practice and were registered with ClinicalTrials.gov and EudraCT. The study protocols were approved by the relevant independent Ethics Committees before starting the study. All study participants provided their written informed consent before entry into the study.

A brief description of the studies is given below. Their main characteristics are tabulated in Supplementary Section 1.1.

### 2.2 Study objectives

The primary objective of the SAD and MAD studies was to investigate the safety, tolerability, and PK of ascending single or multiple oral doses of zabedosertib. The primary objective of the FE/abs.BA study was to investigate the impact of concomitant food intake on the PK properties of zabedosertib, to confirm the data from the orientating food-effect arm in the SAD study and to determine the absolute bioavailability of zabedosertib using a ^13^C-microtracer approach (Lozac’h et al., 2018).

In addition, a panel of exploratory PD and safety biomarkers were investigated in the SAD and MAD studies to show first evidence of pharmacological activity. Of this panel, only the cytokine release determined *ex vivo* after stimulation is reported here.

### 2.3 Study participants

Healthy men aged 18–50 years (inclusive) were eligible for study participation in the three reported studies (details in Supplementary Sections 1.2.1 through 1.2.3).

### 2.4 Study designs and treatments

**FIGURE 1:**
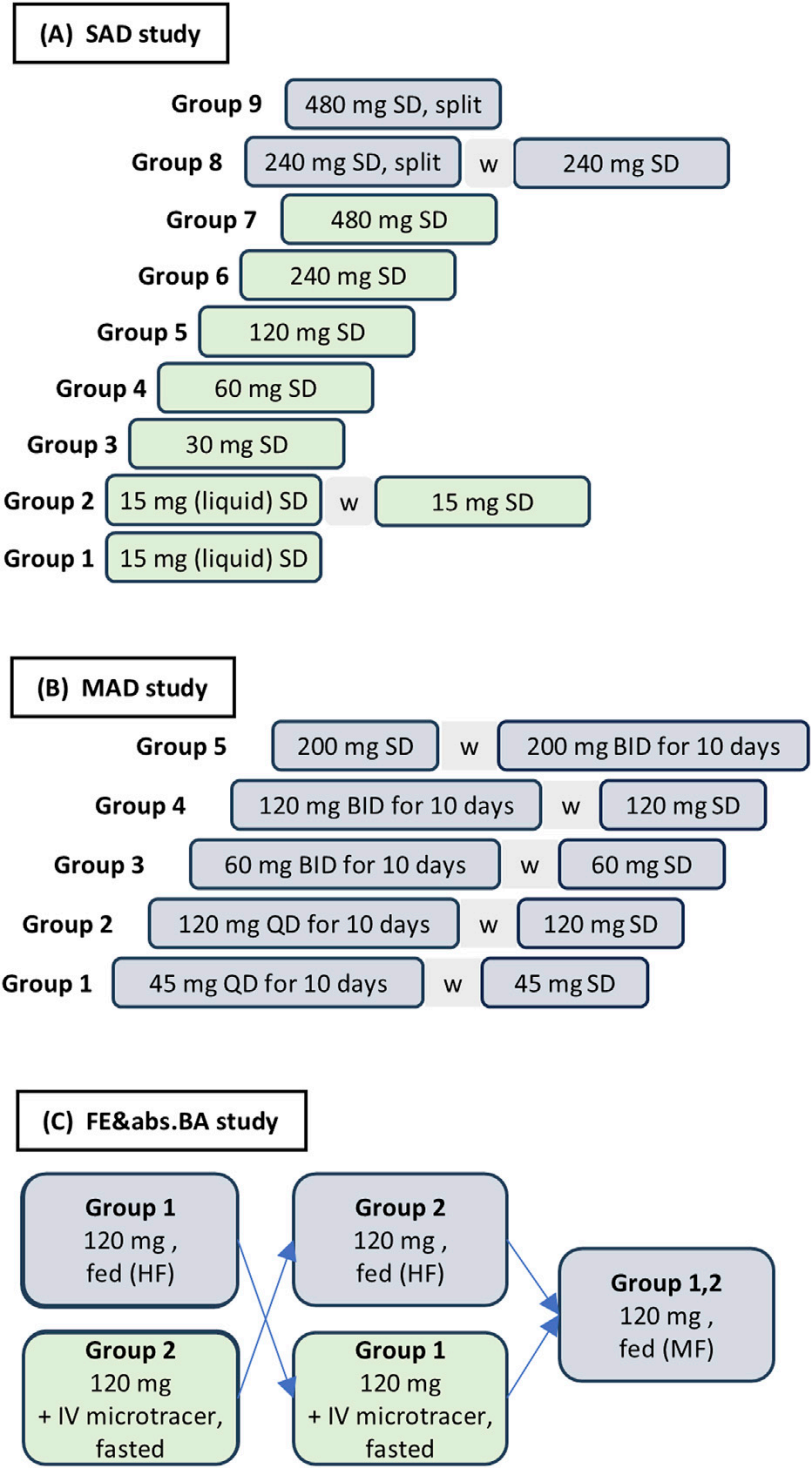
Study designs and dose groups in the three studies. Green boxes indicate treatment periods with dosing in the fasted state; blue boxes indicate treatment periods with dosing after a light meal (SAD and MAD studies) or a high-fat or moderate-fat meal (FE&abs.BA study); “w” indicates a 14-day washout period. Zabedosertib and placebo were administered as tablets unless otherwise indicated. Dose specifications refer to the dose of zabedosertib or the corresponding placebo “dose.” **(A)** The SAD study included one treatment period for participants in groups 1, 3–7, and 9 and two periods for participants in groups 2 and 8. Each group comprised eight participants; among them, six assigned to zabedosertib and two assigned to placebo. Dose splitting: dividing the dose into two halves administered 12 h apart from each other (“split”) was expected to overcome the observed bioavailability limitations at higher doses. **(B)** Each dose group comprised 10 participants, eight assigned to zabedosertib and two assigned to placebo. Group 5 started with single-dose administration, and all other groups started with multiple-dose administration. **(C)** Participants in the FE/abs.BA study were randomly allocated to intake of zabedosertib in the fasted state in period 1 and intake of zabedosertib after eating a high-fat, high-calorie meal in period 2 or vice versa. In period 3, intake of zabedosertib took place shortly after eating a moderate-fat, moderate-calorie meal. Abbreviations: BID, twice daily; FE/abs.BA, food-effect and absolute bioavailability study; HF, high-fat, high-calorie meal; IV, intravenous; MAD, multiple ascending dose; MF, moderate-fat, moderate-calorie meal; QD, once daily; SAD, single ascending dose; SD, single dose; w, 14-day washout phase.

Both the SAD and MAD studies, which also included a single-dose part, were randomized, placebo-controlled, double-blind (sponsor-unblinded), parallel-group, and ascending-dose studies (Figures 1A,B). Participants received either zabedosertib immediate-release tablets or corresponding placebo tablets (N = 52 and N = 40 received zabedosertib in the SAD and MAD studies, respectively).^4^ For the first dose steps in the SAD study, a liquid service formulation was used instead of tablets.

The FE/abs.BA study was an open-label, randomized, three-period, two-sequence cross-over study. Study participants (N = 10) were randomly allocated to one of two possible treatment sequences (Figure 1C): administration of zabedosertib in the fasted state in period 1 and following a standardized high-fat, high-calorie breakfast (FDA/CDER, 2002⁠; EMA, 2012a) in period 2 or vice versa. In period 3, zabedosertib was administered following a moderate-fat, moderate-calorie breakfast (details in Supplementary Section 1.3.1), as high-fat, high-calorie meals are not regarded as a typical breakfast worldwide. To investigate the absolute bioavailability of zabedosertib after oral administration, participants received an intravenous (IV), stable-isotope-labeled microtracer dose of zabedosertib (^13^C_6_-zabedosertib) on top of the oral zabedosertib dose taken in the fasted state.

The study designs were chosen based on regulatory guidance and the standard procedures used in similar early phase 1 studies of the study sponsor. In single- and multiple-dose first-in-men studies, ascending-dose designs are mandatory for safety reasons. The dosages to be studied were selected to cover the intended efficacious exposure range, which was derived from rodent inflammation models (LPS models), taking into account interspecies differences such as differences in protein binding. The starting dose was selected in compliance with the relevant FDA guidance (FDA/CDER, 2005). The dose range to be studied started at an exposure where the assumed maximum concentration would not reach the half-maximum inhibitory concentration (IC_50_) for IL-6 or TNFα inhibition (based on a non-clinical LPS model).

### 2.5 Procedures and assessments

The studies each comprised a screening period, up to three treatment periods (Figure 1), a follow-up period, and the end-of-study visit.

Procedures included safety assessments and blood sampling for PK analyses, zabedosertib plasma protein-binding analyses (SAD and MAD studies), and exploratory PD biomarker analyses (SAD and MAD studies).^5^ For sampling times, see Supplementary Section 1.3.2.

### 2.6 Primary variables

Primary variables for the assessment of safety and tolerability were the frequency and severity of treatment-emergent adverse events (TEAEs).

Treatment-emergent adverse events of special interest (AESIs) were (i) confirmed or suspected severe invasive bacterial infections (all studies), (ii) systemic hypersensitivity reactions (MAD study), and (iii) noninvasive infections of the skin (MAD study).

#### 2.6.1 Primary PK variables

• The area under the plasma concentration–time curve from zero to infinity after single-dose administration (AUC) (SAD and FE/abs.BA studies).
• The observed maximum drug concentration in plasma after single-dose administration (C_max_) (SAD and FE/abs.BA studies).
• The area under the plasma concentration–time curve from zero to 24 h post-dose after repeated administration (AUC(0-24)_md_) [MAD study; once daily (QD) dosing].
• The area under the plasma concentration–time curve from zero to 12 h post-dose after repeated administration (AUC(0-12)_md_) [MAD study, twice daily (BID) dosing].
• The observed maximum drug concentration in plasma after repeated administration (C_max,md_) (MAD study).
• The total body clearance after IV administration (CL) (FE/abs.BA study).
• The volume of distribution at steady state (V_ss_) (FE/abs.BA study).
• The absolute oral bioavailability (F) of zabedosertib in the fasted state (FE/abs.BA study).

### 2.7 Bioanalytical methods

Quantitative analyses of zabedosertib and [^13^C]-zabedosertib in plasma were performed using fully validated high-pressure liquid chromatography and tandem mass spectrometric detection methods; see Supplementary Section 1.4.1 for details and performance parameters.^6^ Method validation and analyses were conducted in compliance with the pertinent regulatory guidelines (FDA/CDER/CVM, 2001⁠; FDA/CDER/CVM, 2018⁠; EMA, 2011; EMA, 2012b).

The unbound fraction (f_u_) of zabedosertib in plasma was determined using equilibrium dialysis after spiking plasma samples *ex vivo* with [^14^C] zabedosertib; details are provided in Supplementary Section 1.4.2.

The impact of zabedosertib on cytokine release (e.g., TNFα) was measured after *ex vivo* stimulation of whole-blood cells with LPSs; details are provided in Supplementary Section 1.4.3.

### 2.8 Pharmacokinetic parameters

Noncompartmental PK parameters for zabedosertib and [^13^C]-zabedosertib were calculated using PK software WinNonlin v5.3 or Phoenix 8.1 (Certara Companies).

### 2.9 Statistical analyses

Statistical analyses were conducted separately for each study using the program package SAS v9.2 (SAS Institute, Cary, NC). Within each study, data from all participants receiving placebo were pooled for safety analyses. Sample sizes were planned based on safety considerations and prior experience with similar studies. All analyses were explorative.

Dose proportionality was investigated separately for the SAD and MAD studies and within the MAD study separately for single and repeated administration. Analyses of variance were performed on log-transformed, dose-normalized PK parameters, with the dose level as an independent factor. Based on these analyses, point estimates (least squares means) and exploratory 90% confidence intervals (CIs) for dose ratios were calculated by retransforming the estimates and CIs to the original scale.

In addition, power models were applied where a linear regression of dose on log-transformed AUC and C_max_ data was performed (SAD study). The point estimate and 90% CI of the slope in the respective linear regression are used to characterize the dose proportionality.

The relative bioavailability of zabedosertib administered under different conditions was investigated by performing mixed models including fixed treatment effects and random subject effects on the log-transformed AUC and C_max_ values. For each mixed model, the point estimate and 90% CI for the ratio of “condition A/condition B” (e.g., solid form vs. liquid form), derived by inverse transformation of the estimates and 90% CIs from the model, are reported.

For the evaluation of the absolute bioavailability (FE/abs.BA study), PK parameter F was calculated as F = (AUC/D)_oral_ / (AUC/D)_IV_ based on data obtained under fasted conditions. The geometric mean as point estimate and the corresponding 90% CI were calculated for this parameter, which was assumed to be log-normally distributed.

To evaluate the impact of food intake on the PK of zabedosertib (FE/abs.BA study), log-transformed AUC and C_max_ were subjected to an analysis of variance, including treatment, subject, period, and sequence effects (high-fat, high-calorie meal) or subject and period effects (moderate-fat, moderate-calorie meal). Point estimates and 90% CIs for the ratio “fed/fasted” were calculated by retransforming the analysis of variance estimates to the original scale.

### 2.10 Population-pharmacokinetic modeling methods

A population-pharmacokinetic analysis based on PK data from the SAD and MAD studies was conducted to describe the variability in the PK of zabedosertib taken as tablets under fasting conditions. The analysis was conducted using nonlinear mixed-effects modeling with NONMEM v7.3 (ICON Development Solutions, Dublin, Ireland); details are provided in Supplementary Section 1.5.

### 2.11 Estimation of target occupancy

As binding of zabedosertib to IRAK4 could not be measured *in vivo,* a model-based approach was developed to assess the potential target occupancy of zabedosertib. This approach assumed that zabedosertib target binding increases with increasing unbound zabedosertib plasma concentration and that the maximal binding capacity is considerably lower than the maximal zabedosertib concentration, resulting in maximal target occupancy toward higher zabedosertib concentrations. Furthermore, it is assumed that zabedosertib binding to the target follows a second-order kinetic process; that is, the target binding rate is dependent on the unbound zabedosertib plasma concentration and free target [see Tummino and Copeland (2008) for further details on target binding kinetics]. When all the targets are occupied, the target binding rate becomes zero, signifying that the maximal binding capacity has been attained. Zabedosertib is assumed to dissociate from IRAK4 following first-order kinetics, which can be measured *in vitro* (Tummino and Copeland, 2008). The second-order binding rate constant can be estimated from the equilibrium dissociation constant Kd. As described by Winkler et al. (2021) for the IRAK4 inhibitor zimlovisertib, Kd can be derived from the IC_50_ of the inhibition of IL6 release in an *in vitro* human whole-blood assay after stimulation with the toll-like-receptor-7 and -8 agonist resiquimod (R848); details are provided in Supplementary Section 1.6. IC_50_ was estimated by fitting the concentration–IL-6 release inhibition data to a sigmoidal E_max_ model using R package drm (Ritz et al., 2015).

The (projected) target occupancy in healthy volunteers was estimated by linking the variability in unbound PK, as described using the population PK model, to the estimated IC_50_ value from the *in vitro* human whole-blood assay and target residence time (∼5 min) using a typical model for target-binding kinetics, as described above. Information on the actual IRAK4 (target) concentration is not required under the assumption that drug concentration would be in excess of the target concentration. R package RxODE (Wang et al., 2016) was used for the estimation.

## 3 Results

### 3.1 Study participants

In total, 130 healthy men aged 18–50 years were included in the three studies. Their demographic characteristics were well balanced within each study (Table 1), with no notable differences between dose groups or treatment sequences (Supplementary Sections 2.1–2.3). All participants were evaluated for safety, PK, and PD; see Supplementary Sections 2.4–2.6 for participant disposition.

**TABLE 1:** Study participants: demographics and participant disposition.

	SAD study	MAD study	FE/abs.BA study
N evaluable	70	50	10
Sex	Male	Male	Male
Age [years]^a^	38 ± 8.1 (21–50)	35.8 ± 8.9 (20–50)	28 ± 7.9 (18–43)
BMI [kg/m^2^]^a^	25.0 ± 2.44 (19.9–30.1)	25.3 ± 2.55 (19.4–29.7)	24.4 ± 2.99 (19.8–28.7)
Ethnicity, race^b^	Mainly White participants^c^	White participants only	White participants only
*Details in*	*Supplementary Material 2.1*	*Supplementary Material 2.2*	*Supplementary Material 2.3*
Assigned to treatment	70 (zabedosertib: 52; placebo: 18)	50 (zabedosertib: 40; placebo: 10)	10 (fed (HF) > fasted >fed (MF): 5; fasted > fed (HF) >fed (MF): 5)^d^
N completed	68^e^	49^f^	10
N excluded from analysis	None	None	None
*Details in*	*Supplementary Material 2.4*	*Supplementary Material 2.5*	*Supplementary Material 2.6*

^a^ Data are mean ± standard deviation (minimum–maximum).

^b^ Self-reported ethnicity and race classification was routinely documented.

^c^ One participant classified himself as Black/African American, and another classified himself as Hawaiian/Pacific Islander.

^d^ Zabedosertib was administered after a high-fat, high-calorie meal in period 1 and in the fasted state in period 2, or vice versa, and after a moderate-fat, moderate-calorie meal in period 3.

^e^ Two participants withdrew prematurely from the study after having completed the first period (both had been assigned to a two-period schedule).

^f^ One participant discontinued after period 1 due to an adverse event present already prior to treatment.

Abbreviations: BMI, body mass index; FE/abs.BA, food-effect and absolute bioavailability; HF, high-fat, high-calorie meal; MAD, multiple ascending dose; MF, moderate-fat, moderate-calorie meal; N, number of participants; SAD, single ascending dose.

### 3.2 Safety

Zabedosertib showed good safety and tolerability. This is reflected in the fact that mainly mild TEAEs were reported and none of the TEAEs led to discontinuation of treatment. Only one adverse event, also the only serious TEAE, as described below, was assessed as severe; however, it was not related to zabedosertib due to the time elapsed since the last drug intake. Different laboratory parameters showed, in individual cases, transient changes, but there was no indication of any relationship to the dose administered and no clinically meaningful difference between zabedosertib- and placebo-treated participants. No drug-related changes were observed in vital signs or ECG parameters.

#### 3.2.1 SAD study

Zabedosertib was well tolerated across all doses. Neither severe TEAEs nor serious TEAEs or AESIs were reported. Although a numerically higher number of TEAEs were observed in the zabedosertib arm than in placebo, there was no apparent trend in the frequency of TEAEs with an increasing zabedosertib dose.

Overall, 30 of all 70 study participants (43%) experienced TEAEs [zabedosertib: 25 out of 52 (48%) and placebo: 5 out of 18 (28%)], mostly of mild intensity (details in Supplementary Sections 3.1–3.2). TEAEs of moderate intensity were nasopharyngitis and gastroenteritis in three participants (two on zabedosertib and one on placebo). The most common TEAEs (MedDRA-preferred terms) were increasing C-reactive protein, nasopharyngitis, and headache, which affected 11%, 9%, and 6% of all participants, respectively. Of these, the investigator classified only one case of moderate nasopharyngitis as related to the study drug (zabedosertib arm).

#### 3.2.2 MAD study

Fourteen of all 49 participants (29%) experienced TEAEs during the single-dose period [zabedosertib: 12 out of 39 (31%) and placebo: 2 out of 10 (20%)] (Supplementary Sections 3.3–3.4). Most of these TEAEs were of mild intensity. TEAEs of moderate intensity included nasopharyngitis, arthropod sting, joint swelling, and headache (all zabedosertib). One severe TEAE occurred 17 days after the last intake of 120 mg zabedosertib BID. This TEAE, cellulitis after a fall (investigator term: phlegmon right elbow and arm), was assessed as serious (hospitalization of the participant) and as an AESI. The event was assessed as unrelated to study drug administration due to the time elapsed since the drug intake.

During the multiple-dose period, 24 of 50 participants (48%) experienced TEAEs [zabedosertib: 21 out of 40 (52.5%) and placebo: 3 out of 10 (30%)], without clearly discernible differences between treatment groups (Supplementary Section 3.5). Most of these TEAEs were of mild intensity. TEAEs of moderate intensity included nasopharyngitis in one zabedosertib-treated participant and hemorrhoids in one placebo-treated participant. No serious TEAEs were reported. Most frequently reported TEAEs belong to the system organ classes “infections and infestations” [six participants in total (12%)] and “respiratory, thoracic, and mediastinal disorders” [five participants (10%)] (Table 2). Four participants (8%) reported TEAEs that the investigator considered related to the study drug. These TEAEs included oral herpes, nasopharyngitis, eructation with flatulence, and motion sickness in zabedosertib-treated participants and breath odor in a placebo-treated participant. Two cases of oral herpes were assessed and reported as AESIs: one case (no details provided), assessed as mild and not related, occurred 23 days after the last dose of 45 mg zabedosertib QD (+midazolam), and the other case (two small lesions on the upper lip), assessed as mild and related to study drug, occurred 2 days after the last dose of 120 mg zabedosertib QD (+midazolam).

**TABLE 2:** Treatment-emergent adverse events following administration of zabedosertib or placebo–overall and related to treatment (MAD study, multiple-dose period).

MedDRA	Placebo	Zabedosertib					Total
Primary system organ class Preferred term	(N = 10)	45 mg QD	120 mg QD	60 mg BID	120 mg BID	200 mg BID	N = 50 (100%)
		N = 8 (100%)	N = 8 (100%)	N = 8 (100%)	N = 8 (100%)	N = 8 (100%)	
*All events irrespective of the investigator’s assessment of the drug-event relationship*							
*Any TEAE*, n (%)	*3 (30.0)*	*3 (37.5)*	*4 (50.0)*	*6 (75.0)*	*4 (50.0)*	*4 (50.0)*	*24 (48.0)*
Cardiac disorders	0	1 (12.5)	0	0	0	0	1 (2.0)
Palpitations	0	1 (12.5)	0	0	0	0	1 (2.0)
Ear and labyrinth disorders	0	0	0	0	1 (12.5)	1 (12.5)	2 (4.0)
Motion sickness	0	0	0	0	0	1 (12.5)	1 (2.0)
Tinnitus	0	0	0	0	1 (12.5)	0	1 (2.0)
Eye disorders	0	0	0	0	0	1 (12.5)	1 (2.0)
Ocular discomfort	0	0	0	0	0	1 (12.5)	1 (2.0)
Gastrointestinal disorders	2 (20.0)	0	0	0	0	1 (12.5)	3 (6.0)
Breath odor	1 (10.0)	0	0	0	0	0	1 (2.0)
Eructation	0	0	0	0	0	1 (12.5)	1 (2.0)
Flatulence	0	0	0	0	0	1 (12.5)	1 (2.0)
Hemorrhoids	1 (10.0)	0	0	0	0	0	1 (2.0)
Lip blister	1 (10.0)	0	0	0	0	0	1 (2.0)
General disorders and administration site conditions	0	0	1 (12.5)	0	0	0	1 (2.0)
Fatigue	0	0	1 (12.5)	0	0	0	1 (2.0)
Infections and infestations	1 (10.0)	1 (12.5)	1 (12.5)	1 (12.5)	2 (25.0)	0	6 (12.0)
Nasopharyngitis	1 (10.0)	0	0	1 (12.5)	2 (25.0)	0	4 (8.0)
Oral herpes	0	1 (12.5)	1 (12.5)	0	0	0	2 (4.0)
Injury, poisoning, and procedural complications	1 (10.0)	1 (12.5)	1 (12.5)	0	0	0	3 (6.0)
Contusion	0	1 (12.5)	0	0	0	0	1 (2.0)
Fall	0	1 (12.5)	0	0	0	0	1 (2.0)
Limb injury	1 (10.0)	0	0	0	0	0	1 (2.0)
Musculoskeletal and connective tissue disorders	0	1 (12.5)	1 (12.5)	1 (12.5)	1 (12.5)	0	4 (8.0)
Limb discomfort	0	0	1 (12.5)	0	0	0	1 (2.0)
Muscle tightness	0	0	0	1 (12.5)	0	0	1 (2.0)
Musculoskeletal pain	0	0	0	0	1 (12.5)	0	1 (2.0)
Myalgia	0	1 (12.5)	0	0	0	0	1 (2.0)
Nervous system disorders	0	1 (12.5)	1 (12.5)	0	1 (12.5)	1 (12.5)	4 (8.0)
Dizziness	0	1 (12.5)	0	0	0	1 (12.5)	2 (4.0)
Headache	0	0	1 (12.5)	0	1 (12.5)	0	2 (4.0)
Psychiatric disorders	0	0	0	2 (25.0)	0	0	2 (4.0)
Initial insomnia	0	0	0	1 (12.5)	0	0	1 (2.0)
Insomnia	0	0	0	1 (12.5)	0	0	1 (2.0)
Respiratory, thoracic, and mediastinal disorders	0	0	1 (12.5)	2 (25.0)	1 (12.5)	1 (12.5)	5 (10.0)
Cough	0	0	0	0	1 (12.5)	0	1 (2.0)
Epistaxis	0	0	0	1 (12.5)	0	0	1 (2.0)
Oropharyngeal pain	0	0	1 (12.5)	0	0	1 (12.5)	2 (4.0)
Rhinorrhea	0	0	0	1 (12.5)	0	0	1 (2.0)
Skin and subcutaneous tissue disorders	0	0	2 (25.0)	1 (12.5)	0	1 (12.5)	4 (8.0)
Acne	0	0	1 (12.5)	0	0	0	1 (2.0)
Dermatitis contact	0	0	0	1 (12.5)	0	0	1 (2.0)
Night sweats	0	0	0	0	0	1 (12.5)	1 (2.0)
Rash macular	0	0	1 (12.5)	0	0	0	1 (2.0)
*Events that the investigator assessed as related to study drug*							
*Any study-drug-related TEAE*, n (%)	*1 (10.0)*	*0*	*1 (12.5)*	*0*	*1 (12.5)*	*1 (12.5)*	*4 (8.0)*
Ear and labyrinth disorders	0	0	0	0	0	1 (12.5)	1 (2.0)
Motion sickness	0	0	0	0	0	1 (12.5)	1 (2.0)
Gastrointestinal disorders	1 (10.0)	0	0	0	0	1 (12.5)	2 (4.0)
Breath odor	1 (10.0)	0	0	0	0	0	1 (2.0)
Eructation	0	0	0	0	0	1 (12.5)	1 (2.0)
Flatulence	0	0	0	0	0	1 (12.5)	1 (2.0)
Infections and infestations	0	0	1 (12.5)	0	1 (12.5)	0	2 (4.0)
Nasopharyngitis	0	0	0	0	1 (12.5)	0	1 (2.0)
Oral herpes	0	0	1 (12.5)	0	0	0	1 (2.0)

TEAEs were coded using the Medical Dictionary for Regulatory Activities (MedDRA) version 23.0. All TEAEs were reported as recovered/resolved or recovering/resolving at the end of the study. The investigator attributed none of the TEAEs to the subtherapeutic dose of midazolam given on the last day of the multiple-dose period.

Abbreviations: BID, twice daily; N, number of participants; QD, once daily; TEAE, treatment-emergent adverse event.

#### 3.2.3 Food-effect/absolute bioavailability study

In the FE/abs.BA study, 8 of 10 study participants (80%) experienced at least one TEAE, without discernible differences between treatment conditions. All TEAEs were of mild intensity, and none of them was serious or an AESI (Supplementary Section 3.6). Most common were various gastrointestinal disorders [six participants in total (60%)], nasopharyngitis [five participants (50%)], and headache [four participants (40%)] (Supplementary Section 3.7). TEAEs assessed as study drug related were reported for only one participant, which included blurred vision, headache, paresthesia, and dry skin after administration of 120 mg zabedosertib following a high-fat meal.

### 3.3 Pharmacokinetics

#### 3.3.1 Single-dose pharmacokinetics

The evaluation of the single-dose PK of zabedosertib focuses on the data obtained after administration of zabedosertib tablets in the fasted state (Table 3). (For a complete overview of the PK data obtained in the SAD study, see Supplementary Section 4.1.)

**TABLE 3:** Pharmacokinetic parameters of zabedosertib (total) in plasma after single-dose administration of zabedosertib tablets in the fasted state.

Parameter	Unit	SAD study						FE/abs.BA study
		15 mg	30 mg	60 mg	120 mg	240 mg	480 mg	120 mg
		N = 5	N = 5	N = 6	N = 6	N = 5	N = 6	N = 10
AUC	mg·h/L	32.1 (30.6)	69.5 (28.5)	107 (60.0)	119 (41.7)	182 (50.3)	188 (34.1)	161 (28.1)
AUC(0-24)	mg·h/L	16.5 (24.0)	33.0 (12.8)	49.6 (35.2)	57.3 (22.8)	75.5 (25.7)	78.9 (18.5)	N/D
C_max_	mg/L	1.13 (25.8)	1.92 (9.64)	2.91 (31.6)	3.30 (21.9)	4.43 (16.6)	4.65 (18.3)	3.22 (19.3)
t_max_ ^a^	h	2.00 [1.0–5.1]	3.50 [2.5–3.5]	3.00 [2.5–6.1]	3.25 [2.5–4.0]	2.50 [1.0–4.0]	1.50 [1.0–5.0]	5.00 [1.5–6.0]
t_1/2_	h	24.0 (16.5)	26.5 (24.9)	26.7 (35.3)	24.4 (29.0)	24.3 (23.0)	22.8 (27.0)	26.1 (23.6)
CL/F	L/h	0.467 (30.6)	0.432 (28.5)	0.561 (60.0)	1.01 (41.7)	1.32 (50.3)	2.56 (34.1)	0.745 (28.1)
MRT	h	34.1 (15.1)	37.8 (19.0)	37.4 (33.8)	34.7 (28.7)	38.7 (27.0)	39.1 (24.2)	43.2 (16.3)
Vz/F	L	16.2 (15.9)	16.5 (13.1)	21.6 (31.0)	35.6 (17.7)	46.2 (26.8)	84.2 (12.9)	28.1 (9.74)

Data are geometric mean followed by coefficient of variation [%] in parentheses unless indicated otherwise.

^a^ Median [range].

Abbreviations: AUC, area under the concentration–time curve from time zero to infinity after single-dose administration; AUC(0-24), AUC from time zero to 24 h post-dose; CL/F, total body clearance of drug calculated after extravascular administration (apparent oral clearance); C_max_, maximum observed drug concentration after single-dose administration; MRT, mean residence time for extravascular administration; N, number of participants; N/D, not determined; SAD, single ascending dose; t_1/2_, half-life associated with the terminal slope; t_max_, time to maximum concentration; Vz/F, apparent volume of distribution during terminal phase after extravascular administration.

**FIGURE 2:**
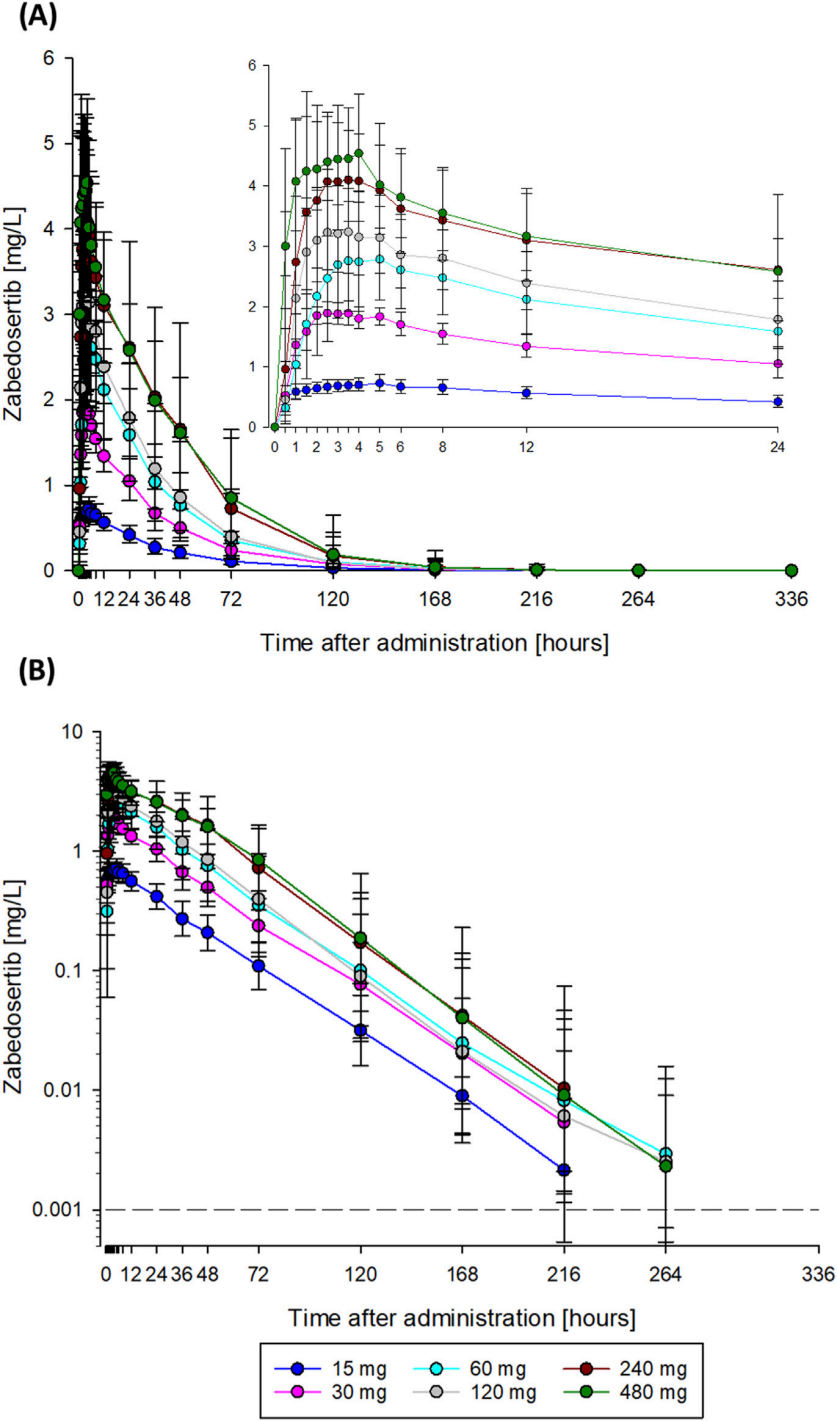
Mean zabedosertib plasma concentration–time curves obtained after single administration of zabedosertib (15–480 mg) taken as tablets in the fasted state (SAD study). Data are represented as geometric means and standard deviations. The dashed line indicates the lower limit of quantification. **(A)** Linear scale; **(B)** semi-logarithmic scale. N = 5–6 participants per dose group. Abbreviations: SAD, single ascending dose.

**FIGURE 3:**
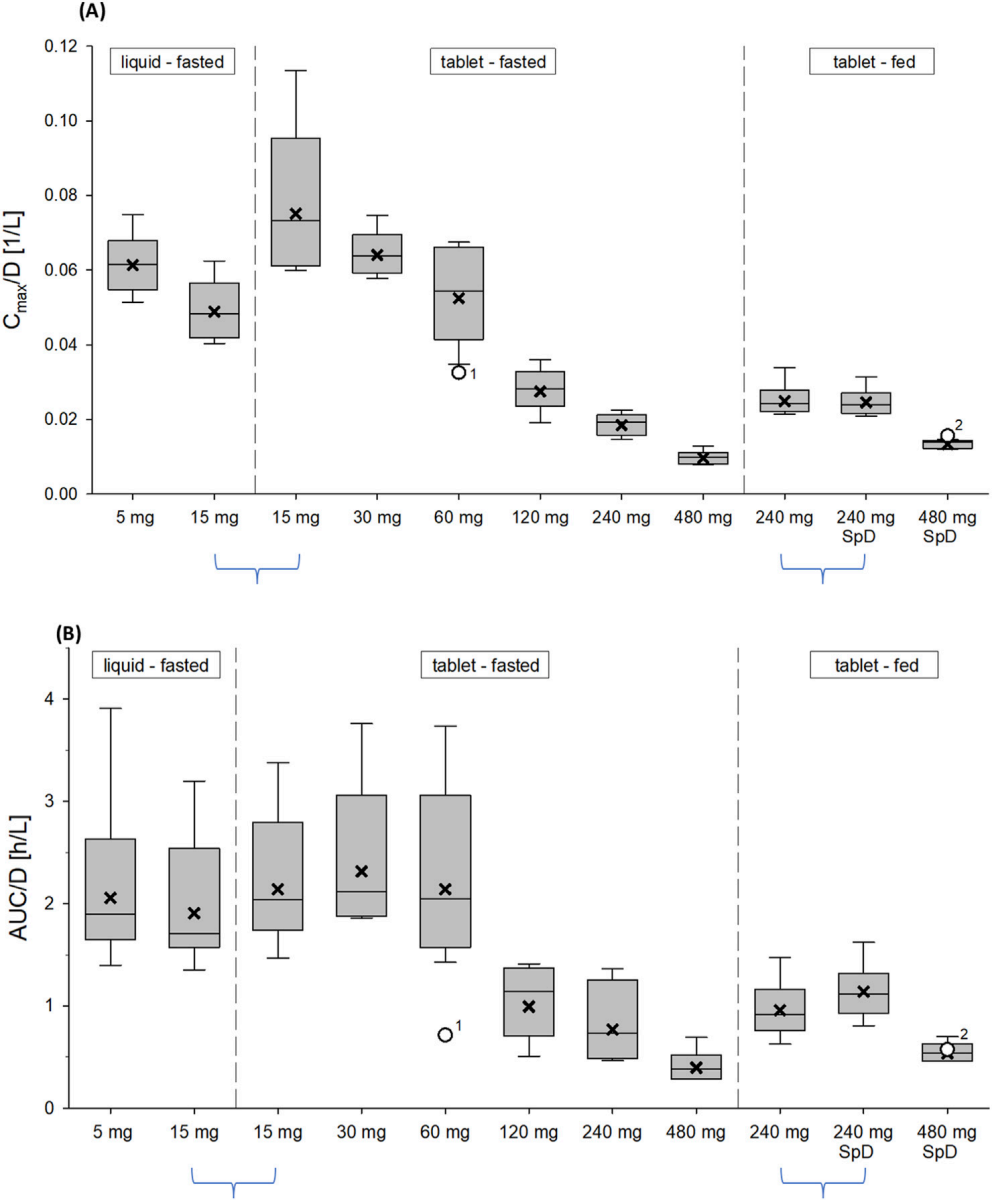
Assessment of dose proportionality: dose-normalized C_max_ **(A)** and AUC **(B)** of zabedosertib after single oral administration of zabedosertib (5–480 mg) (SAD study). Box: 25th to 75th percentile; horizontal line: median; cross: geometric mean; whiskers: 10th to 90th percentile. Box plots represent Caucasian participants; other ethnicities are indicated as individual data points (1: Black or African American; 2: Native Hawaiian or other Pacific Islander). In total, nine dose groups of 5–6 participants each were studied. Each participant received either one single dose or a split dose of zabedosertib, except for participants in two groups (indicated by blue horizontal braces), who received two doses separately by a 14-day washout. Abbreviations: AUC/D, area under the concentration–time curve divided by dose; C_max_, maximum observed drug concentration divided by dose; SAD, single ascending dose; SpD, split dose.

After single oral administration of 15–480 mg zabedosertib in the SAD study, zabedosertib plasma concentrations increased until 2–3 h post-dose, remained on a plateau until approximately 6 h post-dose, and subsequently decreased slowly (Figure 2). AUC and C_max_ values increased with dose, but sub-proportionally at doses above 60 mg or 30 mg, respectively, as illustrated in Figure 3. Statistical analyses using analysis of variance and power models also indicated that the zabedosertib exposure did not increase proportionally after single-dose administration in the fasted state (Supplementary Section 4.2). The interindividual variability (geometric CV) was generally low for C_max_ and moderate for AUC.

The mean times to C_max_ (1.5–3.5 h) and the terminal half-lives (23–27 h) determined were similar in all dose groups, with no obvious dose dependency. The apparent oral clearance of zabedosertib was similar in the lower-dose groups (15 mg–60 mg) but increased with dose in the higher-dose groups. In line with this, the apparent volume of distribution also increased with dose for doses >60 mg. That the apparent oral clearance and volume of distribution changed at the same rate with dose while the terminal half-life remained constant suggests a decreasing oral bioavailability with increasing dose. No further increase in exposure was observed for doses >120 mg.

The f_u_ of zabedosertib seemed to increase with zabedosertib dose and plasma concentration (Supplementary Sections 4.3, 4.4). The highest geometric mean f_u_ values were observed 4–5 h post-dose in the 240 mg and 480 mg split-dose groups (7.69% and 8.68%, respectively) (for comparison, the geometric mean f_u_ in pre-dose samples and placebo samples spiked with 0.1 mg/L [^14^C] zabedosertib ranged between 1.44% and 4.00%). In most treatment groups, f_u_ had decreased to some extent by 24 h post-dose and pretreatment values were reached at 96 h post-dose.

The single-dose PK parameters obtained in the FE/abs.BA study under fasting conditions (Table 3, right-hand side) were largely similar to the parameters determined in the SAD study.

The above-described lack of dose proportionality was also observed in the MAD study after single-dose administration in the fed state. Geometric mean dose-normalized zabedosertib AUC and C_max_ values (unbound) visibly decreased with increasing dose, both after QD and BID administration (Supplementary Section 4.5).

#### 3.3.2 Multiple-dose pharmacokinetics

The time course of the zabedosertib concentrations determined daily during the 10-day multiple-dose treatment period, before and 4 h after each morning dose, indicates that the PK steady state was reached after 2–3 days of treatment in all dose groups (Supplementary Section 4.6). After discontinuation of treatment, zabedosertib plasma concentrations decreased largely in parallel in all dose groups. There was no major difference in the elimination phases of the PK curves between single- and multiple-dose administrations. That the concentration of zabedosertib was below the lower limit of quantification in all pre-dose samples collected at the beginning of the single-dose period following multiple-dose administration (MAD study, dose groups 1–4) confirms that the washout of 14 days was sufficient.

**FIGURE 4:**
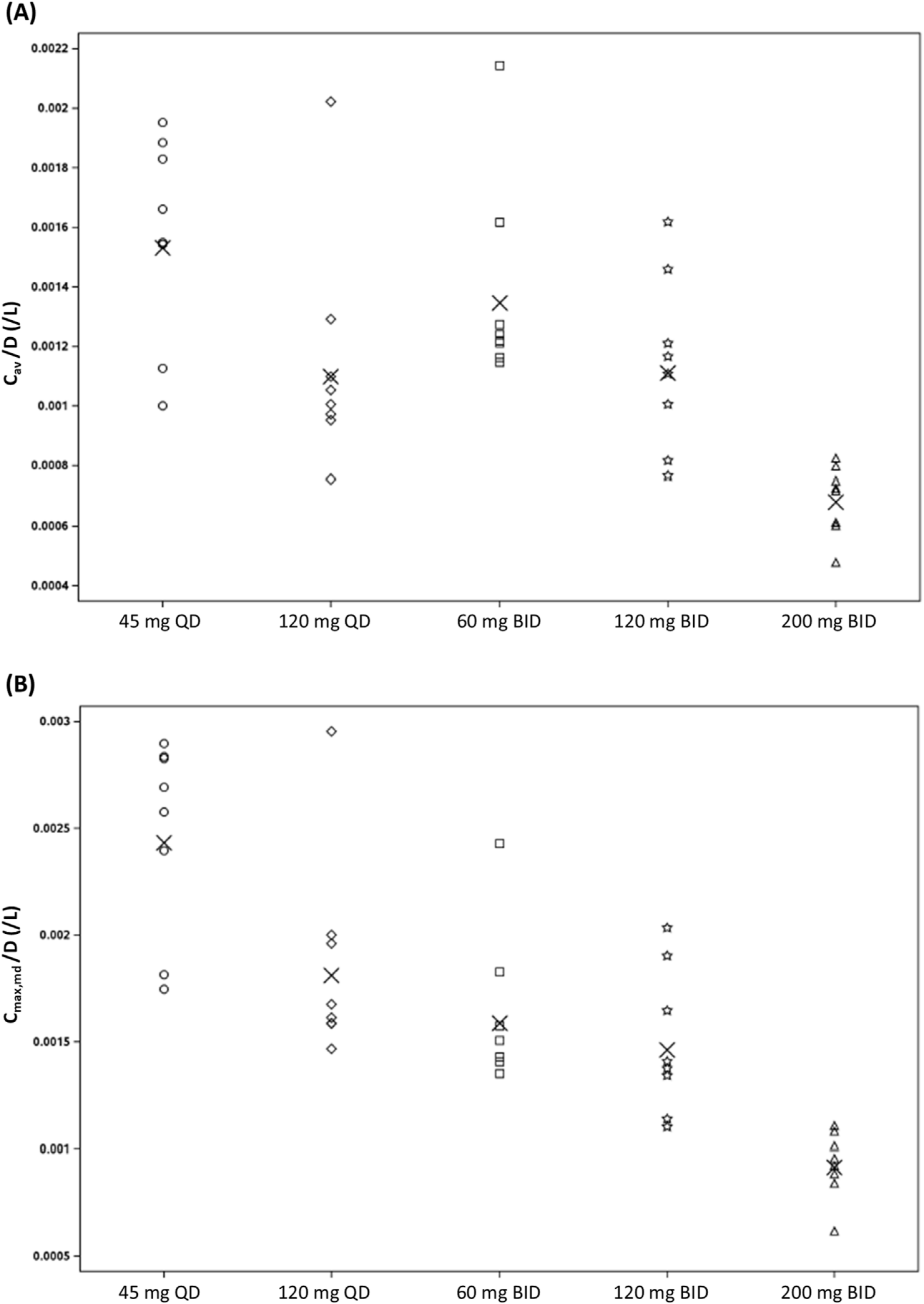
Assessment of dose proportionality: scatterplot of **(A)** dose-normalized C_av,md_ and **(B)** dose-normalized C_max,md_ of zabedosertib (unbound) after repeated oral administration of zabedosertib (MAD study; individual data). Crosses indicate the geometric mean. Abbreviations: BID, twice daily; C_av_/D, average concentration within a dosing interval divided by dose; C_max,md_/D, maximum observed drug concentration within a dosing interval divided by dose; MAD, multiple ascending dose; QD, once daily.

An overview of the multiple-dose PK parameters of (estimated) unbound zabedosertib is given in Table 4. (Corresponding parameters for total zabedosertib are summarized in Supplementary Section 4.7.) As already observed after single-dose administration, the exposure of zabedosertib increased with dose, but sub-proportionally at doses >60 mg. Accordingly, the geometric mean dose-normalized average concentration (C_av_) and C_max_ of zabedosertib within a dosing interval decreased with increasing dose, both after QD and BID administrations, as illustrated in Figure 4. The point estimates and 90% CIs for C_av_ and C_max_ ratios (higher dose/lower dose) showed a similar pattern (Table 5).

**TABLE 4:** Pharmacokinetic parameters of zabedosertib (unbound) after 10-day administration of zabedosertib tablets in the fed state (MAD study).

Parameter	Unit	45 mg QD	120 mg QD	60 mg BID	120 mg BID	200 mg BID
AUC(0-12)_md_	mg·h/L	1.01 (21.6)	2.00 (25.4)	1.94 (21.9)	3.20 (26.3)	3.26 (18.3)
AUC(0-24)_md_	mg·h/L	1.65 (24.7)	3.17 (29.4)	3.88 (21.9)^b^	6.40 (26.3)^b^	6.53 (18.3)^b^
AUC(0-24)_md_/D	h/L	0.0367 (24.7)	0.0264 (29.4)	0.0323 (21.9)^b^	0.267 (26.3)^b^	0.0163 (18.3)^b^
C_max,md_	mg/L	0.109 (20.4)	0.217 (22.8)	0.190 (19.8)	0.351 (22.5)	0.366 (18.6)
t_max,md_ ^a^	h	3.5 [1.5–5.0]	4.0 [2.0–5.0]	4.0 [1.5–5.0]	3.9 [1.0–5.8]	4.5 [0.0–5.0]
t_1/2,md_	h	21.2 (28.5)	22.6 (24.5)	28.9 (21.4)	25.8 (18.8)	27.6 (17.3)
C_av_	mg/L	0.0689 (24.7)	0.132 (29.4)	0.161 (21.9)	0.267 (26.3)	0.272 (18.3)
CL_md_/F	L/h	27.2 (24.7)	37.9 (29.4)	31.0 (21.9)	37.5 (26.3)	61.3 (18.3)
V_z,md_/F	L	832 (12.5)	1230 (14.8)	1290 (11.3)	1390 (20.2)	2440 (14.2)
PTF	%	96.7 (14.7)	103 (23.3)	31.0 (24.5)	56.9 (23.5)	58.5 (33.0)
R_A_AUC		1.26 (18.8)	1.14 (13.7)	1.72 (28.9)	1.61 (23.0)	1.56 (17.0)
R_A_C_max_		1.22 (19.0)	1.18 (18.3)	1.53 (32.4)	1.62 (29.3)	1.46 (21.9)
R_LIN_		0.757 (15.7)	0.628 (20.0)	0.455 (9.60)	0.521 (23.0)	0.484 (17.4)

Data are geometric mean followed by coefficient of variation [%] in parentheses unless indicated otherwise.

^a^ Median [minimum–maximum].

^b^ 2 × AUC(0-12).

Abbreviations: AUC(0-x)_md_, area under the concentration–time curve from time zero to x hours post-dose after repeated administration; AUC(0-x)_md_/D, AUC(0-x)_md_ divided by dose; BID, twice daily; C_av_, average concentration within a dosing interval after multiple dosing; CL_md_/F, total body clearance of drug calculated after extravascular application; C_max,md_, maximum drug concentration in plasma after repeated administration; D, daily dose (e.g., 120 mg for 60 mg BID); md, multiple-dose administration; PTF, peak-trough fluctuation; QD, once daily; R_A_AUC, accumulation ratio calculated as AUCτ at steady state/AUCτ after single dose, τ representing the dosing interval; R_A_C_max_, accumulation ratio for C_max_; R_LIN_, linearity factor calculated as AUCτ at steady state/AUC after single dose; t_1/2,md_, half-life associated with terminal slope; t_max,md_, time to reach maximum drug concentration; V_z,md_/F, apparent volume of distribution during terminal phase after extravascular administration.

**TABLE 5:** Assessment of dose proportionality of zabedosertib (unbound) in plasma after repeated oral administration of zabedosertib (MAD study).

Parameter (unit)	Ratio	N	LS-mean	90% confidence interval	Geom. CV%
C_av_/D	120 mg QD/45 mg QD	8/8	0.718	(0.568; 0.909)	27.2
(h/L)	120 mg QD/60 mg BID	8/8	0.817	(0.653; 1.02)	25.9
	120 mg BID/60 mg BID	8/8	0.826	(0.670; 1.02)	24.2
	200 mg BID/120 mg BID	8/8	0.612	(0.503; 0.745)	22.6
C_max,md_/D	120 mg QD/45 mg QD	8/8	0.745	(0.617; 0.899)	21.6
(1/L)	120 mg QD/60 mg BID	8/8	0.876	(0.728; 1.06)	21.4
	120 mg BID/60 mg BID	8/8	0.921	(0.765; 1.11)	21.2
	200 mg BID/120 mg BID	8/8	0.627	(0.524; 0.751)	20.7

Abbreviations: C_av_/D, average drug concentration within a dosing interval after multiple administration divided by dose; C_max,md_/D, maximum observed drug concentration within a dosing interval after multiple administration divided by dose; CV%, coefficient of variation; LS, least squares.

The geometric mean accumulation ratios for the unbound AUC (RA_AUC_, calculated as AUCτ at steady state/AUCτ after single dose; τ representing the dosing interval) also decreased with increasing dose. They ranged from 1.14 to 1.72, which is less than the theoretical value estimated from the terminal half-life and the length of the dosing interval. The geometric mean linearity factor of unbound zabedosertib (R_LIN_, calculated as AUCτ at steady state/AUC after single dose) was <1 in all dose groups (range: 0.455–0.757).

#### 3.3.3 Absolute and relative bioavailability

The PK parameters of zabedosertib in plasma determined after IV administration of a microdose of [^13^C_6_]-labeled zabedosertib in the FE/abs.BA study are summarized in Table 6, together with the parameters determined in the same participants after oral administration of 120 mg unlabeled zabedosertib. (For mean [^13^C_6_]-zabedosertib plasma concentration–time curves, see Supplementary Section 4.8.)

**TABLE 6:** PK parameters of zabedosertib in plasma after single oral administration of 120 mg zabedosertib in fasted and fed states with evaluation of food effects, and PK parameters of zabedosertib after IV administration of a microtracer (FE/abs.BA study).

Parameter	Unit	Zabedosertib 120 mg taken after a high-fat, high-calorie meal	Zabedosertib 120 mg taken on an empty stomach	Zabedosertib 120 mg taken after a moderate-fat, moderate-calorie meal	[^13^C_6_]-zabedosertib 0.100 mg given intravenously
		N = 10	N = 10	N = 10	N = 10
C_max_	mg/L	4.63 (22.4)	3.22 (19.3)	4.59 (15.9)	10.7 (23.6)
t_max_ ^a^	h	5.00 [1.50–5.02]	5.00 [1.50–6.02]	4.00 [2.00–5.00]	0.233 [0.233–0.250]
AUC	mg·h/L	158 (35.4)	161 (28.1)	172 (37.4)	181 (31.2)
t_1/2_	h	25.7 (26.4)	26.1 (23.6)	27.0 (25.6)	25.3 (24.1)
MRT	h	37.0 (23.1)	43.2 (16.3)	39.2 (25.3)	N/A
MRT_iv_		N/A	N/A	N/A	32.2 (23.2)
CL/F	L/h	0.760 (35.4)	0.745 (28.1)	0.699 (37.4)	N/A
CL		N/A	N/A	N/A	0.551 (31.2)
Vz/F	L/h	28.1 (12.0)	28.1 (9.74)	27.3 (12.4)	N/A
Vz		N/A	N/A	N/A	20.1 (11.8)
Vss	--	N/A	N/A	N/A	17.7 (13.8)
Evaluation of food effects					
Cmax^b^	HF/fasted	1.44 (1.29–1.61) 13.7%			N/A
AUC^b^		0.980 (0.916–1.049) 8.14%			N/A
Cmax^b^	MF/fasted		1.43 (1.29–1.58) 12.3%		N/A
AUC^b^			1.07 (0.967–1.18) 12.0%		N/A

Data are geometric mean followed by coefficient of variation [%] in parentheses unless indicated otherwise.

^a^ Median [minimum–maximum].

^b^ Geometric least squares mean followed by 90% confidence interval in parentheses and coefficient of variation.

Abbreviations: AUC, area under the concentration–time curve from time zero to infinity after single-dose administration; CL, total body clearance of drug in the measured matrix calculated after intravenous administration; CL/F, total body clearance of drug calculated after extravascular administration (apparent oral clearance); C_max_, maximum observed drug concentration after single-dose administration; HF, high-fat, high-calorie meal; MF, moderate-fat, moderate-calorie meal; MRT, mean residence time for extravascular administration; MRT_IV_, mean residence time after intravenous administration; N, number of participants; N/A, not applicable; t_1/2_, half-life associated with the terminal slope; t_max_, time to maximum concentration; V_z_, apparent volume of distribution during terminal phase after intravascular administration; V_z_/F, apparent volume of distribution during terminal phase after extravascular administration.

The clearance of zabedosertib determined after IV administration was relatively low (0.551 L/h); the volume of distribution at the steady state was moderate (17.7 L), and the geometric mean half-life was long with 25.3 h and similar to the half-life observed in the same participants after oral intake of 120 mg zabedosertib in the fasted state (26.1 h). The absolute oral bioavailability of zabedosertib (120 mg orally in the fasted state) was calculated to be 73.9% (90% CI: 68.5%–80.8%; geom. CV%: 14.6).

The comparison of the PK parameters determined in six participants in the SAD study who received 15 mg zabedosertib in solid and liquid forms (Supplementary Section 4.1) showed that the AUC and C_max_ of zabedosertib in plasma were higher after tablet intake than after intake of the oral solution by approximately 25% and 60%, respectively (Supplementary Section 4.9; Supplementary Table 1). The rate of absorption immediately after dosing, in contrast, was faster after intake of the oral solution.

The comparison of the PK parameters determined in five participants in the SAD study who received 240 mg zabedosertib in the fed state as one dose and in two halves administered 12 h apart (Supplementary Section 4.1) showed that the AUC was only marginally affected by dose splitting (Supplementary Section 4.9; Supplementary Table 2); that is, dose splitting did not overcome the bioavailability limitations observed at higher doses.

#### 3.3.4 Impact of concomitant food intake

The evaluation of the impact of concomitant food intake on the exposure of zabedosertib focuses on the results of the dedicated food-effect study (FE/abs.BA study). The PK data obtained in the SAD study after administration of 240 mg and 480 mg zabedosertib in fed and fasted states are provided in Supplementary Section 4.1 for comparison.

**FIGURE 5:**
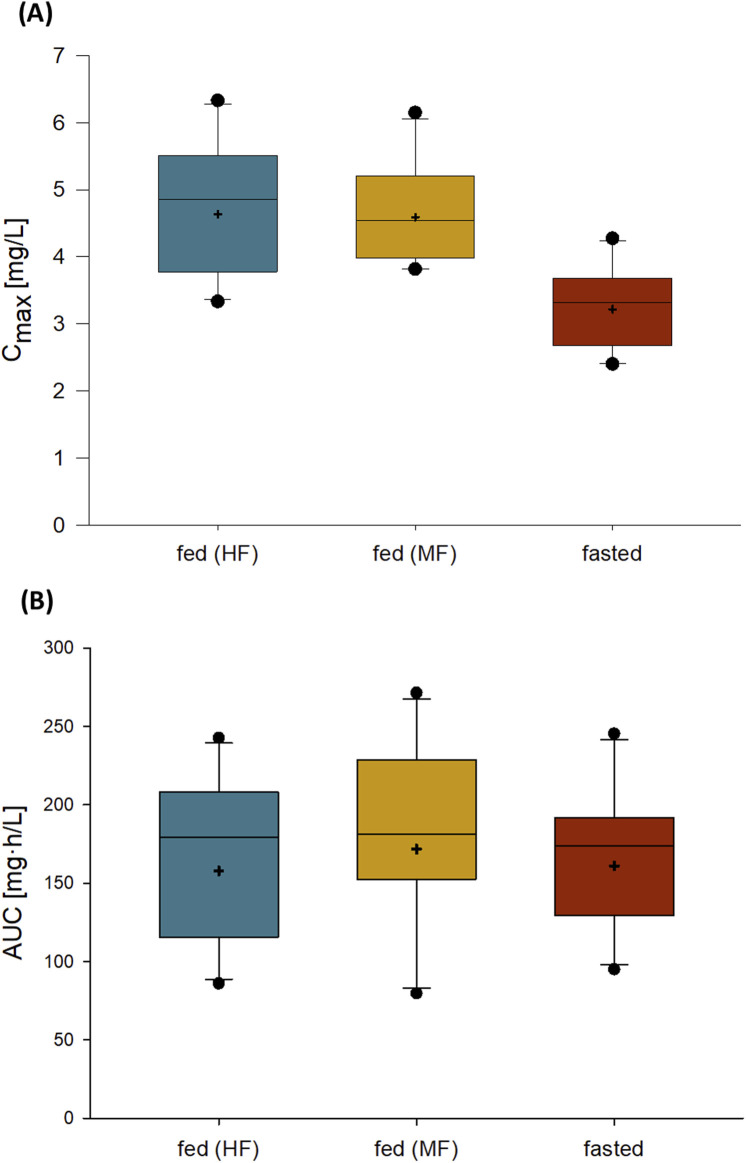
C_max_ **(A)** and AUC **(B)** of zabedosertib in plasma after single oral administration of 120 mg zabedosertib in different fed states and fasted states (FE/abs.BA study). Box: 25th to 75th percentile; horizontal line: median; whiskers: 10th to 90th percentile; cross: geometric mean; any value more extreme is plotted separately (N = 10). Abbreviations: AUC, area under the concentration–time curve; C_max_, maximum observed drug concentration; FE/abs.BA, food-effect and absolute bioavailability; HF, intake of zabedosertib after a high-fat, high-calorie meal; MF, intake of zabedosertib after a moderate-fat, moderate-calorie meal.

Administration of 120 mg zabedosertib after consumption of a moderate-fat, moderate-calorie meal or a high-fat, high-calorie meal resulted in an approximately 1.4-fold C_max_ compared with administration in the fasted state (Table 6; Figure 5). The AUC, in contrast, was similar under fed and fasted conditions, as were the time to reach maximum plasma concentrations and the elimination half-life. Variability was low for both AUC and C_max_. These observations are in line with results from the SAD study.

#### 3.3.5 Population-pharmacokinetic analysis (SAD and MAD)

The observed nonlinearity in the PK of zabedosertib after a single-dose administration, which resulted in a less-than-proportional increase in exposure with increasing dose, could be adequately described by assuming that the relative bioavailability decreases with increasing dose. As after multiple dosing a lower accumulation ratio was observed than expected based on the terminal half-life determined after a single dose, the model was adapted to capture this nonlinearity.

Zabedosertib binds to both human serum albumin and alpha-acid glycoprotein (AGP) *in vitro* with dissociation constants (K_d_) of 178 and 0.4 µM, respectively. This led to the hypothesis that capacity-limited binding to these proteins could change the unbound concentration over time, resulting in a change in both the total clearance and the total volume of distribution. As the affinity of zabedosertib for AGP is considerably higher and its concentration in plasma is typically lower (∼15 µM) than that of human serum albumin (∼600 µM), AGP binding was expected to have a larger influence on the unbound concentrations and the modeling focused on capacity binding to AGP.

### 3.4 Pharmacodynamics

**FIGURE 6:**
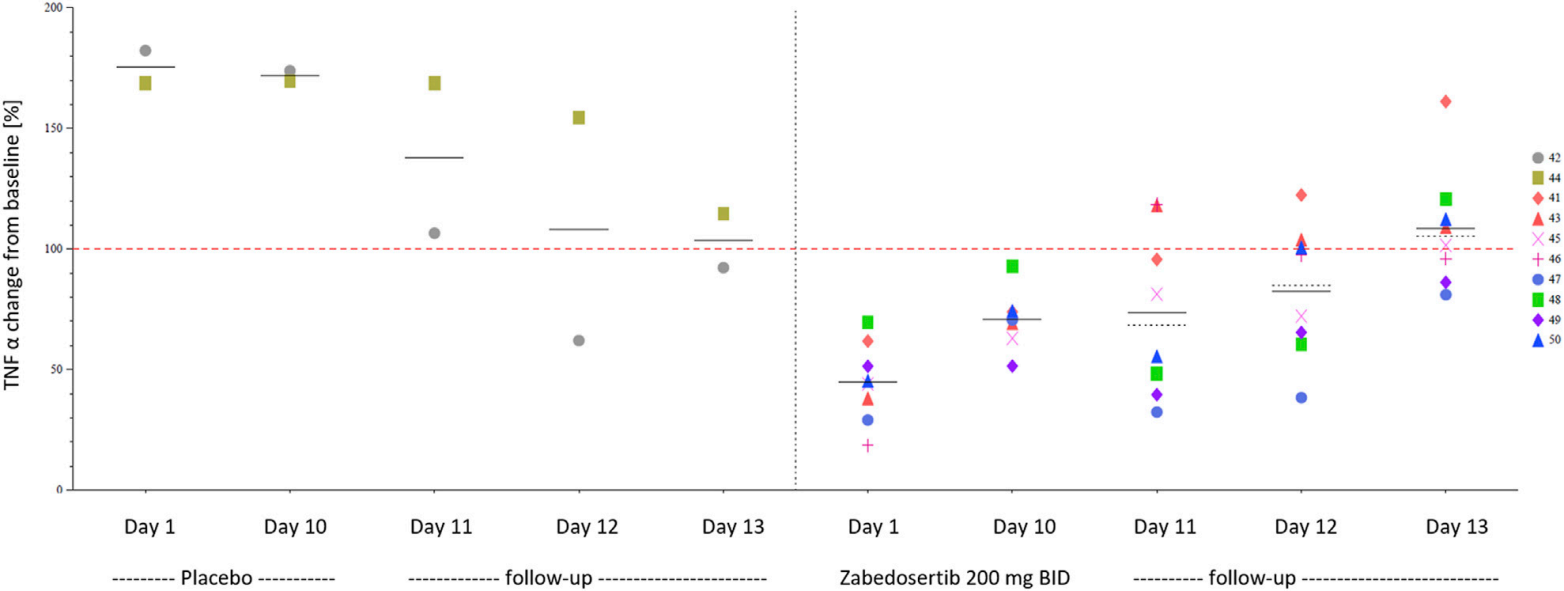
TNFα during and after 10-day treatment with zabedosertib 200 mg twice daily or placebo (MAD study, individual data). Different symbols and numbers indicate individual participants. Abbreviations: BID, twice daily; MAD, multiple ascending dose; TNFα, tumor necrosis factor α.

Single-dose administration of zabedosertib to healthy men led to a transient reduction in LPS-mediated cytokine release (6 h post-dose) in the *ex vivo* whole-blood assay (Table 7), which resolved within approximately 3 days (Figure 6). The effect was most pronounced for TNFα (6-h stimulation protocol, 0.1 ng/mL LPS; see Supplementary Section 1.4.3) and interferon gamma (24-h stimulation protocol, 100 ng/mL LPS). This was interpreted as an indication of target engagement on the enzyme IRAK4, which plays a central role in the LPS signaling due to its role in the signaling downstream of toll-like receptor 4. A dose-dependent complete or near-complete suppression of TNFα mean values was not observed.

**TABLE 7:** Exploratory pharmacodynamic biomarkers–cytokine secretion after *ex vivo* whole-blood LPS stimulation (SAD study).

		Zabedosertib								Placebo		Placebo split fed	
Biomarker		30 mg		120 mg		480 mg		480 mg split fed					
(pg/mL)		N = 5		N = 6		N = 6		N = 6		N = 6		N = 2	
TNFα^a^	Baseline	5243	±3283	3600	±2377	5217	±2114	5559	±1479	4932	±1302	6738	±3234
	Change	−1155	±1438	−418	±811	−238	±2385	−949	±1149	289	±250	−261	±2823
TNFα^b^	Baseline	805	±443	1119	±530	982	±555	1649	±1216	992	±476	932	±174
	Change	48	±353	−291	±215	−25	±292	−674	±986	−34	±226	105	±195
IL-1β^b^	Baseline	2088	±671	1989	±968	2257	±661	2155	±827	2262	±1369	3144	±1283
	Change	−116	±534	−513	±477	−597	±687	−698	±588	−166	±379	7	±100
IFN-γ^b^	Baseline	11,050	±8161	16,943	±11,632	24,237	±23,290	46,141	±77,342	8892	±5601	11,643	±9383
	Change	−2037	±8829	−4628	±5347	−2569	±25,230	−13990	±27,810	747	±1187	−2587	±7452
IL-6^b^	Baseline	15,138	±2870	16,911	±7022	15,003	±5875	14,974	±3163	14,512	±4308	20,444	±4169
	Change	−1357	±3708	−3025	±1579	−1285	±5228	−2201	±2220	14	±919	949	±4517

Data are mean ± standard deviation for baseline values and changes from baseline 6 h post-dose. Baseline for cytokine concentrations was defined as the median of all non-missing valid measurements before the administration of zabedosertib or placebo.

^a^ 6-h LPS stimulation (0.1 ng/mL).

^b^ 24-h LPS stimulation (100 ng/mL). Abbreviations: IFN-γ, interferon gamma; IL-…, interleukin … ; LPS, lipopolysaccharide; TNFα, tumor necrosis factor alpha.

**FIGURE 7:**
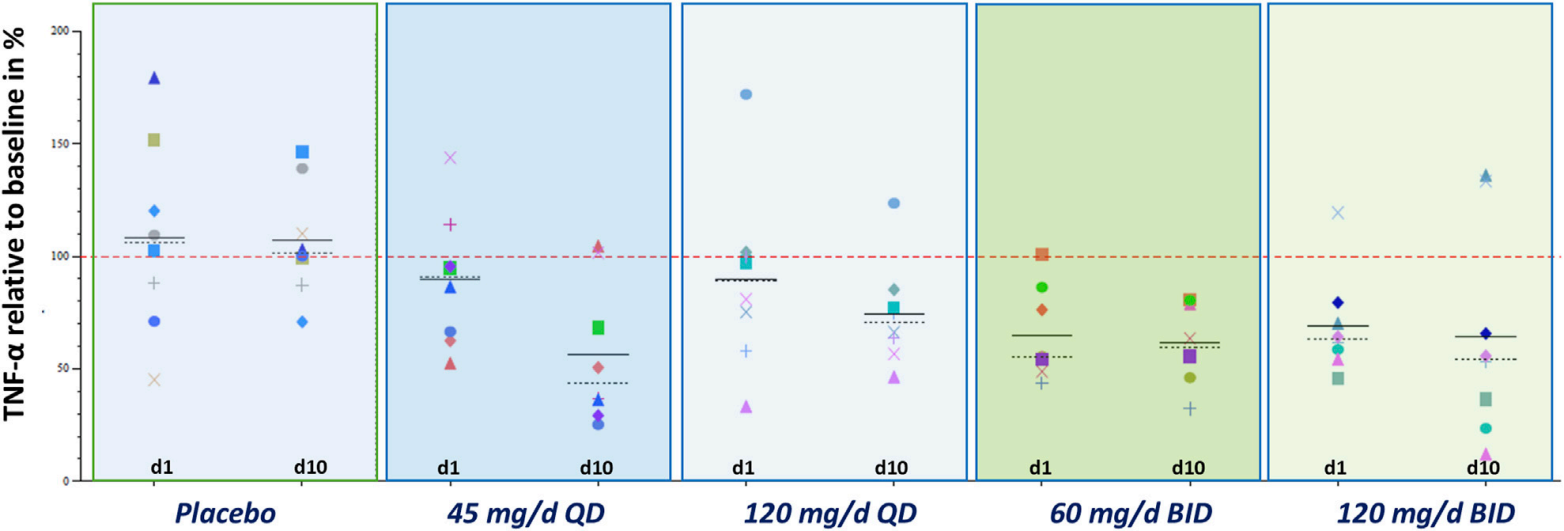
Exploratory pharmacodynamic biomarker analysis in whole blood: TNFα after stimulation with LPS (MAD study; change from baseline; individual data) TNFα release after 6-h LPS stimulation 0.1 ng/ml. The individual baseline value was defined as the median of all non-missing valid measurements prior to the first administration of study medication. Placebo data were pooled. Abbreviations: BID, twice daily; d … , treatment day … ; MAD, multiple ascending dose; QD, once daily; TNFα, tumor necrosis factor α.

Repeated administration of zabedosertib over 10 days led to a clear reduction in LPS-stimulated TNFα release in almost all zabedosertib-treated participants (Figure 7). The reduction seemed to increase with dose and treatment duration. After the end of treatment, the TNFα release reached the baseline level again within 3 days (Figure 6).

### 3.5 Target occupancy

**FIGURE 8:**
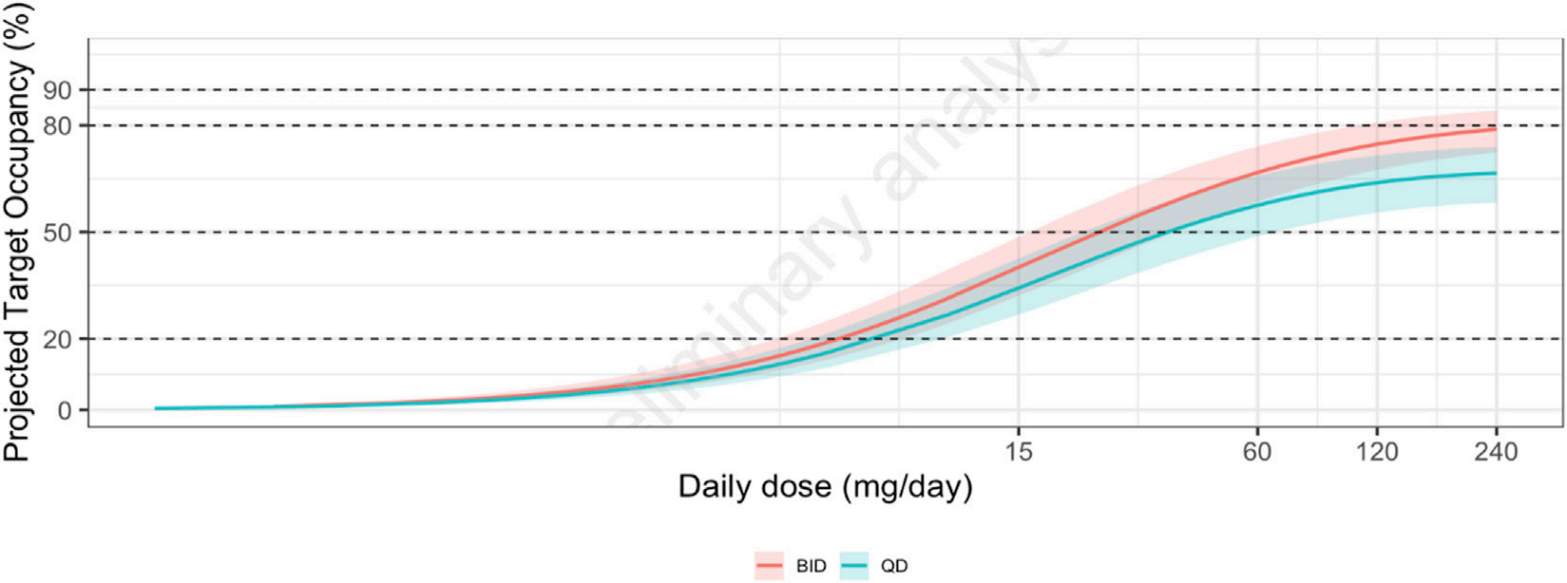
Relationship between zabedosertib dose and projected steady-state target occupancy at trough. Solid colored lines show the median projected target occupancy, and the shaded areas show the corresponding 95% confidence intervals based on the uncertainty in the IC_50_ for *in vitro* IL-6 release inhibition after resiquimod stimulation. Abbreviations: BID, twice daily; QD, once daily.

IC_50_ for IL-6 release inhibition after resiquimod stimulation in the *in vitro* human whole-blood assay was 86 (60–122) nM (mean and 95% CI). With the assumption that this concentration would result in 50% IRAK4 occupancy by zabedosertib in healthy volunteers, the estimated steady-state target occupancy at trough after 120 mg zabedosertib BID was on average 79% (72%–84%) (Figure 8). After the same daily dose given as a single dose, that is, 240 mg, the estimated steady-state target occupancy was clearly lower [67% (58%–74%)].

## 4 Discussion

### 4.1 Overall summary of results

The three early phase 1 studies of zabedosertib described in this publication revealed a favorable safety and tolerability and pharmacokinetic profile for zabedosertib. Single oral doses of zabedosertib of up to 480 mg and repeated oral doses up to 200 mg twice daily for 10 days (taken as tablets) were well tolerated in healthy male volunteers, without dose-limiting toxicities or severe infections.

The PK profile of zabedosertib is characterized by increasing concentrations until 2–3 h after dosing and a plateau until approximately 6 h post-dose. For doses >60 mg, a sub-proportional increase in exposure was observed, with an absolute bioavailability of 74% at a dose of 120 mg. The nonlinearity in the pharmacokinetics could be adequately described with a one-compartmental population-pharmacokinetic model with first-order elimination, dose-dependent bioavailability, and capacity-limited binding in plasma. Explorative biomarker analysis revealed signals for target engagement or pharmacodynamic effects by reduction in the LPS-stimulated release of TNFα in an *ex vivo* whole-blood assay. Target occupancy at a dose of 120 mg BID was estimated to be 79%.

### 4.2 Safety of zabedosertib

Across the three reported phase 1 studies, zabedosertib showed good safety and tolerability, without dose-limiting toxicities or severe infections in the healthy male volunteers. In accordance with the benign adverse event profile and favorable tolerability of the tested increasing doses of zabedosertib, vital signs, regular ECGs, and laboratory safety parameters were assessed without any signals or other noteworthy findings beyond those described above. This is in line with published data on other drugs inhibiting IRAK4, where no safety concerns in healthy volunteers or in patients were reported (Danto et al., 2019a⁠; Winkler et al., 2021). In contrast to an IRAK4-degrading molecule, no prolongations of QT-time in ECG were observed (Ackerman et al., 2023). Moreover, no signals for rhabdomyolysis were observed, which is in contrast to experiences with emavusertib (CA-4948) (a combined IRAK4/FLT3-inhibitor), for which a partial clinical (FDA)-hold was set in place due to a case of lethal rhabdomyolysis in an oncologic clinical study (treatment in^7^ combination with azacitidine or venetoclax).

At the time when the study protocols for the studies reported here were drafted, no such safety data were available, and based on the mode of action and a potentially immune-compromising effect (Picard et al., 2007), different AESIs were defined and helped to assess the benefit-risk ratio of zabedosertib while conducting the study. In the early phase 1 program of zabedosertib with treatment durations of up to 10 days, no signals for a clinically meaningful immune suppression were found. This is supported by the fast return of TNFα levels within 72 h observed in the *ex vivo* LPS whole-blood assay (data available for the 200-mg BID-dose group), which suggests a quick reconstitution of the innate immune function, providing no evidence for sustained immune suppression in the study settings. The data may also point toward a quick suspension of the drug effect by zabedosertib, unlike what can be observed with molecules degrading IRAK4 or covalent binders.

The overall good safety and tolerability of zabedosertib observed in the studies reported here is in line with data collected in an up to 12-week treatment time frame^8^ but cannot be predictive for longer-term treatment and, therefore, will have to be confirmed in a chronic treatment setting. A good prerequisite for the long-term use of zabedosertib is that there were no signals for gastrointestinal intolerability detectable across studies.

### 4.3 Pharmacokinetic properties of zabedosertib

The bioavailability of zabedosertib was greater when the drug was taken in the tablet form than when it was taken in the liquid form (both taken as single doses in the fasted state). One explanation could be that the excipient Polyethylene glycol (PEG) used for the liquid formulation decreases the small intestinal transit time, which resulted in a reduced absorption from the small intestine (Basit et al., 2002⁠; Lentini et al., 2016). Another explanation could be that zabedosertib from the liquid formulation rapidly precipitates in the stomach due to its low solubility in water. The observed time to maximum plasma concentrations of approximately 5 h for the 15-mg liquid-formulation dose was also distinctively longer than the 2 h determined for the 15-mg tablet formulation in the same participants. Drug precipitates might have redissolved to a certain extent. This could explain the slow increase and late maximum of zabedosertib plasma concentrations after the administration of zabedosertib as liquid formulation.

The assessment of a potential food effect on the pharmacokinetics of zabedosertib revealed an increase in C_max_ by a factor of approximately 1.4 under fed conditions, that is, when zabedosertib was administered after a high-fat or moderate-fat meal compared to fasted conditions. The extent of absorption (AUC), however, was unaffected by the intake of food. Likewise, the time to maximum plasma concentrations and the relatively long half-life (∼26 h) remained unaffected by the presence or absence of concomitant food intake. The food effect on C_max_ may be explained by an enhanced solubility of zabedosertib, resulting in an increased absorption rate shortly after drug intake.

Following multiple-dose administration over 10 days (tablet intake 30 min after a light meal), a lack of dose proportionality, that is, a decrease in dose-normalized PK parameter values (AUC, C_max_, and C_av_) with increasing dose, was observed at higher dose. Similarly, no dose proportionality was observed for PK parameters of unbound zabedosertib. The time course of the zabedosertib trough concentrations indicated that steady-state conditions in plasma were reached after 2–3 days. It can therefore be assumed that the multiple-dose PK parameters determined reflect steady-state conditions.

The C_max_ after multiple dosing increased by a factor of 1.05 [120 mg QD] to 1.27 [60 mg BID] compared to single dosing. The mean accumulation ratio for AUC increased by a factor of 1.04 [120 mg QD] to 1.42 [60 mg BID], with lower accumulation observed at higher doses. This is less than the theoretical value of 3.65 estimated from the half-life (∼26 h) and the length of the dosing interval (12 h BID). The sub-proportional accumulation is also reflected in the linearity factor, which decreased with increasing dose, also indicating nonlinearity in PK.

The geometric mean f_u_ of zabedosertib determined *ex vivo* in pre-dose plasma samples and in plasma samples of placebo-treated participants ranged between 1.44% and 4.00%, confirming the *in vitro* data. The unbound fraction decreased within 24 h post-dose to some extent and reached pretreatment values at 96 h post-dose. There was a considerable variability in the f_u_ data, as indicated by the broad 95% prediction interval. As zabedosertib binds in plasma with high affinity to AGP, the unbound fraction is dependent on the plasma concentration of this acute-phase protein. The geometric mean AGP concentration determined in all verum-treated participants (0.612 g/L; CV: 7.08%; fasted conditions) did not significantly change after treatment with zabedosertib (e.g., at 24 h post-administration 0.585 g/L; CV: 6.55%). Nevertheless, the AGP concentration ranged among individuals from 0.41 to 0.91 g/L in the corresponding pretreatment samples, indicating that different AGP concentrations could have contributed to a certain extent to the observed variability in the unbound fraction of zabedosertib. AGP concentrations can be elevated in certain inflammatory diseases (Jaurila et al., 2020⁠; Farrag et al., 2024⁠; Agra et al., 2017). Possibly, this could lead to higher total concentrations of zabedosertib. However, given the high interindividual variability of AGP, the effect is expected to be minor.

The PK of zabedosertib after both single and multiple dosing could be adequately described by a one-compartmental model with first-order elimination, a dose-dependent bioavailability, and by assuming that zabedosertib binds to a high-affinity and low-capacity binding partner or component in the plasma with a binding affinity of 0.4 µM, that is, with an affinity like the *in vitro* AGP-binding affinity. The estimated first-order dissociation rate has a half-life of approximately 12 h, which is relatively long compared to *in vitro* estimates of plasma protein binding, with dissociation half-lives of seconds. The binding capacity of 14.9 µM is estimated to be very close to the median AGP concentration in the reported studies (15.2 µM). In addition, the individual estimates of the binding partner concentrations show a weak positive correlation (Pearson correlation coefficient of 0.28) with the observed AGP concentrations. However, it should be noted that the binding to AGP is a model assumption and does not necessarily reflect actual biology. The dose-dependent bioavailability could also partly be explained by the fact that zabedosertib is lipophilic (log P 2.4) and has a low solubility in water. However, at a dose of 120 mg, zabedosertib has a high oral bioavailability (73.9%), along with a low clearance (0.551 L/h) and long half-life (∼26 h), as demonstrated in the microtracer study (FE/abs.BA study). In addition to limited water solubility and capacity-limited protein binding, which may impact the pharmacokinetics of zabedosertib, another underlying mechanism for the observed nonlinearity in PK might be target binding to certain tissue compartments or any combination of the mechanisms. Currently, no mass balance data are available to provide further insights. Whatever the underlying mechanisms may be, our current data indicate that dose increases above 120 mg zabedosertib BID, administered as immediate-release tablets, did not result in clinically relevant higher exposure.

Currently, data on the potency of several IRAK4 inhibitors that are in clinical development in humans have been published. The IRAK4 inhibitor zimlovisertib, for example, showed positive proof of concept in a phase 2b trial in patients with active rheumatoid arthritis (Danto et al., 2019b). This drug has one of the highest potencies with an IC_50_ of 0.2 nM (Danto et al., 2019b), whereas zabedosertib has an IC_50_ of 8 nM; the half-life of zimlovisertib after single dosing was shorter than that of zabedosertib, but it could be increased to 38.8 ± 21.1 h with the development of a modified-release formulation (Danto et al., 2019a). For zabedosertib, this would not be required as its half-life is ∼26 h, and BID dosing was needed to maximize exposure. Due to the BID dosing, long half-life, and the plateau-like concentration–time profile of zabedosertib, sufficiently high exposure over 24 h was achieved.

### 4.4 Pharmacodynamics and target occupancy

As demonstrated by Winkler et al. (2021), *in vitro* IL-6 inhibition correlates with IRAK4 target occupancy. Based on this assumption and the reported pharmacokinetics of 120 mg BID zabedosertib, the calculated target occupancy is ∼80%. These observations, together with the positive proof of concept for zimlovisertib, support the assumption that zabedosertib can be dosed sufficiently high to achieve a clinical effect. This is furthermore supported by the observed distinct PD effects in humans in a proof-of-mechanism study, which demonstrated a clear suppression of systemic inflammation and suppression of local inflammation following a systemic LPS challenge and a local (akin) imiquimod challenge (Jodl et al., 2024).

### 4.5 Limitations of the studies

In accordance with sponsor-internal standards in force at the time of the conduct of the phase 1 studies, only men were included although the envisaged indications of the drug include conditions that affect both men and women. Healthy women who would not benefit from participation in the studies were excluded from study participation for safety reasons because no reproductive toxicity studies had been conducted when the three studies were conducted and to control PK variability at this early stage of development. Furthermore, the vast majority of study participants were White/Caucasian although the protocols had no restrictions with regard to race and ethnicity. An intrinsic limitation of the three studies is the overall relatively small sample size of the individual dose groups and the exclusion of elderly study participants. Another limitation is that the phase 1 program in healthy volunteers covered only a treatment length of up to 10 days. Therefore, conclusions with regard to long-term safety and tolerability and predictions for a longer treatment time frame are limited.

Yet another limitation of the studies might be that the measured systemic exposures may not represent the desired exposures at the site of action (target tissue) of the respective target indication. Furthermore, an *ex vivo* whole-blood assay was performed in this phase 1 program to reflect the pharmacodynamic effects of zabedosertib, but from this assay, no dose decision could be derived for phase 2 due to a certain “floor effect” in cytokine suppression. Therefore, IL-6 correlation with IRAK4 target occupancy was used as substitute, and a separate proof-of-mechanism study was performed (Jodl et al., 2024).

### 4.6 Implications of findings for future research

The above-described exposure limitations define, from a PK perspective, 120 mg BID as the highest feasible dose for future studies with zabedosertib. The very good safety and tolerability of this dose over up to 10 days and the high projected target coverage, which is expected to lead to a clinical effect in the relevant target indications, suggest advancing zabedosertib further in its development.

### 4.7 Conclusion

Based on the projected target occupancy and the favorable pharmacokinetic and safety profile, as well as distinct pharmacodynamic effects in a proof-of-mechanism study, zabedosertib 120 mg twice daily was selected for further clinical development in patient studies.

## Data Availability

The data analyzed in this study are subject to the following licenses/restrictions: the data analyzed are not publicly available for confidentiality reasons. Requests to access these datasets should be directed to Maximilian Feldmüller, maximilian.feldmueller@bayer.com.

## Footnotes

1 This study comprised two independent parts, of which only the first part is reported here. The second part, a multiple-dose safety, tolerability, PK, and PD study in patients with psoriasis, is planned to be reported separately.

2 This study comprised two independent parts, of which only the first part is reported here. The second part, a drug–drug interaction study with methotrexate, is planned to be reported in a separate publication. This publication will also include an evaluation of the CYP3A4-interaction potential of zabedosertib.

3 The studies described in this publication are planned to be part of the clinical pharmacology package in a future regulatory submission. At the time this manuscript was written, zabedosertib was in the phase 2 status, and the assessment of the optimal target indication for further clinical development was ongoing. A manuscript describing the results of a Ph2a proof-of-concept trial in atopic dermatitis (https://clinicaltrials.gov/: ID: NCT05656911) is in preparation.

4 In the MAD study, participants additionally received midazolam at a subtherapeutic dose to allow an early assessment of the drug–drug interaction potential of zabedosertib. The results are planned to be reported in a separate publication.

5 In addition, urine was collected in the SAD study for PK analyses.

6 The same method was used for the determination of zabedosertib in urine and for explorative metabolite analyses, the results of which are not reported here. The amount of drug excreted in urine within 24 h post-dose was <3% of the administered dose on average in the SAD study.

7
https://www.prnewswire.com/news-releases/curis-announces-fda-partial-clinical-hold-for-takeaim-leukemia-study-of-emavusertib-ca-4948-301516446.html https://investors.curis.com/2022-04-11-Curis-Announces-FDA-Partial-Clinical-Hold-for-TakeAim-Lymphoma-Study-of-Emavusertib-CA-4948

8 The abstract of the results of this proof-of-concept study in moderate-to-severe atopic dermatitis, 22158, has been accepted for the EADV Annual Meeting 2024.

## Abbreviations

AESI, treatment-emergent adverse event of special interest; AGP, alpha-acid glycoprotein; AUC, area under the plasma concentration–time curve from zero to infinity; AUC(0-x), area under the plasma concentration–time curve from zero to time x; BID, twice daily; C_av_, average concentration within the dosing interval; CI, confidence interval; CL, total body clearance after IV administration; C_max_, observed maximum drug concentration in plasma; F, absolute oral bioavailability; FE/abs.BA, food-effect/absolute bioavailability (study); f_u_, unbound fraction; IC_50_, half-maximum inhibitory concentration; IL, interleukin; IRAK4, interleukin-1 receptor-associated kinase 4; IV, intravenous; LPS, lipopolysaccharide; MAD, multiple ascending dose; md, multiple dose; PD, pharmacodynamic(s); PK, pharmacokinetic(s); QD, once daily; SAD, single ascending dose; TEAE, treatment-emergent adverse event; TNFα, tumor necrosis factor alpha; V_ss_, volume of distribution at steady state.
