# Supplementary material for: Zabedosertib, a novel interleukin-1 receptor-associated kinase-4 inhibitor, shows a favorable pharmacokinetic and safety profile across multiple phase 1 studies
Source: Front Pharmacol. 2025 May 30;16:1521505. doi: 10.3389/fphar.2025.1521505 (PMC12163019; doi:10.3389/fphar.2025.1521505)
Supplement: Supplementary file 1 [file DataSheet1.pdf]

frontiers

Supplementary Material

Contents

1 Supplementary Material: Materials and Methods 3
1.1 Study details – overview 4
1.2 Study participants: Inclusion/exclusion criteria 7
1.2.1 Inclusion/exclusion criteria SAD study 7
1.2.2 Inclusion/exclusion criteria MAD study 10
1.2.3 Inclusion/exclusion criteria FE/abs.BA study 12
1.3 Study procedures 15
1.3.1 FE/abs.BA study: Composition of meals served before drug administration 15
1.3.2 Sampling schedules 16
1.4 Bioanalytical methods 17
1.4.1 Determination of zabedosertib in plasma 17
1.4.2 Determination of unbound fraction of zabedosertib 19
1.4.3 Investigation of explorative pharmacodynamic biomarkers 20
1.5 PopPK analysis 21
1.6 Target occupancy – IC_{50} for IL-6 inhibition 27

2 Supplemental Material: Study Participants 28
2.1 SAD study: Participant demographics and other baseline characteristics 29
2.2 MAD study: Participant demographics and other baseline characteristics 30
2.3 FE&BA study: Participant demographics and other baseline characteristics 31
2.4 SAD study: Participant disposition 32
2.5 MAD study: Participant disposition 33
2.6 FE/abs.BA study: Participant disposition 34

3 Supplemental Material: Safety 35
3.1 SAD study: Overall summary of treatment-emergent adverse events 36
3.2 SAD study: Treatment-emergent adverse events by primary MedDRA system organ class and preferred term 37
3.3 MAD study, single-dose period: Overall summary of treatment-emergent adverse events 39
3.4 MAD study, single-dose period: Treatment-emergent adverse events by MedDRA system organ class and preferred term 40
3.5 MAD study, multiple-dose period: Overall summary of treatment-emergent adverse events 41
3.6 FE/abs.BA study: Overall summary of treatment-emergent adverse events 42
3.7 FE/abs.BA study: Treatment-emergent adverse events 43

4 Supplemental Material: Pharmacokinetics 45
4.1 SAD study: Pharmacokinetic parameters of zabedosertib (total) in plasma, all dose groups 46
4.2 SAD study: Assessment of dose proportionality of zabedosertib 15–480 mg taken as tablets under fasting conditions 47

4.3 SAD study: Geometric mean unbound fraction (fu) of zabedosertib in plasma [%] after single oral administration of 5 mg to 480 mg zabedosertib and placebo under fasting conditions 48
4.4 SAD study: Relationship between zabedosertib plasma concentration and *ex-vivo*-determined unbound fraction of zabedosertib – individual data of all zabedosertib- and placebo-treated study participants 49
4.5 MAD study, single-dose period: PK parameters of zabedosertib (unbound) 50
4.6 MAD study, multiple-dose period: Geometric mean zabedosertib plasma concentration–time curves (±SD) obtained during repeated administration of zabedosertib tablets under fed conditions........ 51
4.7 MAD study, multiple-dose period: PK parameters of zabedosertib (total) obtained after repeated oral administration of zabedosertib to healthy young men 52
4.8 FE/abs.BA study: Mean [$^{13}C_6$]-zabedosertib plasma concentration–time curves obtained during and after a 15-minute intravenous infusion of 0.1 mg [$^{13}C_6$]-zabedosertib 53
4.9 SAD study: Assessment of the impact of drug formulation, concomitant food intake and dose splitting 54

1 Supplementary Material: Materials and Methods

1.1 Study details – overview

		SAD study	MAD study [a]	FE/abs. BA study [a]
1	ClinicalTrials.gov registration no.	NCT03054402	NCT03493269	NCT03244462
2	EudraCT registration no.	2016-002668-15	2017-001817-10	2016-004393-18
3	Relevant ethics committee	Ethik-Kommission des Landes Berlin, Berlin, Germany	Ethik-Kommission des Landes Berlin, Berlin, Germany	METC, Stichting BeBo, Assen, The Netherlands
4	Study center(s)	CRS Clinical Research Services Berlin GmbH, Berlin, Germany [b]	Charité Research Organisation GmbH, Berlin, Germany; Parexel GmbH, Berlin, Germany [b]	PRA Health Sciences [now ICON Plc], Groningen, The Netherlands
5	Study period (first in - last out)	Feb - Nov 2017	Apr 2018 – Sep 2020	Aug 2017 – Feb 2018
6	Study objectives<br>- primary	To investigate safety, tolerability and PK of ascending single oral doses of zabedosertib (first-in-human study)	To investigate safety, tolerability and PK of ascending repeated oral doses of zabedosertib	To investigate possible food effects on the PK of zabedosertib tablets and to determine the absolute bioavailability of zabedosertib
7	- other	To determine the relative bioavailability of the solid dosage form vs. a liquid form; to investigate exploratory PD biomarkers	To explore PD biomarkers (TNFα); to explore the CYP3A4-interaction potential of zabedosertib using midazolam as the probe substrate [c]	
8	Study design	Single-center, double-blind, sponsor-unblinded, randomized, placebo-controlled parallel-group study with ascending single doses (Manuscript, Figure 1A)	Randomized, placebo-controlled, double-blind single- and multiple-ascending-dose study (Manuscript, Figure 1B)	Open-label, randomized, 3-period, 2-sequence cross-over study (Manuscript, Figure 1C)
9	Participants	Healthy men, 18-50 years of age (inclusive) (SUPPL. MATERIAL 1.2)		
10	- Planned	96 participants (12 x 8) [d]	50 (5 x 10)	10
	- Treated	70 (zabedosertib: 52; PLC: 18)	50 (zabedosertib 40; PLC 10)	10 (5 per sequence)
	- Excluded from analysis [e]	None<br>(SUPPL. MATERIAL 2.4)	None<br>(SUPPL. MATERIAL 2.5)	None<br>(SUPPL. MATERIAL 2.6)
11	Randomization (verum : placebo)	6:2 in each dose group	8:2 in each dose group	1:1 to one of two treatment sequences
12	Blinding	Zabedosertib and placebo tablets were of identical appearance and smell.		N/A (open label)
13	Investigational products [f]	Zabedosertib immediate-release tablets; liquid service form for oral administration	Zabedosertib immediate-release tablets	Zabedosertib immediate-release tablets; [$^{13}C_6$]-zabedosertib solution for IV infusion (microtracer)

Continued next page

		SAD study	MAD study [a]	FE/abs. BA study [a]
14	Mode of administration	Oral, fasting or shortly after a standardized meal	Oral, shortly after a light meal	Oral (tablets) and by 15-min IV infusion (microtracer dose)
15	Dosage and treatment schedule	Single dose or split dose [g] of 5, 15, 30, 60, 120, 240 or 480 mg zabedosertib or placebo (Manuscript, Figure 1A)	Zabedosertib (45 mg QD, 120 mg QD, 60 mg BID, 120 mg BID, and 200 mg BID) or placebo as a single dose in one study period and over 10 days in the other with 2-week washout;[h] midazolam (1 mg as oral solution; ratiopharm, Germany) alone at day –1, and in combination with zabedosertib or placebo at day 10 (Manuscript Figure 1B)	3 single oral doses of 120 mg zabedosertib with 2-week washout between doses; plus an IV microdose of 0.1 mg $[^{13}C_6]$-zabedosertib on top of the oral dose taken in fasted state, shortly before the expected C_{max} of the oral dose (Manuscript, Figure 1C)
16	Concomitant food	Zabedosertib intake in fasted or fed state	Zabedosertib intake 30 min after the start of a light meal	Zabedosertib intake in fasted state in period 1 and following a high-fat, high-calorie meal in period 2 or vice versa; intake in period 3 following a moderate-fat, moderate-calorie meal (for meal compositions, see SUPPL. MATERIAL 1.3.1)
17	Concomitant medication	Use of concomitant medication was not planned; concomitant medication suspected to bias the study results was not permitted.		
18	Procedures and assessments	Screening visit, 1 or 2 treatment periods (Manuscript, Figure 1A), end-of-study visit 18-25 days after last dose	Screening visit, a 10-day multiple-dose period and a single-dose period (Manuscript, Figure 1B), end-of-study visit 18-22 days after last dose	Screening visit, 3 treatment periods (Manuscript, Figure 1C), end-of-study visit (14-21 d after last dose)
		Safety assessments;[i] blood sampling for PK, plasma protein-binding and exploratory biomarker analyses (SUPPL. MATERIAL 1.3.2); urine collection for PK over 24 h	Safety assessments;[i] blood sampling for PK and exploratory biomarker analyses (SUPPL. MATERIAL 1.3.2)	Safety assessments;[i] blood sampling for PK analyses (SUPPL. MATERIAL 1.3.2)
19	Primary variables			
	- Safety	Frequency and severity of treatment-emergent adverse events	Frequency and severity of treatment-emergent adverse events	---
	- Pharmacokinetics	AUC and C_{max} of zabedosertib	$AUC(0\text{-}24)_{md}$ or $AUC(0\text{-}12)_{md}$ of zabedosertib for QD and BID dosing, $C_{max,md}$ and C_{av}	AUC and C_{max} of zabedosertib after oral administration; CL and V_{ss} of labeled zabedosertib after IV administration; absolute bioavailability
20	Other variables	---	TNFα release	---

Continued next page

		SAD study	MAD study [a]	FE/abs. BA study [a]
21	Bioanalytics	Zabedosertib (plasma, urine): LC-MS/MS [j]<br>PD biomarker: ex vivo functional assay (whole-blood LPS stimulation)	Zabedosertib: LC-MS/MS [j]<br>PD biomarker: ex vivo functional assay (whole blood LPS stimulation)	Zabedosertib: LC-MS/MS
22	Pharmacokinetic evaluation	Noncompartmental analysis	Noncompartmental analysis	Noncompartmental analysis
23	Population-pharmacokinetic modeling	See Manuscript, Section 2.10		N/A
24	Statistical analysis	Exploratory analyses; see Manuscript, Section 2.9	Exploratory analyses; see Manuscript, Section 2.9	Exploratory analyses; see Manuscript, Section 2.9

[a] Details of part 2 of the study are not included in this table.

[b] Safety laboratory tests were performed at a central laboratory (SYNLAB Analytics & Services Germany GmbH).

[c] Investigations of the CYP3A4 interaction potential of zabedosertib are planned to be reported in a separate paper.

[d] Three of the groups planned were canceled (single-dose administration of 720 mg, 1080 mg and 1440 mg) because the increase in dose from 240 mg to 480 mg (both administered as split doses) did not lead to an increase in exposure.

[e] None of the participants was reassigned to another group for analysis.

[f] All drug products were produced by Bayer AG, Germany, unless otherwise indicated. The same immediate-release tablet formulation (at different strengths) was used in all studies. Only in the MAD study, group 5, a newly developed 200 mg tablet formulation was used because the exposure of zabedosertib at the preceding dose level was lower than predicted.

[g] Two times half the dose, 12 h apart.

[h] On the 10th day, the PK profiling day, only the morning dose was administered in the BID groups.

[i] Safety assessments: adverse event documentation, standard clinical laboratory tests, standard immune parameters, infection markers, physical examinations, vital signs, electrocardiograms.

[j] In addition, exploratory metabolite analyses were conducted (not reported here).

Abbreviations: BID, twice daily; IV, intravenous; LC-MS/MS, high-pressure liquid chromatography and tandem mass spectrometric detection; LPS, lipopolysaccharides; md, multiple dose; N/A, not applicable; QD, once daily; PD, pharmacodynamic(s); PK, pharmacokinetic(s); PLC, placebo. For abbreviations of pharmacokinetic parameters, see the list of abbreviations in Section 6 of the manuscript.

1.2 Study participants: Inclusion/exclusion criteria

1.2.1 Inclusion/exclusion criteria SAD study

Inclusion criteria

Subjects who met all of the following criteria were eligible for inclusion into the study:

1. The informed consent was signed before any study specific tests or procedures were done
2. Healthy male subject
3. Age: 18 to 50 years (inclusive) at the first screening visit
4. Body mass index (BMI): 18.5 ≤BMI ≤30 kg/m²
5. Male subjects and their female partners of childbearing potential were required to use an accepted method of contraception for the duration of the study. This applied for the time period between signing of the informed consent form and up to 30 days after the last administration of study drug.

   The definition of adequate contraception was based on the judgment of the investigator and on local requirements.

   Acceptable methods of contraception included, but were not limited to, (i) condoms (male or female) with or without a spermicidal agent; (ii) diaphragm or cervical cap with spermicide; (iii) intra-uterine device; (iv) hormone-based contraception

   Subjects had to agree to utilize two reliable and acceptable methods of contraception simultaneously.
6. Confirmation of the subject's health insurance coverage prior to the first screening examination / visit
7. Ability to understand and follow study-related instructions

Exclusion criteria

Subjects who met any of following criteria were not eligible for inclusion into the study:

Medical and surgical history

1. Incompletely cured pre-existing diseases for which it could be assumed that the absorption, distribution, metabolism, elimination and effects of the study drugs would not be normal
2. Known gastrointestinal (GI) disorders (e.g. stomach ulcers, duodenal ulcers, GI bleeding) or inflammatory bowel disease (e.g. Crohn's disease, ulcerative colitis)
3. Relevant diseases within the last 4 weeks prior to the first study drug administration
4. Febrile illness within 4 weeks before the first study drug administration
5. Known hypersensitivity to the study drugs or components of the preparations
6. Known severe allergies, non-allergic drug reactions, or multiple drug allergies
7. Known cardiovascular disorders, e.g. presence or history of prodroma of a thrombosis, valvular heart disease, atrial fibrillation, cardiac dysfunction
8. Known or suspected malignant tumors or carcinoma in situ (including history of malignant tumors, with a status after treatment)
9. Known or suspected diseases of the respiratory tract, e.g. asthma, chronic obstructive pulmonary disease, bronchiectasis, or cystic fibrosis
10. Known or suspected immunodeficiency, e.g. history of recurrent and/ or severe infections (e.g. bacterial arthritis, cellulitis, chronic sinusitis, furunculosis, meningitis, organ abscesses, osteomyelitis, otitis, pneumonia and sepsis); eczema (atopic dermatitis)

11. Liver diseases: existing or previous, acute or chronic progressive liver diseases, e.g., disturbances of the bilirubin excretion (Dubin-Johnson and Rotor syndromes), cholecystectomy; disturbances of the bile secretion and the flow (cholestasis, also history thereof); presence or history of liver tumors (benign or malignant); history of viral hepatitis
12. Relevant kidney diseases (e.g. glomerulonephritis), or renal injury associated with multisystem diseases/disorders (e.g. systemic lupus erythematosus, diabetic nephropathy); a history of a single episode of uncomplicated nephrolithiasis did not prevent participation
13. Known metabolic disorders (e.g. diabetes mellitus, severe hypertriglyceridemia)
14. Clinically significant depression (current or in the last year)
15. Known current thyroid disorders which require treatment (subjects with an euthyroid struma who did not need any treatment could participate)
16. History of known or suspected tuberculosis

Medication, drug use and special behavioral patterns

17. Regular use of therapeutic or recreational drugs, e.g. carnitine products, anabolics, high-dose vitamins
18. Use of systemic or topical medicines or substances which opposed the study objectives or which might have influenced them within 4 weeks before first study drug administration, e.g. an investigational drug; any drug known to induce liver enzymes (e.g. dexamethasone, barbiturates, rifampicin, anticonvulsants, griseofulvin, St. John's Wort [Hypericum perforatum]).
19. Repeated use of drugs during 1 week before first study drug administration which might have affected absorption (e.g. laxatives, loperamide, metoclopramide, antacids, H2-receptor antagonists)
20. Use of drugs during 4 weeks before first study drug administration which might have affected the immune system (e.g. glucocorticoids, NSAIDs, IL-1R antagonists, TNFα inhibitors)
21. Smoking (former smokers who had stopped smoking at least 3 months before the first screening visit could be included)
22. Regular daily consumption of more than 800 mL of usual beer or the equivalent quantity of approximately 40 g of alcohol in another form
23. Intake of alcohol within 48 h before first study drug administration
24. Special diets preventing the subjects from eating the standard meals during the study, e.g. strict vegetarian or low caloric diet
25. Regular daily consumption of more than 1 L of xanthin-containing beverages, e.g. coffee, tea, cacao
26. Intake of xanthin-containing beverages within 48 h before first study drug administration
27. Intake of foods or beverages containing grapefruit, pomelo and Seville oranges within 7 days before first study drug administration until last sample for PK measurement was collected
28. Donation of blood or plasmapheresis after signing the informed consent form

ECG, blood pressure, pulse rate

29. Clinically relevant findings in the ECG such as a second- or third-degree atrioventricular (AV) block, prolongation of the QRS complex over 120 msec or clinically relevant QTcB-prolongation over 450 msec
30. Systolic blood pressure below 100 or above 145 mmHg (after at least 10 min in supine position)

31. Diastolic blood pressure below 50 or above 95 mmHg (after at least 10 min in supine position)
32. Pulse rate below 45 or above 95 beats / min

Physical examination

33. Clinically relevant findings in the physical examination

Laboratory examination

34. Positive results for hepatitis B virus surface antigen (HBsAg), hepatitis C virus antibodies (anti-HCV), human immunodeficiency virus antibodies (anti-HIV 1 and 2), mycobacterium tuberculosis (positive whole-blood IFN-γ test)
35. Positive urine drug screening
36. Clinically relevant deviations of the screened laboratory parameters from reference ranges, especially for high-sensitivity C-reactive protein (hsCRP) and HLA-DR expression on monocytes

Other

37. Participation in another clinical study during the preceding 2 months (last treatment from previous study to first treatment of new study)
38. Scheduled (elective) surgery / planned hospitalization / dental treatment after signing the informed consent form until end of study
39. Exclusion periods from other studies or simultaneous participation in other clinical studies
40. Subject was in custody by order of an authority or a court of law
41. Criteria which in the opinion of the investigator precluded participation for scientific reasons, for reasons of compliance, or for reasons of the subject’s safety
42. Previous assignment to treatment (e.g. randomization) during this study (allowing previously randomized subjects to be re-included into the study may have led to bias)
43. Close affiliation with the investigator (e.g. a close relative) or persons working at the study site
44. Subject was an employee of Bayer Pharma AG or Bayer AG or CRS Group
45. Unable/unwilling to comply with study restrictions

1.2.2 Inclusion/exclusion criteria MAD study

Inclusion criteria

Subjects had to fulfill all of the following criteria to be included in the study:

1. Signed and dated informed consent
2. Healthy male subjects
3. 18 to 50 years of age (inclusive)
4. Good health determined by medical history, physical examination, vital signs, ECG, and laboratory tests at screening
5. No signs of increased susceptibility to severe or chronic infections
6. Body mass index (BMI) above or equal 18.5 and lower or equal 30 kg/m^2
7. Body weight above or equal 50 kg
8. If sexually active with a female partner of childbearing potential, non-sterilized males had to agree to practice adequate methods of contraception. This applied for the time period between signing of the informed consent form and up to 30 days after the last administration of the study drug(s)..
9. Able to communicate well with the investigator, to understand and comply with the requirements of the study

Exclusion criteria

Subjects had to be excluded from the study if they met any of the following criteria:

1. Use of other investigational drugs at the time of enrollment, or within 5 half-lives of enrollment, or within 30 days, whichever is longer
2. Previous assignment to treatment during this study
3. Subjects with a medical history of increased frequency of infections, immunodeficiency diseases, with recent febrile diseases and anamnestic and/or laboratory signs of an impaired immune status (clinically relevant changes in hematologic parameters or reduced HLA-DR on monocytes) or latent infections (hepatitis B, hepatitis C, tuberculosis, and human immunodeficiency virus (HIV)) were excluded from the study.
4. Any active or ongoing chronic infectious disease
   a) Any infection requiring hospitalization, parenteral antimicrobial therapy within 60 days, or as otherwise judged to be an opportunistic infection or clinically significant by the investigator, within the past 6 months
   b) Infected joint prosthesis at any time with the prosthesis still *in situ*
   c) Recurrent (more than one episode) herpes zoster or disseminated (a single episode) herpes zoster or disseminated (a single episode) herpes simplex
5. Any disorder which, in the opinion of the investigator, might have jeopardized subject`s safety or compliance with the protocol
6. Any clinically relevant abnormal findings in medical history and physical examination at screening or Study Day -1 which in the opinion of the investigators, could have put the subject at risk because of his/her participation in the trial or provided difficulties in interpreting the trial data
7. Clinically relevant findings in the ECG such as a second- or third-degree atrioventricular (AV) block, prolongation of the QRS complex over 120 ms or clinically relevant QTcB (QT interval frequency-corrected according to Bazett’s formula) -prolongation over 450 ms

8. Systolic blood pressure below 100 or above 140 mmHg (after at least 10 min in supine position) at screening
9. Diastolic blood pressure below 50 or above 90 mmHg (after at least 10 min in supine position) at screening
10. Pulse rate below 50 or above 90 beats / min
11. Clinically relevant deviations of the screened laboratory parameters from reference ranges, especially for ALT (alanine aminotransferase) and AST (aspartate aminotransferase) (>1 x upper limit of normal [ULN]), Glomerular Filtration Rate < 90 mL/min, high-sensitivity C-reactive protein (hsCRP > 5 mg/L) and HLA-DR expression on monocytes at screening (Breithaupt-Groegler et al. 2017)
12. Whole blood or red blood cell donation, or any blood loss > 500 mL within 2 months prior to screening
13. History of hypersensitivity to any of the components of the study drug (e.g. lactose)
14. History of malignancy or obligatory precancerous condition of any organ system (other than localized basal cell carcinoma of the skin), treated or untreated, within the past 5 years, regardless whether there was evidence of local recurrence or metastases
15. History of drug or alcohol abuse within the 12 months prior to dosing, or evidence of such abuse as indicated by the laboratory assays conducted during screening and/or first baseline
16. Receipt of live or attenuated vaccine 90 days prior to the first dosing
17. History of tuberculosis (TB) or active or latent tuberculosis (as indicated by a positive result for QuantiFERON test)
18. Abuse of therapeutic or recreational drugs, e.g. carnitine products, anabolics, high-dose vitamins
19. Use of drugs during 4 weeks before first study drug administration which might suppress the immune system (e.g. glucocorticoids, IL-1R antagonists, TNFα inhibitors)
20. Any significant gastrointestinal disease or surgery resulting in malabsorption (cholecystectomy should be permitted)
21. Individuals with scheduled (elective) surgery, planned hospitalization and dental treatment are excluded from participation
22. Vulnerable subjects who are, e.g., institutionalized due to regulatory or juridical order, dependent on sponsor, site or investigator or not able to consent, respectively.

References:

Breithaupt-Groegler K, Coch C, Coenen M, Donath F, Erb-Zohar K, Francke K, et al. Who is a 'healthy subject'? Consensus results on pivotal eligibility criteria for clinical trials. Eur J Clin Pharmacol. 2017 Apr;73(4):409-16

1.2.3 Inclusion/exclusion criteria FE/abs.BA study

Inclusion criteria

Subjects who met all of the following criteria were eligible for inclusion into the study:

1. Signature of the informed consent form before any study specific tests or procedures were performed.
2. Male; healthy according to complete medical history, physical examination, vital signs, 12-lead ECG and clinical laboratory tests as listed in the exclusion criteria given below.
3. Age 18–50 years (inclusive) at the first screening visit.
4. Body mass index: $\geq$18 kg/m² and $\leq$30 kg/m².
5. Sexually active men had to agree to practice adequate methods of contraception (protection). This applied for the time period between signing of the informed consent form and up to 90 days after the last administration of the study drugs.
6. Ability to understand and follow study-related instructions.

Exclusion criteria

Subjects who met any of following criteria were not eligible for inclusion into the study:

Medical and surgical history:

1. Incompletely cured pre-existing diseases potentially associated with abnormal absorption, distribution, metabolism, elimination and effects of the study drugs.
2. Known gastrointestinal (GI) disorders (e.g. chronic diarrhea, stomach and duodenal ulcers, GI bleeding) or inflammatory bowel disease (e.g. Crohn's disease, ulcerative colitis).
3. Within the 4 weeks prior to administration of study drug, any disease(s) that the investigator deemed relevant to the subjects' eligibility.
4. Febrile illness within the 4 weeks before first study drug administration.
5. Known hypersensitivity to the study drugs or components of the preparations (e.g. galactose intolerance for methotrexate).
6. Known severe allergies, non-allergic drug reactions, or multiple drug allergies.
7. Known cardiovascular disorders, e.g. presence or history of prodromes of a thrombosis, valvular heart disease, atrial fibrillation or cardiac dysfunction.
8. Known or suspected malignant tumors (including any history of malignant tumors), known or suspected benign tumors of the liver and pituitary (including after treatment).
9. Known or suspected lung disease, e.g. asthma, chronic obstructive pulmonary disease, bronchiectasis, or cystic fibrosis.
10. Known or suspected immunodeficiency, e.g. history of recurrent and / or severe infections (e.g. bacterial arthritis, cellulitis, chronic sinusitis, furunculosis, meningitis, organ abscesses, osteomyelitis, otitis, pneumonia, or sepsis).
11. Liver diseases: existing or previous, acute or chronic progressive liver diseases, e.g., disturbances of bilirubin excretion (Dubin-Johnson and Rotor syndromes), cholecystectomy; disturbances of the bile secretion and the flow (cholestasis); liver fibrosis; liver dysfunction; presence or history of liver tumors (benign or malignant); history of viral hepatitis B or C. [Note: A history of hepatitis A was allowed, provided that this was fully resolved at the time of screening.]
12. Relevant kidney diseases (e.g. glomerulonephritis, renal insufficiency), or renal injury associated with multisystem diseases/disorders (e.g. systemic lupus erythematosus, diabetic nephropathy).

[Note: History of a single episode of uncomplicated nephrolithiasis did not prevent participation.]

13. Known metabolic disorders (e.g. diabetes mellitus, severe hypertriglyceridemia) that were clinically significant as judged by the investigator.
14. Known presence or any known history of psychiatric disorders, including depression that was clinically significant as judged by the investigator.
15. Known current thyroid disorders which require treatment (subjects with a euthyroid struma not requiring any treatment could participate).

Medication, drug use and special behavior patterns:

16. Regular use of therapeutic or recreational drugs, e.g. carnitine products, anabolics, high-dose vitamins.
17. Within the 4 weeks before the first study drug administration, use of systemic or topical medicines or substances that oppose the study objectives or which could have influenced their attainment (e.g. an investigational drug, or any drug known to induce liver enzymes, such as dexamethasone, barbiturates, rifampicin, anticonvulsants, griseofulvin, or St. John's wort (*Hypericum perforatum*)).
18. Within the week before first study administration, use of medicines which are sensitive substrates for the efflux transporter BCRP (e.g. atorvastatin, fluvastatin, imatinib, irinotecan, lapatinib, mitoxantrone, rosuvastatin, simvastatin, sulfasalazine, topotecan).
19. During the week before first study drug administration, repeated use of drugs that could have affected absorption (e.g. laxatives, loperamide, metoclopramide, antacids, H2-receptor antagonists).
20. During the 4 weeks before first study drug administration, use of drugs that could have affected the immune system (e.g. glucocorticoids, interleukin-1-receptor antagonists, TNFα inhibitors, live vaccines).
21. (Applies to Part B only:) Intake of non-steroidal anti-inflammatory drugs (NSAIDs), salicylates, probenecid, penicillin and proton-pump inhibitors (e.g. omeprazole, pantoprazole) and co-trimoxazole or trimethoprim 72 hours before first study drug administration.
22. Regular daily consumption of more than 800 mL of normal-strength beer or the equivalent quantity of approximately 40 g of alcohol in any other form.
23. Intake of alcohol within the 72 hours before first study drug administration.
24. Smoking within the 3 months before the screening visit.
25. Special diets preventing the subjects from eating the standard meals during the study, e.g. vegetarian or low-calorie diet.
26. Regular daily consumption of more than 1 L of xanthine-containing beverages, e.g. coffee, tea, cocoa.
27. Within the 7 days before first study drug administration, intake of foods or beverages containing furanocoumarin derivative (e.g. grapefruit, pomelo, limes, Seville orange).
28. Donation of more than 500 mL blood, or any plasmapheresis, in the four weeks before signing the informed consent form or any plans for these from the moment of signature until four weeks after the follow-up visit.
29. Acting as sperm donor after signing the informed consent form until 90 days after the last dose of the study drug(s).

Electrocardiogram, blood pressure, pulse rate:

30. Clinically relevant findings in the ECG such as a second- or third-degree AV block, prolongation of the QRS complex over 120 msec or clinically relevant prolongation of QTcB and/or QTcF.
31. Systolic blood pressure below 90 mmHg or above 145 mmHg (after at least 10 min in an upright sitting position).
32. Diastolic blood pressure below 45 mmHg or above 95 mmHg (after at least 10 min in an upright sitting position).
33. Pulse rate below 45 bpm or above 95 bpm (after at least 10 min in an upright sitting position).

Physical examination:

34. Clinically relevant findings in the physical examination.

Laboratory examination:

35. Positive results for hepatitis B virus surface antigen, hepatitis C virus antibodies (anti-HCV), human immune deficiency virus antibodies (anti-HIV 1+2), mycobacterium tuberculosis (positive whole-blood IFN-g test).
36. Positive result in urine drug screening.
37. Clinically relevant deviations of the screened laboratory parameters from their respective reference ranges, as follows:
    - High-sensitivity C-reactive protein (hsCRP) above 3.0 mg/L.
    - Aspartate transaminase and/or alanine transaminase and/or g-glutamine transferase above the upper limit of the normal range.
    - (Applies to Part B only:) pathologically low serum folate concentration (<5.38 ng/mL) or high homocysteine serum concentration (>15 µmol/L)
    - Estimated glomerular filtration rate below 60 ml/min.
    - Hematocrit, number of leucocytes and thrombocytes below the lower limit of the normal range.

Other:

38. Participation in another clinical study during the preceding 3 months (last treatment in previous study to first treatment in this study).
39. Exclusion periods from other studies or simultaneous participation in other clinical studies.
40. Scheduled (elective) surgery, planned hospitalization, planned or actual dental treatment, or planned or actual immunization that took place, or was planned to take place, between signature of the informed consent form and the end of the study last visit of the individual subject.
41. Inability or unwillingness on the subject's part to comply with the study restrictions.
42. (Applies to Part A only:) Inability or unwillingness on the subject's part to eat the complete high-fat, high-calorie meal.
43. The subject was in custody by order of an authority or a court of law.
44. Criteria which in the opinion of the investigator precluded participation for scientific reasons, for reasons of compliance, or for reasons of the subject’s safety.
45. Previous assignment to treatment (e.g. randomization) during this study (because allowing previously randomized subjects to be re-included into the study could have led to bias).
46. Close affiliation with the investigator (e.g. a close relative) or persons working at the study site.
47. The subject is an employee of Bayer AG or the CRO conducting the study.

1.3 Study procedures

1.3.1 FE/abs.BA study: Composition of meals served before drug administration

**High-fat, high-calorie meal (American breakfast)**<br><br>(~1044 kcal in total;<br>544 kcal fat, 345 kcal carbohydrates)	2 fried eggs (in 15 g butter/margarine) (~100 g)<br>1 portion of bacon (40 g)<br>1 portion of fried potatoes (115 g)<br>2 slices of (toasted) (wheat) bread with 15 g margarine<br><br>1 glass of whole milk (240 mL)
**Moderate-fat, moderate-calorie meal (Continental breakfast)**<br><br>(~564 kcal in total;<br>127 kcal fat, 363 kcal carbohydrates)	3 slices of wheat toast (3 x 40 g) with<br>half-fat margarine (20 g),<br>jam (25 g) and<br>cheese (20 g)<br><br>1 cup of decaffeinated coffee (120 mL)

The participants were instructed to eat the meal within 30 minutes or less. Thirty minutes after the start of the meal, the study drug was administered.

1.3.2 Sampling schedules

Study	Analyte	Matrix	Sampling times
**SAD study**	Zabedosertib	Plasma	Pre dose and 0.5, 1.0, 1.5, 2.0, 2.5, 3.0, 3.5, 4.0, 5.0, 6.0, 8.0, 12, 24, 36, and 48 hours and 3, 5, 7, 9, 11, and 14 days after administration of the study drug #
	Zabedosertib	Urine	Over 24 h postdose (dose groups 1-6)
	Protein binding	Plasma	Pre dose and 3, 24, and 72 hours post dose
	PD biomarkers		Pre dose, 6, 24, and 48 hours and 6 days post dose
**MAD study**	Zabedosertib	Plasma	Multiple-dose period:<br>Day 1 to 9: daily before and 4 h post dose<br>Day 10 and following: pre dose and 0.5, 1, 1.5, 2, 3, 4, 5, 6, 8, 10, 12, 15, and 24 h and 2, 3, 4, 5, and 6 d after the last dose (Day 10)
	Zabedosertib	Plasma	Single-dose period:<br>Pre dose, 0.5, 1, 2, 3, 4, 5, 6, 8, 10, 12, and 24 h and 2, 3, 4, 5, 7, and 20±2 d post dose
	PD biomarkers		Baseline, 6 h post dose on Day 1 and Day 10 of the multiple-dose period
**FE/abs.BA study**	Zabedosertib	Plasma	Pre dose and 0.5, 1.0, 1.5, 2.0, 2.5, 3.0*, 3.13, 3.25*, 3.38, 3.5, 3.75, 4.0, 4.5, 5, 6, 8, 11, 12, 15, 24, 36 and 48 hours and 3, 5, and 7 days after study drug intake in the fasted state and 0.5, 1.0, 1.5, 2.0, 2.5, 3, 4, 5, 6, 8, 12, 24, 36, and 48 hours and 3, 5, and 7 days after intake in the fed state. (Asterisks indicate the samples taken at the start and end of the IV infusion)

In case of split dosing, PK blood samples were taken pre dose, 0.5, and 1, 2, 3, 4, 5, and 12 hours after the first partial dose (evening dose). After administration of the second partial dose (morning dose), the above-described sampling schedule applied.

Abbreviations: FE/abs.BA, food effect & absolute bioavailability; IV, intravenous; MAD, multiple ascending dose; PD, pharmacodynamic; SAD, single ascending dose.

1.4 Bioanalytical methods

1.4.1 Determination of zabedosertib in plasma

Zabedosertib and [$^{13}C_6$]-zabedosertib were quantitatively determined in plasma after protein precipitation with acetonitrile/ammonium acetate buffer (SAD, MAD study) or methanol (FE/abs.BA study) containing the internal standard followed by separation employing high-pressure liquid chromatography (HPLC) and tandem mass spectrometric detection (LC-MS/MS). [$^{2}H_6$]zabedosertib was used as the internal standard. For performance parameters of the method, see Table S 1.

All samples were stored at or below -15°C and analyzed within 330 days (SAD study), 8 months (MAD study) or 149 days (FE/abs.BA study) after sampling. The stability data indicated that the analytes were stable for this time period (Table S 2). The validation of the methods and the analyses of the study samples were performed in compliance with the pertinent FDA and EMA guidelines on bioanalytical method validation.

Table S 1: Determination of zabedosertib in plasma: performance of the bioanalytical method

Calibration standards	SAD study Zabedosertib in plasma	MAD study Zabedosertib in plasma	FE/abs.BA study Zabedosertib plasma	FE/abs.BA study [$^{13}C_6$]-zabedosertib in plasma
Calibration range (LLOQ to ULOQ) [µg/L]	1.0 to 1000	10.0 to 10000	10.0 to 10 000	0.01 to 10.0
Mean inter-assay accuracy of back-calculated concentrations (except LLOQ)	#	95.1% to 103%	94.8% - 104%	97.6% to 104%
Precision (except LLOQ)	#	≤5.5%	≤5.0%	≤ 7.0%
Accuracy at the LLOQ	#	98.5%	97.1%	100%
Precision at the LLOQ	#	2.9%	2.3%,	5.1%
**Quality control samples**				
Concentration range [µg/L]	3.00 to 40000	30.0 to 40000	30.0 to 40000	0.03 to 400
Accuracy	96.4% to 100%	96.0% to 103%	96.9% to 102%	93.8% to 104%
Precision	1.8% to 4.1%.	2.6% to 7.1%	2.9% to 4.9%	3.5% to 9.8%

<u>Abbreviations</u>: LLOQ, lower limit of quantitation; ULOQ, upper limit of quantitation

Table S 2: Stability tests

Study	Bioanal. Method	Analyte	LLOQ [µg/L]	Long-term stability [days]	Short-term stability [hours]	Freeze/thaw stability	Autosampler stability [days]
SAD	A	Zabedosertib	1.00	330	96	3 cycles	4
MAD	B	Zabedosertib	10.0	270	24	3 cycles	8
FE/abs.BA	C	Zabedosertib	10.0	253	24	3 cycles	6
18387	C	[$^{13}C_6$] Zabedosertib	0.0100	149	24	3 cycles	6

References:

U.S. Department of Health and Human Services, Food and Drug Administration, Center for Drug Evaluation and Research (CDER), Center for Veterinary Medicine (CVM). Guidance for Industry: Bioanalytical Method Validation. 2001.

U.S. Department of Health and Human Services Food and Drug Administration Center for Drug Evaluation and Research (CDER) Center for Veterinary Medicine (CVM): Bioanalytical Method Validation Guidance for Industry 2018.

European Medicines Agency, Committee for Medicinal Products for Human Use (CHMP). Guideline on Bioanalytical Method Validation. 2011.

European Medicines Agency: Reflection paper for laboratories that perform the analysis or evaluation of clinical trial samples. 2012.

1.4.2 Determination of unbound fraction of zabedosertib

The unbound fraction (f_u) of zabedosertib was determined using equilibrium dialysis after spiking plasma samples *ex vivo* with radioactively labeled [^{14}C]zabedosertib at a nominal concentration of 100 µg/L. (It has been demonstrated that the f_u showed no increase when increasing the test concentrations from 100 to 500 µg/L. Therefore, the additional 100 µg/L of radiolabeled zabedosertib that was added to the study samples is considered to have only a minor influence on the determined fraction unbound.)

The unbound plasma concentrations (C_u) of zabedosertib in individual plasma samples were estimated using the following formula:

$$(1) \qquad C_{u,e} \approx C \times f_{u,e}$$

$$(2) \qquad f_{u,e} \approx -\frac{\frac{K_{D,AGP}}{C}+f_{n,HSA}\left(\frac{P_{AGP}}{C}-1\right)}{2}+\sqrt{\left\{\frac{\frac{K_{D,AGP}}{C}+f_{n,HSA}\left(\frac{P_{AGP}}{C}-1\right)}{2}\right\}^2+f_{n,HSA}\frac{K_{D,AGP}}{C}}$$

$$(3) \qquad f_{n,HSA}=\frac{1}{\left(\frac{P_{HSA}}{K_{D,HSA}}+1\right)} \quad - \text{ fraction not bound to HSA}$$

$C_{u,e}$	estimated unbound plasma concentration of BAY 1834845
C	total (bound and unbound) plasma concentration of BAY 1834845
$f_{u,e}$	estimated unbound fraction of BAY 1834845 in plasma
$f_{n,HSA}$	fraction of BAY 1834845 in plasma not bound to HSA (($C_{u,e}$ + C_{AGP})/C), where C_{AGP} is the plasma concentration of BAY 1834845 bound to AGP
P_{AGP}	Alpha-1-Acid Glycoprotein (AGP) concentration (g/L) in plasma determined at baseline

The obtained estimated unbound plasma concentrations were used to calculate unbound PK parameters.

1.4.3 Investigation of explorative pharmacodynamic biomarkers

Peripheral blood samples were drawn directly into TruCulture tubes (Myriad RBM, Austin, US) according to the manufacturer's instructions.

Blood samples were incubated in duplicate for each challenge agent at 37°C. Two incubation modes were used:

1. 6-hour incubation protocol (customized TruCulture tubes containing 0.1 ng/mL LPS) (this mode was used for TNFα in the SAD and the MAD study) and
2. 24-hour incubation protocol (standard TruCulture tubes containing 100 ng/mL LPS) (used for measurement of TNFα, IL-1β, IFNγ and IL-6 in the SAD study).

After incubation, TNFα release in culture supernatants was evaluated using multiplex immunoassays.

1.5 PopPK analysis

A population-pharmacokinetic (popPK) analysis based on PK data[1] from the SAD and the MAD study was conducted to describe the variability in the pharmacokinetics of zabedosertib taken as tablets. PK data from 77 study participants with a total of 2500 concentration measurements were included in the analysis (Table S 3).

Different compartmental population PK models were fitted to the data and the model that best described the data was selected on the basis of goodness of fit criteria and visual predictive checks. The development of the popPK model started with modeling the single-dose data. Multiple-dose data followed, and the single-dose model was adapted where needed. No covariate analysis was done.

The analysis was conducted via nonlinear mixed-effects modeling using NONMEM (ICON Development Solutions, version 7.3) with the Navigator workbench (Mango solutions,) on a Red Hat Enterprise Linux 6.3 environment. Visual predictive checks and additional evaluations were performed in R (version 3.2.5 (2016-04-14) in a validated RStudio Server Pro environment (Rstudio 1.1.423). The RxODE package, version 0.8.0-8, was used for the (visual) predictive checks (https://CRAN.R-project.org/src/contrib/Archive/RxODE/).

Table S 3: Population PK analysis: data base

Study	N of valid participants	N of valid PK samples	Mean N of valid PK samples per participant	Minimum N of valid PK samples per participant	Maximum N of valid PK samples per participant
SAD	45	1065	23.7	16	49
MAD	32	1435	44.8	30	52
Total	77	2500	32.5	16	52

Note: Zabedosertib concentrations had to be above the lower limit of quantitation to be valid for analysis

The observed nonlinearity in the PK after a single dose, which resulted in a less than proportional increase in exposure with increasing dose could be adequately described by a one-compartmental model with first-order elimination and the assumption that the relative bioavailability decreases with increasing dose.

As after multiple dosing a lower accumulation ratio was observed as expected based on the terminal half-life after a single dose, the model was adapted to capture this nonlinearity with respect to time.

Zabedosertib binds to both human serum albumin (HSA) and alpha-acid glycoprotein (AGP) *in vitro* with binding affinities of 178 and 0.4 µM respectively. This led to the hypothesis that capacity-limited binding to these proteins could change the unbound concentration over time, resulting in a change in both the total clearance and the total volume of distribution. As the affinity of Zabedosertib for AGP is considerably higher and its concentration in plasma is typically lower (~15 µM) compared to HSA (~600 µM), AGP binding is expected to have a

[1] Data obtained after administration of the liquid formulation or in the fed state in the SAD study were not included in the analysis. The analysis was based on preliminary zabedosertib concentration data. Later it was confirmed that the data were in line with the final data after data base lock.

larger influence on the unbound concentration and the modeling focused on capacity binding to AGP.

Capacity-limited binding to a binding partner in the central compartment was added to the initial single dose popPK model, which resulted in the model shown in Figure S 1.

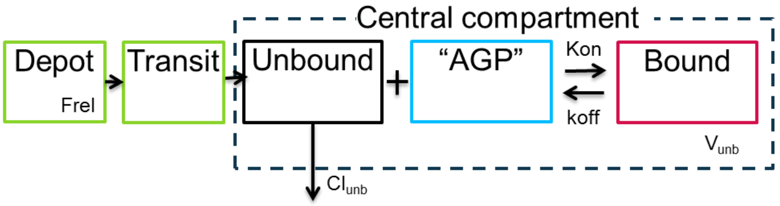

Figure S 1: Schematic representation of the popPK model with capacity-limited binding in the central compartment

It was assumed that the binding affinity (K_d) is equal to the *in vitro* binding affinity for AGP. The first-order dissociation rate constant (k_{off}) and the binding capacity ("AGP"), i.e., the concentration of the binding partner, were estimated. The association rate constant was estimated by dividing the k_{off} by the Kd. The dose-dependent decrease in the relative bioavailability (F_{rel}) was described by the equation: F1 = (f_{min} + (1 - f_{min}) * exp(-log(2)/d50 * DOSE)), in which f_{min} is lowest value for Frel at an infinitely high dose (DOSE) and the d50 is the dose at which the maximal F_{rel} value, which was fixed to 1 in this case, is reduced by 50%. Inter-individual variability was estimated for the apparent unbound clearance (Cl_{unb}), unbound volume of distribution (V_{unb}), the first-order absorption rate constant (ka) and the concentration of the binding partner in the plasma AGP. The parameter estimates of model are shown in Table S 4.

Table S 4: Parameter estimates

Parameter	Unit	Estimate	RSE [%]	LLCI	ULCI	Description
			Fixed effects (THETA)			
Cl_{unb}	L/h	15.4	12.3	11.7	19.1	Unbound clearance
V_{unb}	L	7.84	8.36	6.56	9.12	Unbound volume of distribution
k_a	h^{-1}	1.2	9.17	0.984	1.42	Absorption rate constant
K_d	µM	0.4	fixed			Binding affinity for “AGP”, fixed to *in vitro* AGP-binding affinity
k_{off}	h^{-1}	0.058	8.71	0.048	0.068	Dissociation rate constant
d50	mg	204	11.7	157	251	Dose resulting I 50% reduction in the relative bioavailability (Frel)
f_{min}		0.01	fixed			lowest value of the relative bioavailability at an infinitely high dose
“AGP”	µM	14.9	8.00	12.6	17.2	Concentration of binding partner in plasma
			Random effects: Inter-individual variability (OMEGA)			
Cl_{unb} (ω^2)	-	0.293	25.1	0.149	0.437	Inter-individual variability on Cl_{unb}
“AGP” (ω^2)	-	0.124	29.5	0.052	0.196	Inter-individual variability on “AGP”
V_{unb} (ω^2)		0.071	38.5	0.018	0.125	Inter-individual variability on V_{unb}
ka (ω^2)		0.39	15.9	0.269	0.511	Inter-individual variability on ka
			Residual error (SIGMA)			
σ^2_{prop}	-	0.037	10.7	0.029	0.045	Proportional residual error
$\sigma^2_{add,SAD}$	-	0.25	Fixed			Additive residual error for the SAD study
$\sigma^2_{add,MAD}$	-	2.5	Fixed			Additive residual error for the MAD study

Overall, the observed trend in the zabedosertib PK after both single and multiple dosing could be adequately described by the model, as can be observed in the visual predictive checks (VPC) shown in Figure S 2 and Figure S 3. On the other hand, the variability in the PK appears to be over-estimated as most observations fall within the prediction interval for 90% of the population.

The estimated first-order dissociation rate has a half-life of approximately 12 hours (Table S 4) and the binding capacity of 14.9 µM (Table S 4) is estimated to be very close to the median AGP concentration in both studies (15.2 µM).

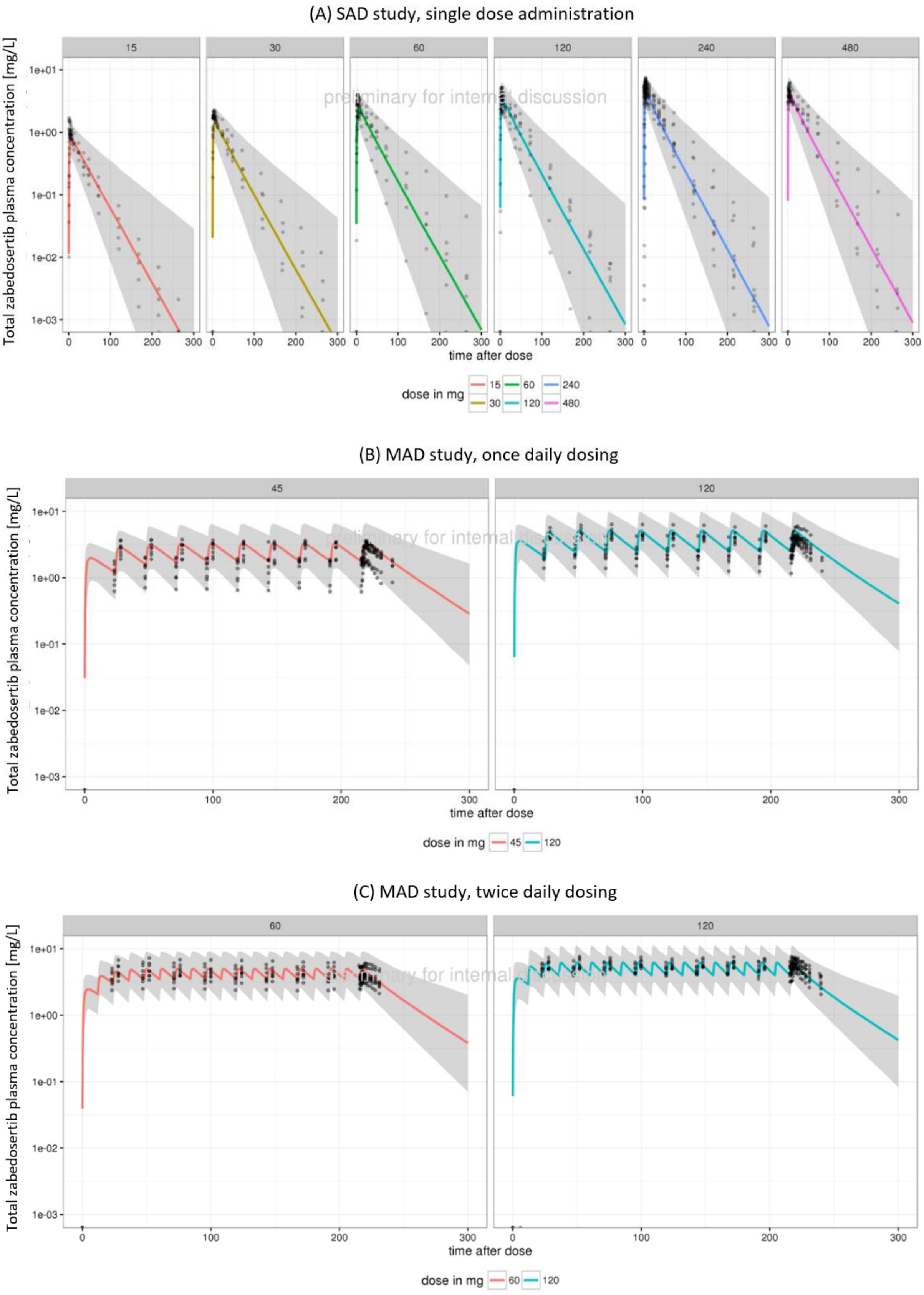

Figure S 2: Visual predictive check for (A) the SAD study, (B) the MAD study after once daily dosing and (C) the MAD study after twice daily dosing

The shaded area shows the predicted variability for 90% of the population. The colored lines show the predictions for a typical participant and the symbols show the observations.

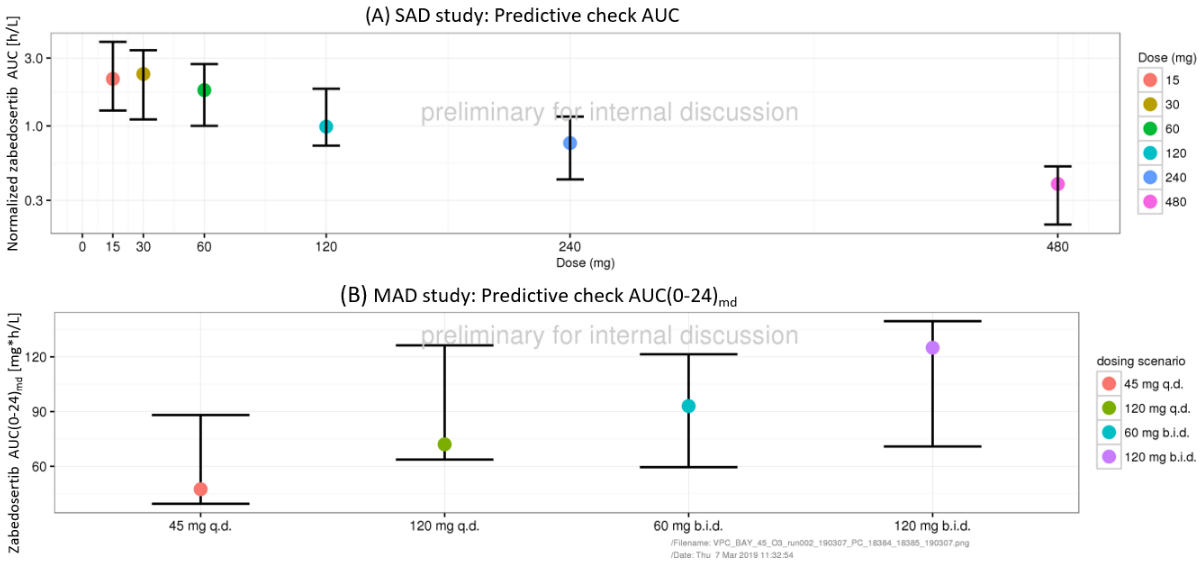

Figure S 3: Predictive check of (A) the observed dose-normalized AUC[0-inf] after single dose in the SAD study after single dose, (B) the observed AUC[0-24] on day after once or twice daily administration.

Note: The error bars show the 95% confidence limits of the predicted geometric mean of the AUC based on bootstrap resampling with replacement of a cohort with n=5 or 6 participants from n=1000 simulated AUC values per treatment cohort. The colored symbols show observed geometric mean AUC values for each cohort.

The estimated first-order dissociation rate has a half-life of approximately 12 hours, which is relatively long compared to *in vitro* estimates of plasma protein binding with dissociation half-lives of seconds (Zheng et al. 2014). The binding capacity of 14.9 µM is estimated to be very close to the median AGP concentration in both studies (15.2 µM). Also, the individual estimates of the binding partner concentrations show a weak positive correlation (Pearson correlation coefficient of 0.28) with the observed AGP concentrations (Figure S 4). However, it should be noted that the binding to AGP is a model assumption and must not necessarily reflect actual biology. Therefore inferences from the model, e.g. higher drug exposure with higher AGP concentrations, should be drawn cautiously.

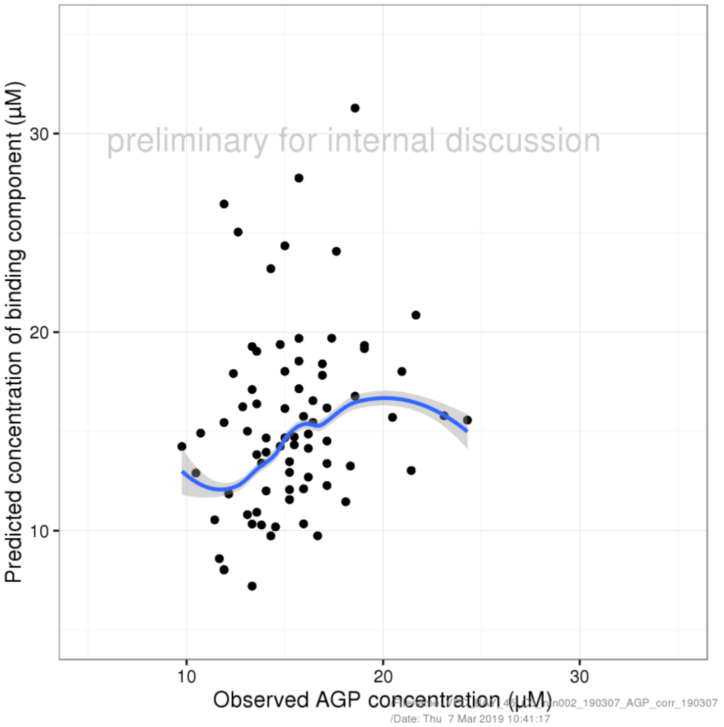

Figure S 4: Correlation between individual estimates of the "AGP" concentration and the observed AGP concentration in the SAD study and the MAD study

The symbols show the individual data. The solid blue line shows a loess regression fit of the data. The gray area shows the uncertainty of the loess fit.

References

Zheng et. al. (2014) Determination of Rate Constants and Equilibrium Constants for Solution-Phase Drug−Protein Interactions by Ultrafast Affinity Extraction. Anal. Chem., 86, 6454−6460

1.6 Target occupancy – IC_{50} for IL-6 inhibition

As described in the Manuscript, Section 2.11, it was assumed that the target occupancy of zabedosertib can be inferred from the IC_{50} for IL-6 inhibition after resiquimod (R848) stimulation in an *in vitro* whole-blood assay.

For this assay, blood samples were obtained from healthy donors via venipuncture using Li-heparin tubes. The blood was incubated with a range of zabedosertib concentrations for 1 hour at 37°C, and 1 mL of the treated blood was stimulated in a TruCulture Tube (Myriad RBM) pre-filled with 2 mL of medium containing resiquimod, resulting in a final concentration of 1 µM of resiquimod in the sample. The blood was incubated for 6 hours at 37°C and the supernatant was harvested via TruCulture filtration. Analysis of IL-6 levels in the stimulated TruCulture supernatants was performed using the V-PLEX Human Proinflammatory Panel II (Meso Scale Discovery, Rockville, Maryland), in accordance with the manufacturer’s instructions.

2 Supplemental Material: Study Participants

2.1 SAD study: Participant demographics and other baseline characteristics

	5 mg zabedo. liquid	15 mg zabedo. liquid & tablet [#]	30 mg zabedo.	60 mg zabedo.	120 mg zabedo.	240 mg zabedo.	240 mg zabedo. [$] fed	480 mg zabedo.	480 mg zabedo. split fed	Placebo 5 mg liquid	Placebo 15 mg liquid & tablet [#]	Placebo 30-480 mg	Placebo 240 mg fed [$]	Placebo 480 mg split fed	Total
	N=6	N=6	N=5	N=6	N=6	N=5	N=6	N=6	N=6	N=2	N=2	N=10	N=2	N=2	N=70
**Sex** = male	6 (100%)	6 (100%)	5 (100%)	6 (100%)	6 (100%)	5 (100%)	6 (100%)	6 (100%)	6 (100%)	2 (100%)	2 (100%)	10 (100%)	2 (100%)	2 (100%)	70 (100%)
**Race**															
White	6 (100%)	6 (100%)	5 (100%)	5 (83%)	6 (100%)	5 (100%)	6 (100%)	6 (100%)	5 (83%)	2 (100%)	2 (100%)	9 (90%)	2 (100%)	2 (100%)	67 (96%)
Asian	-	-	-	-	-	-	-	-	-	-	-	1 (10%)	-	-	1 (1%)
Black/African American	--	-	-	1 (17%)	-	-	-	-	-	-	-	-	-	-	1 (1%)
Native Hawaiin	--	-	-	-	-	-	-	-	1 (17%)	-	-	-	-	-	1 (1%)
**Ethnicity**															
Hispanic/Latino	-	1 (17%)	-	-	-	-	-	-	-	-	-	-	-	-	1 (1%)
Non-Hispanic/ Latino	6 (100%)	5 (83%)	5 (100%)	6 (100%)	6 (100%)	5 (100%)	6 (100%)	6 (100%)	6 (100%)	2 (100%)	2 (100%)	10 (100%)	2 (100%)	2 (100%)	69 (99%)
**Age** (years)															
Mean ± SD	37.0±9.1	37.5±4.9	39.8±6.8	34.2±9.3	38.5±11.3	40.6±3.7	40.5±7.6	33.7±10.3	36.7±10.2	37.5±4.9	38.0±11.3	39.8±7.3	43.0±1.4	45.5±6.4	38.3±8.1
Range	27-48	27-49	34-49	22-50	26-50	38-47	28-48	21-46	21-46	34-41	30-46	24-49	42-44	41-50	21-50
**Weight (kg)**															
Mean ± SD	81.5±9.08	83.8±9.35	82.1±10.8	73.5±7.67	82.8±6.16	80.8±8.05	85.1±15.6	77.1±8.03	79.6±7.54	90.0±22.1	82.6±1.06	82.4±8.95	79.3±8.49	82.0±11.5	81.2±9.41
Range	69.5-91.8	70.3-98.3	69.1-97.0	62.0-82.0	76.2-93.3	69.5-90.7	61.3-98.6	67.1-85.5	70.0-90.3	74.3-106	81.8-83.3	67.5-94.5	73.3-85.3	73.8-90.1	61.3-106
**Height (cm)**															
Mean ± SD	184±5.3	183±5.4	179±7.1	177±8.1	179±6.1	180±6.8	178±7.0	181±4.4	176±8.2	185±17.7	184±12.0	184±9.6	180±3.5	178±2.8	180±7.4
Range	179-191	176-192	170-186	166-188	172-187	173-188	167-188	174-186	170-188	172-197	175-192	170-197	177-182	176-180	166-197
**BMI** (kg/m^2)															
Mean ± SD	24.0±2.48	25.2±2.87	25.6±1.65	23.7±2.87	26.0±2.85	24.9±1.41	26.8±3.55	23.6±1.87	25.8±1.52	26.2±1.48	24.7±2.90	24.4±2.20	24.6±1.70	26.0±4.45	25.0±2.44
Range	19.9-27.1	21.0-29.0	23.9-28.3	20.0-28.1	21.8-30.1	23.2-26.8	22.0- 29.7	21.4- 26.1	24.2-28.0	25.1-27.2	22.6- 26.7	21.5-28.9	23.4- 25.8	22.8- 29.1	19.9-30.1

Zabedosertib was administered in fasted state unless indicated otherwise. Self-reported ethnicity and race classification was routinely documented. For classification criteria, see the 2026 FDA guidance on *Collection of Race and Ethnicity Data in Clinical Trials* (FDA-2016-D-3561). [#] as liquid formulation and as tablet; [$] 2x120 mg split and as one dose. Abbreviations: zabedo., zabedosertib; BMI, body mass index; SD, standard deviation.

2.2 MAD study: Participant demographics and other baseline characteristics

	Placebo N=10 (100%)	45 mg zabedosertib QD N=8 (100%)	120 mg zabedosertib QD N=8 (100%)	60 mg zabedosertib BID N=8 (100%)	120 mg zabedosertib BID N=8 (100%)	200 mg zabedosertib BID N=8 (100%)	Total N=50 (100%)
**Sex**							
Male	10 (100%)	8 (100%)	8 (100%)	8 (100%)	8 (100%)	8 (100%)	50 (100%)
**Race**							
White	10 (100%)	8 (100%)	8 (100%)	8 (100%)	8 (100%)	8 (100%)	50 (100%)
**Ethnicity**							
Hispanic or Latino	1 (10.0%)	0	0	0	0	0	1 (2.0%)
Not Hispanic or Latino	9 (90.0%)	8 (100%)	8 (100%)	8 (100%)	8 (100%)	8 (100%)	49 (98.0%)
**Age** (years)							
Mean ± SD	34.9±10.7	39.5±6.1	41.3±8.9	31.0±7.4	30.9±7.4	37.8±8.4	35.8±8.9
Range	22-50	33-47	25-50	20-41	21-41	20-48	20-50
**Weight** (kg)							
Mean ± SD	79.9±14.0	90.3±9.90	83.8±7.20	91.3±7.9	74.6±10.4	86.0±12.3	84.2±11.7
Range	65.9-111	75.9-99.1	75.4-96.9	76-102	62.8-97.1	66.8-101	62.8-111
**Height** (cm)							
Mean ± SD	180±8.39	185±7.04	182±4.66	186±7.80	177±5.87	182±9.36	182±7.57
Range	170-195	172-195	173-187	177-200	165-183	166-197	165-200
**Body mass index** (kg/m^2)							
Mean ± SD	24.5±2.52	26.4±2.34	25.2±1.72	26.6±2.73	23.8±3.12	25.8±2.11	25.3±2.55
Range	21.6-29.3	23.3-29.2	23.4-28.6	22.0-29.7	19.4-29.0	23.6-28.8	19.4-29.7

Abbreviations: BID, twice daily; N, number of participants; SD, standard deviation; QD, once daily

2.3 FE&BA study: Participant demographics and other baseline characteristics

	**Zabedosertib 120 mg HF - FS – MF** [#] N=5 (100%)	**Zabedosertib 120 mg FS - HF - MF** [#] N=5 (100%)	**Total** N=10 (100%)
**Sex**			
Male	5 (100%)	5 (100%)	10 (100%)
**Race**			
White	5 (100%)	5 (100%)	10 (100%)
**Ethnicity**			
Not Hispanic or Latino	5 (100%)	5 (100%)	10 (100%)
**Age** (years)			
Mean ± SD	28.8±11.1	27.4±3.8	28.1±7.9
Range	18-43	25-34	18-43
**Weight** (kg)			
Mean ± SD	79.0±13.8	84.0±11.1	81.5±12.1
Range	68.2-103	77.4-104	68.2-104
**Height** (cm)			
Mean ± SD	184±8.96	182±7.40	183±7.80
Range	173-194	173-190	173-194
**Body mass index** (kg/m2)			
Mean ± SD	23.4±3.49	25.4±2.34	24.4±2.99
Range	19.8-28.6	22.3-28.7	19.8-28.7

[#] treatment sequence (intake in fasted state in one period and following a high-fat, high-calorie or moderate-fat, moderate-calorie meal in the other two periods). Abbreviations: FS, drug intake in fasted state; HF, drug intake after a high-fat, high-calorie meal; MF, drug intake after a moderate-fat, moderate-calorie meal; SD, standard deviation;

2.4 SAD study: Participant disposition

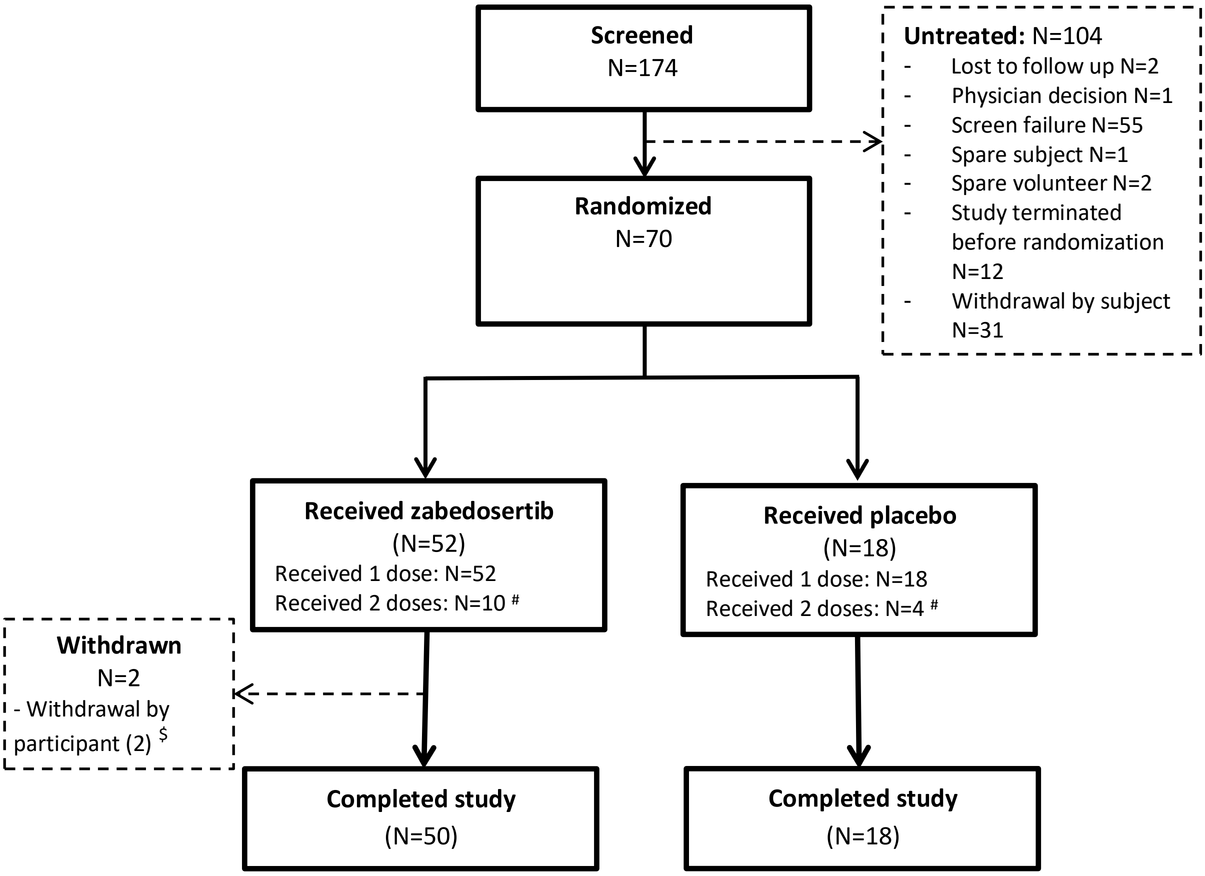

[#] Participants in the 15 mg liquid/15 mg tablet group and the 240 mg split fed/240 mg fed group received two doses of study drug. [$] Both participants were randomized to a dose group with two treatment periods (one participant to the 15 mg liquid/15 mg tablet group and one to the 240 mg split fed/240 mg fed group). They completed treatment period 1, but did not complete treatment period 2.

2.5 MAD study: Participant disposition

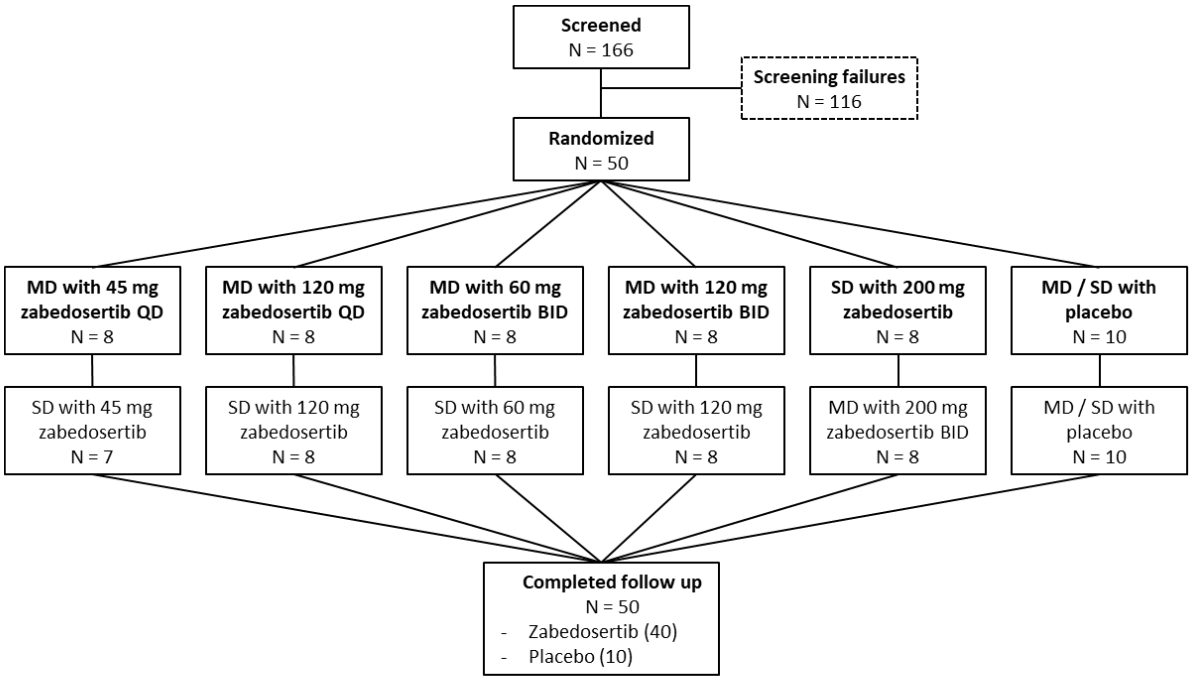

Participants who received 200 mg zabedosertib started with single-dose administration in period 1, followed by multiple-dose administration in period 2. Abbreviations: BID, twice daily; MD, multiple-dose period; QD, once daily; SD, single-dose period.

2.6 FE/abs.BA study: Participant disposition

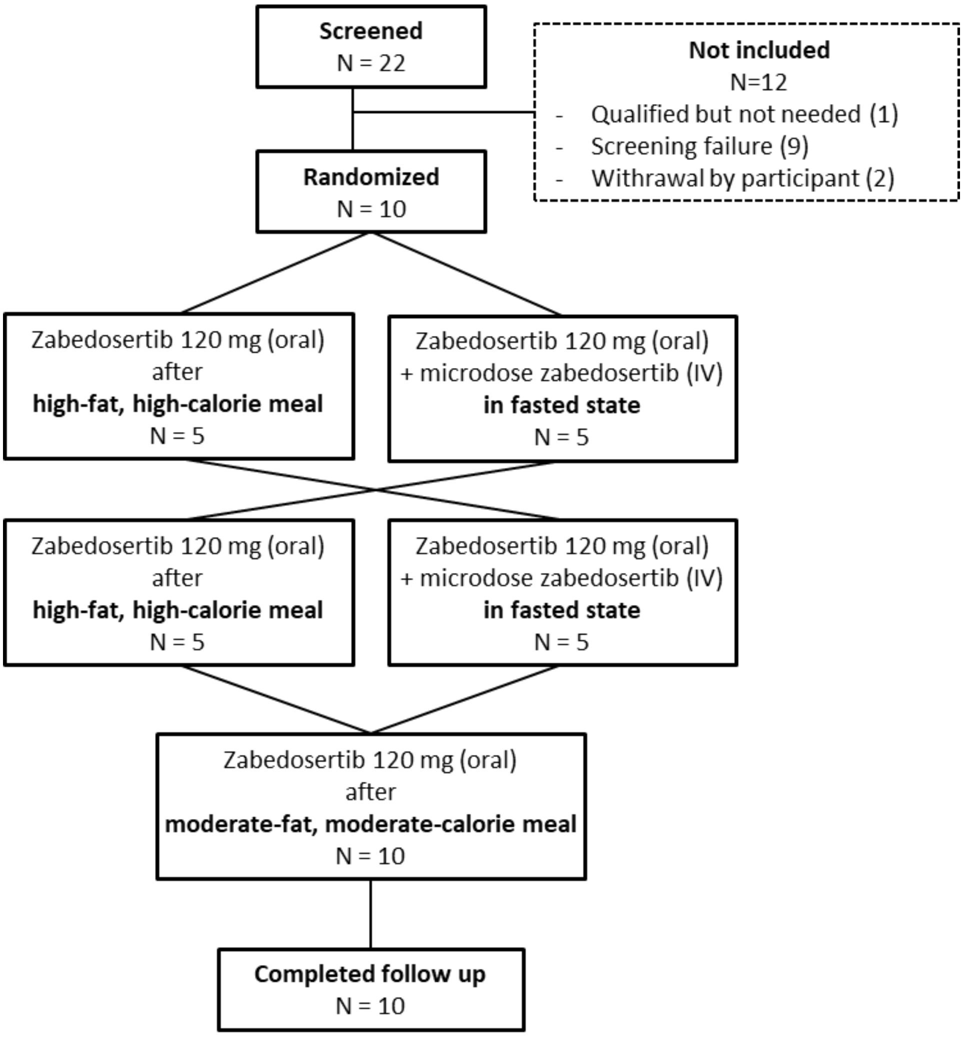

Abbreviations: IV, intravenous.

3 Supplemental Material: Safety

3.1 SAD study: Overall summary of treatment-emergent adverse events

	5 mg zabedo. liquid N=6 (100%)	15 mg zabedo. liquid N=6 (100%)	15 mg zabedo. N=5 (100%)	30 mg zabedo. N=5 (100%)	60 mg zabedo. N=6 (100%)	120 mg zabedo. N=6 (100%)	240 mg zabedo. N=5 (100%)	240 mg zabedo. split, fed N=6 (100%)	240 mg zabedo. fed N=5 (100%)	480 mg zabedo. N=6 (100%)	480 mg zabedo. split, fed N=6 (100%)	Placebo 5/15 liquid N=4 (100%)	Placebo 30/60/120/ 240/ 480 N=12 (100%)	Placebo 240 fed N=2 (100%)	Placebo 240/ 480 split, fed N=4 (100%)	Total N=70 (100%)
Any TEAE	2 (33%)	3 (50%)	1 (20%)	3 (60%)	3 (50%)	2 (33%)	4 (80%)	2 (33%)	1 (20%)	3 (50%)	3 (50%)	1 (25%)	3 (25%)	0	1 (25%)	30 (43%)
Maximum intensity for any TEAE																
Mild	1 (17%)	3 (50%)	1 (20%)	3 (60%)	3 (50%)	2 (33%)	3 (60%)	2 (33%)	1 (20%)	3 (50%)	3 (50%)	1 (25%)	2 (17%)	0	1 (25%)	27 (39%)
Moderate	1 (17%)	0	0	0	0	0	1 (20%)	0	0	0	0	0	1 (8%)	0	0	3 (4%)
Any TEAE related to study drug	1 (17%)	1 (17%)	0	0	0	0	0	1 (17%)	0	0	0	0	1 (8%)	0	0	4 (6%)
Maximum intensity for any TEAE related to study drug																
Mild	0	1 (17%)	0	0	0	0	0	1 (17%)	0	0	0	0	1 (8%)	0	0	3 (4%)
Moderate	1 (17%)	0	0	0	0	0	0	0	0	0	0	0	0	0	0	1 (1%)
Any AESI	0	0	0	0	0	0	0	0	0	0	0	0	0	0	0	0
Any serious TEAE	0	0	0	0	0	0	0	0	0	0	0	0	0	0	0	0

Dark-green shading highlights 2-period data. Abbreviations: AESI, treatment-emergent adverse event of special interest; TEAE, treatment-emergent adverse event; zabedo., zabedosertib.

3.2 SAD study: Treatment-emergent adverse events by primary MedDRA system organ class and preferred term

MedDRA System organ class Preferred term [#]	5 mg zabedo. liquid N=6 (100%)	15 mg zabedo. liquid N=6 (100%)	15 mg zabedo. N=5 (100%)	30 mg zabedo. N=5 (100%)	60 mg zabedo. N=6 (100%)	120 mg zabedo. N=6 (100%)	240 mg zabedo. N=5 (100%)	240 mg zabedo. split fed N=6 (100%)	240 mg zabedo. fed N=5 (100%)	480 mg zabedo. N=6 (100%)	480 mg zabedo. split fed N=6 (100%)	Placebo 5/15 mg liquid N=4 (100%)	Placebo 30/60/ 120/ 240/ 480 mg N=12 (100%)	Placebo 240 fed N=2 (100%)	Placebo 240/ 480 split fed N=4 (100%)	Total N=70 (100%)
All events irrespective of the investigator’s assessment of the drug-event relationship																
Any TEAE	***2 (33%)***	***3 (50%)***	***1 (20%)***	***3 (60%)***	***3 (50%)***	***2 (33%)***	***4 (80%)***	***2 (33%)***	***1 (20%)***	***3 (50%)***	***3 (50%)***	***1 (25%)***	***3 (25%)***	***0***	***1 (25%)***	***30 (43%)***
Investigations	1 (17%)	2 (33%)	1 (20%)	2 (40%)	1 (17%)	0	2 (40%)	0	0	1 (17%)	1 (17%)	0	1 (8%)	0	1 (25%)	12 (17%)
Blood bilirubin increased	0	2 (33%)	1 (20%)	0	0	0	1 (20%)	0	0	0	0	0	0	0	0	3 (4%)
Blood creatine phosphokinase increased	0	0	0	0	0	0	0	0	0	0	1 (17%)	0	0	0	1 (25%)	2 (3%)
CRP increased	1 (17%)	0	0	2 (40%)	1 (17%)	0	2 (40%)	0	0	1 (17%)	0	0	1 (8%)	0	0	8 (11%)
Infections and infestations	2 (33%)	0	0	0	2 (33%)	0	2 (40%)	1 (17%)	0	1 (17%)	1 (17%)	0	1 (8%)	0	0	10 (14%)
Nasopharyngitis	1 (17%)	0	0	0	1 (17%)	0	2 (40%)	0	0	1 (17%)	1 (17%)	0	0	0	0	6 (9%)
Rhinitis	1 (17%)	0	0	0	1 (17%)	0	0	1 (17%)	0	0	0	0	0	0	0	3 (4%)
Gastrointestinal disorders	0	1 (17%)	1 (20%)	1 (20%)	0	0	0	1 (17%)	0	0	0	0	0	0	0	4 (6%)
Nausea	0	1 (17%)	0	1 (20%)	0	0	0	0	0	0	0	0	0	0	0	2 (3%)
Musculoskeletal and connective tissue disorders	0	0	0	1 (20%)	0	0	0	0	1 (20%)	1 (17%)	0	0	0	0	1 (25%)	4 (6%)
Nervous system disorders	0	0	1 (20%)	0	0	1 (17%)	0	0	0	0	0	1 (25%)	1 (8%)	0	0	4 (6%)
Headache	0	0	1 (20%)	0	0	1 (17%)	0	0	0	0	0	1 (25%)	1 (8%)	0	0	4 (6%)
General disorders and administration site conditions	0	1 (17%)	0	0	0	0	0	1 (17%)	0	0	0	0	0	0	0	2 (3%)
Injury, poisoning and procedural complications	0	0	0	0	0	1 (17%)	1 (20%)	0	0	0	0	0	0	0	0	2 (3%)
Respiratory, thoracic and mediastinal disorders	0	0	0	0	1 (17%)	0	0	0	0	1 (17%)	0	0	0	0	0	2 (3%)
Cough	0	0	0	0	1 (17%)	0	0	0	0	1 (17%)	0	0	0	0	0	2 (3%)
Skin and subcutaneous tissue disorders	0	0	0	0	0	0	0	0	0	0	1 (17%)	0	1 (8%)	0	0	2 (3%)

Continued next page

**MedDRA System organ class Preferred term #**	**5 mg zabedo. liquid**	**15 mg zabedo. liquid**	**15 mg zabedo.**	**30 mg zabedo.**	**60 mg zabedo.**	**120 mg zabedo.**	**240 mg zabedo.**	**240 mg zabedo. split fed**	**240 mg zabedo. fed**	**480 mg zabedo.**	**480 mg zabedo. split fed**	**Placebo 5/15 mg liquid**	**Placebo 30/60/ 120/ 240/ 480 mg**	**Placebo 240 fed**	**Placebo 240/ 480 split fed**	**Total**
	N=6 (100%)	N=6 (100%)	N=5 (100%)	N=5 (100%)	N=6 (100%)	N=6 (100%)	N=5 (100%)	N=6 (100%)	N=5 (100%)	N=6 (100%)	N=6 (100%)	N=4 (100%)	N=12 (100%)	N=2 (100%)	N=4 (100%)	N=70 (100%)
Ear and labyrinth disorders	0	0	0	0	0	0	0	0	0	0	1 (17%)	0	0	0	0	1 (1%)
**Events that the investigator assessed as treatment related**																
Any study-drug-related TEAE	***1 (17%)***	***1 (17%)***	***0***	***0***	***0***	***0***	***0***	***1 (17%)***	***0***	***0***	***0***	***0***	***1 (8%)***	***0***	***0***	***4 (6%)***
Gastrointestinal disorders	0	1 (17%)	0	0	0	0	0	1 (17%)	0	0	0	0	0	0	0	2 (3%)
Diarrhoea	0	0	0	0	0	0	0	1 (17%)	0	0	0	0	0	0	0	1 (1%)
Nausea	0	1 (17%)	0	0	0	0	0	0	0	0	0	0	0	0	0	1 (1%)
Infections and infestations	1 (17%)	0	0	0	0	0	0	0	0	0	0	0	0	0	0	1 (1%)
Nasopharyngitis	1 (17%)	0	0	0	0	0	0	0	0	0	0	0	0	0	0	1 (1%)
Nervous system disorders	0	0	0	0	0	0	0	0	0	0	0	0	1 (8%)	0	0	1 (1%)
Dizziness	0	0	0	0	0	0	0	0	0	0	0	0	1 (8%)	0	0	1 (1%)

TEAEs were coded using the Medical Dictionary for Regulatory Activities (MedDRA) version 20.1. All TEAEs had resolved by the end of the study except blood bilirubin increased in a participant from the 240 mg group and blood alkaline phosphatase increased in a participant from the 480 mg split-dose group. As some participants received more than one treatment, the column 'Total' is not necessarily the sum of the other columns. Dark-green shading highlights 2-period data. # Only preferred terms reported for at least 2 participants are presented here. Abbreviations: CRP, C-reactive protein.

3.3 MAD study, single-dose period: Overall summary of treatment-emergent adverse events

	Placebo N=10 (100%)	45 mg zabedosertib QD N=7 (100%) #	120 mg zabedosertib QD N=8 (100%)	60 mg zabedosertib BID N=8 (100%)	120 mg zabedosertib BID N=8 (100%)	200 mg zabedosertib BID N=8 (100%)	Total N=49 (100%) #
Any TEAE	***2 (20%)***	***1 (14%)***	***1 (13%)***	***5 (62.5%)***	***2 (25%)***	***3 (37.5%)***	***14 (29%)***
Maximum intensity for any TEAE							
mild	2 (20%)	1 (14%)	1 (13%)	3 (37.5%)	0	3 (37.5%)	10 (20%)
moderate	0	0	0	2 (25%)	1 (13%)	0	3 (6%)
severe	0	0	0	0	1 (13%)	0	1 (2%)
Any TEAE related to study drug	0	1 (14%)	0	1 (13%)	0	0	2 (4%)
Maximum intensity for study-drug-related TEAE							
mild	0	1 (14%)	0	0	0	0	1 (2%)
moderate	0	0	0	1 (13%)	0	0	1 (2%)
Any AESI	0	0	0	0	1 (13%)	0	1 (2%)
Maximum intensity for TEAE(s) of special interest							
severe	0	0	0	0	1 (13%)	0	1 (2%)
Any TEAE leading to discontinuation of study drug	0	0	0	0	0	0	0
Any serious TEAE	0	0	0	0	1 (13%)	0	1 (2%)

Two adverse events of special interest were reported: One participant in the 45 mg zabedosertib QD group reported an episode of herpes labialis, which was assessed as mild. This event of special interest was assessed by the investigator as unrelated to zabedosertib. One participant in the 120 mg zabedosertib QD reported herpes labialis, which was assessed as mild. The TEAE of special interest was judged by the investigator as related to zabedosertib. # One participant from the 45 mg group who withdrew due to an adverse event already present before single-dose administration was excluded from the safety evaluation of the single-dose period. Abbreviations: AESI, treatment-emergent adverse event of special interest; BID, twice daily; QD, once daily; TEAE, treatment-emergent adverse event.

3.4 MAD study, single-dose period: Treatment-emergent adverse events by MedDRA system organ class and preferred term

MedDRA<br>System organ class<br>Preferred term	Placebo<br>N=10 (100%)	45 mg zabedosertib QD<br>N=7 (100%) #	120 mg zabedosertib QD<br>N=8 (100%)	60 mg zabedosertib BID<br>N=8 (100%)	120 mg zabedosertib BID<br>N=8 (100%)	200 mg zabedosertib BID<br>N=8 (100%)	Total #<br>N=49 (100%)
**All events irrespective of the investigator's assessment of the drug-event relationship**							
Any TEAE	***2 (20%)***	***1 (14%)***	***1 (13%)***	***5 (63%)***	***2 (25%)***	***3 (38%)***	***14 (29%)***
Ear and labyrinth disorders	0	0	0	0	0	1 (13%)	1 (2%)
Ear discomfort	0	0	0	0	0	1 (13%)	1 (2%)
Tinnitus	0	0	0	0	0	1 (13%)	1 (2%)
Gastrointestinal disorders	1 (10%)	0	0	0	0	0	1 (2%)
Vomiting	1 (10%)	0	0	0	0	0	1 (2%)
Infections and infestations	1 (10%)	1 (14%)	0	1 (13%)	1 (13%)	0	4 (8%)
Cellulitis	0	0	0	0	1 (13%)	0	1 (2%)
Nasopharyngitis	1 (10%)	1 (14%)	0	1 (13%)	0	0	3 (6%)
Injury, poisoning and procedural complications	0	0	0	1 (13%)	1 (13%)	1 (13%)	3 (6%)
Arthropod sting	0	0	0	1 (13%)	0	0	1 (2%)
Limb injury	0	0	0	0	0	1 (13%)	1 (2%)
Scratch	0	0	0	0	1 (13%)	0	1 (2%)
Musculoskeletal and connective tissue disorders	0	0	1 (13%)	0	1 (13%)	0	2 (4%)
Joint swelling	0	0	0	0	1 (13%)	0	1 (2%)
Myalgia	0	0	1 (13%)	0	0	0	1 (2%)
Neoplasms benign, malignant and unspecified (incl cysts and polyps)	0	0	0	0	0	1 (13%)	1 (2%)
Skin papilloma	0	0	0	0	0	1 (13%)	1 (2%)
Nervous system disorders	0	0	0	2 (25%)	1 (13%)	1 (13%)	4 (8%)
Headache	0	0	0	1 (13%)	1 (13%)	1 (13%)	3 (6%)
Muscle contractions involuntary	0	0	0	1 (13%)	0	0	1 (2%)
Psychiatric disorders	1 (10%)	0	0	0	0	0	1 (2%)
Insomnia	1 (10%)	0	0	0	0	0	1 (2%)
Respiratory, thoracic and mediastinal disorders	2 (20%)	0	0	1 (13%)	0	0	3 (6%)
Cough	1 (10%)	0	0	0	0	0	1 (2%)
Oropharyngeal pain	0	0	0	1 (13%)	0	0	1 (2%)
Rhinorrhoea	1 (10%)	0	0	0	0	0	1 (2%)
**Events that the investigator assessed as treatment related**							
Nasopharyngitis	0	1 (14%)	0	1 (13%)	0	0	2 (4%)

TEAEs were coded using the Medical Dictionary for Regulatory Activities (MedDRA) version 23.0. # One participant from the 45 mg group who withdrew due to an adverse event already present before single-dose administration was excluded from the safety evaluation of the single-dose period. Abbreviations: BID, twice daily; QD, once daily; TEAE, treatment-emergent adverse event.

3.5 MAD study, multiple-dose period: Overall summary of treatment-emergent adverse events

	**Placebo** N=10 (100%)	**45 mg zabedosertib QD** N=8 (100%)	**120 mg zabedosertib QD** N=8 (100%)	**60 mg zabedosertib BID** N=8 (100%)	**120 mg zabedosertib BID** N=8 (100%)	**200 mg zabedosertib BID** N=8 (100%)	**Total** N=50 (100%)
Any TEAE	***3 (30%)***	***3 (38%)***	***4 (50%)***	***6 (75%)***	***4 (50%)***	***4 (50%)***	***24 (48%)***
Maximum intensity for any TEAE							
mild	2 (20%)	3 (38%)	4 (50%)	6 (75%)	3 (38%)	4 (50%)	22 (44%)
moderate	1 (10%)	0	0	0	1 (13%)	0	2 (4%)
Any study-drug-related TEAE	1 (10%)	0	1 (13%)	0	1 (13%)	1 (13%)	4 (8%)
Maximum intensity for study-drug-related AE							
mild	1 (10%)	0	1 (13%)	0	1 (13%)	1 (13%)	4 (8%)
Any midazolam related TEAE	0	0	0	0	0	0	0
Any AESI	0	1 (13%)	1 (13%)	0	0	0	2 (4%)
Maximum intensity for AEs of special interest							
mild	0	1 (13%)	1 (13%)	0	0	0	2 (4%)
Any TEAE leading to discontinuation of study drug	0	0	0	0	0	0	0
Any serious TEAE	0	0	0	0	0	0	0

Abbreviations: AESI, treatment-emergent adverse event of special interest; TEAE, treatment-emergent adverse event.

3.6 FE/abs.BA study: Overall summary of treatment-emergent adverse events

	**Zabedosertib 120 mg taken after a high-fat, high-calorie meal** N = 10	**Zabedosertib 120 mg taken after a moderate-fat, moderate-calorie meal** N = 10	**Zabedosertib 120 mg taken on an empty stomach** N = 10	**Total** N = 10
Any TEAE	***4 (40%)***	***5 (50%)***	***5 (50%)***	***8 (80%)***
Maximum intensity for any TEAE				
Mild	4 (40%)	5 (50%)	5 (50%)	8 (80%)
Any zabedosertib-tablet-related TEAE	1 (10%)	0	0	1 (10%)
Maximum intensity for any zabedosertib-tablet-related TEAE				
Mild	1 (10%)	0	0	1 (10%)
Any zabedosertib-IV-related TEAE	0	0	0	0
Any zabedosertib (oral or iv)-related TEAE leading to discontinuation of the study	0	0	0	0
Any AESI	0	0	0	0
Any serious TEAE	0	0	0	0

Abbreviations: AESI, treatment-emergent adverse event of special interest; TEAE, treatment-emergent adverse event.

3.7 FE/abs.BA study: Treatment-emergent adverse events

MedDRA System organ class<br>Preferred term	Zabedosertib 120 mg taken after a high-fat, high-calorie meal<br>N = 10	Zabedosertib 120 mg taken after a moderate-fat, moderate-calorie meal<br>N = 10	Zabedosertib 120 mg taken on an empty stomach<br>N = 10	Total<br>N = 10
All events irrespective of the investigator's assessment of the drug-event relationship				
Any TEAE	***4 (40%)***	***5 (50%)***	***5 (50%)***	***8 (80%)***
Eye disorders	1 (10%)	0	0	1 (10%)
Vision blurred	1 (10%)	0	0	1 (10%)
Gastrointestinal disorders	3 (30%)	2 (20%)	4 (40%)	6 (60%)
Abdominal pain	0	2 (20%)	1 (10%)	2 (20%)
Abdominal pain upper	0	0	1 (10%)	1 (10%)
Diarrhoea	0	0	1 (10%)	1 (10%)
Dyspepsia	0	0	1 (10%)	1 (10%)
Flatulence	1 (10%)	1 (10%)	2 (20%)	3 (30%)
Nausea	2 (20%)	1 (10%)	1 (10%)	3 (30%)
General disorders and administration site conditions	0	0	2 (20%)	2 (20%)
Catheter site related reaction	0	0	1 (10%)	1 (10%)
Fatigue	0	0	1 (10%)	1 (10%)
Vessel puncture site haematoma	0	0	1 (10%)	1 (10%)
Catheter site paraesthesia	0	0	1 (10%)	1 (10%)
Infections and infestations	1 (10%)	4 (40%)	0	5 (50%)
Fungal skin infection	0	1 (10%)	0	1 (10%)
Nasopharyngitis	1 (10%)	4 (40%)	0	5 (50%)
Musculoskeletal and connective tissue disorders	1 (10%)	0	0	1 (10%)
Musculoskeletal stiffness	1 (10%)	0	0	1 (10%)
Nervous system disorders	4 (40%)	1 (10%)	2 (20%)	5 (50%)
Dizziness	0	1 (10%)	1 (10%)	2 (20%)
Headache	4 (40%)	1 (10%)	1 (10%)	4 (40%)
Paraesthesia	1 (10%)	0	0	1 (10%)
Somnolence	0	1 (10%)	0	1 (10%)
Respiratory, thoracic and mediastinal disorders	1 (10%)	0	0	1 (10%)
Nasal congestion	1 (10%)	0	0	1 (10%)
Skin and subcutaneous tissue disorders	1 (10%)	1 (10%)	0	2 (20%)
Dry skin	1 (10%)	1 (10%)	0	2 (20%)

Continued next page

	Zabedosertib 120 mg taken after a high-fat, high-calorie meal	Zabedosertib 120 mg taken after a moderate-fat, moderate-calorie meal	Zabedosertib 120 mg taken on an empty stomach	Total
	Events that the investigator assessed as treatment related			
Any study-drug-related TEAE	***1 (10%)***	***0***	***0***	***1 (10%)***
Eye disorders	1 (10%)	0	0	1 (10%)
Vision blurred	1 (10%)	0	0	1 (10%)
Nervous system disorders	1 (10%)	0	0	1 (10%)
Headache	1 (10%)	0	0	1 (10%)
Paraesthesia	1 (10%)	0	0	1 (10%)
Skin and subcutaneous tissue disorders	1 (10%)	0	0	1 (10%)
Dry skin	1 (10%)	0	0	1 (10%)

TEAEs were coded using the Medical Dictionary for Regulatory Activities (MedDRA) version 21.0. All of the reported AEs were transient and had resolved by the end of the study. All adverse events that the investigator classified as related to the study drug were observed in the same participant. Abbreviations: TEAE, treatment-emergent adverse event.

4 Supplemental Material: Pharmacokinetics

4.1 SAD study: Pharmacokinetic parameters of zabedosertib (total) in plasma, all dose groups

Parameter	Unit	5 mg liquid N=6	15 mg liquid N=6	15 mg tablet N=5	30 mg tablet N=5	60 mg tablet N=6	120 mg tablet N=6	240 mg tablet N=5	480 mg tablet N=6	240 mg tablet fed state N=5	240 mg tablet, split dose fed state N=6	480 mg tablet split dose fed state N=6
**AUC**	mg·h/L	10.3 (35.9)	28.6 (31.4)	32.1 (30.6)	69.5 (28.5)	107 (60.0)	119 (41.7)	182 (50.3)	188 (34.1)	230 (32.6)	268 (23.7)	261 (15.5)
**AUC(0-24)**	mg·h/L	5.27 (18.2)	13.4 (17.6)	16.5 (24.0)	33.0 (12.8)	49.6 (35.2)	57.3 (22.8)	75.5 (25.7)	78.9 (18.5)	103 (19.7)	103 (15.6)	112 (7.97)
**CL/F**	L/h	0.487 (35.9)	0.525 (31.4)	0.467 (30.6)	0.432 (28.5)	0.561 (60.0)	1.01 (41.7)	1.32 (50.3)	2.56 (34.1)	1.04 (32.6)	0.897 (23.7)	1.84 (15.5)
**C_{max}**	mg/L	0.307 (13.0)	0.733 (16.4)	1.13 (25.8)	1.92 (9.64)	2.91 (31.6)	3.30 (21.9)	4.43 (16.6)	4.65 (18.3)	5.98 (18.4)	5.84 (15.4)	6.59 (10.0)
**MRT**	h	33.8 (23.6)	38.4 (19.0)	34.1 (15.1)	37.8 (19.0)	37.4 (33.8)	34.7 (28.7)	38.7 (27.0)	39.1 (24.2)	39.4 (24.3)	43.0 (18.7)	37.7 (13.7)
**$t_{1/2}$**	h	24.4 (23.2)	27.1 (20.0)	24.0 (16.5)	26.5 (24.9)	26.7 (35.3)	24.4 (29.0)	24.3 (23.0)	22.8 (27.0)	28.8 (27.0)	29.5 (25.2)	25.6 (13.3)
**t_{max} #**	h	2.50 [0.5-5.0]	5.00 [3.5-5.0]	2.00 [1.0-5.1]	3.50 [2.5-3.5]	3.00 [2.5-6.1]	3.25 [2.5-4.0]	2.50 [1.0-4.0]	1.50 [1.0-5.0]	4.00 [3.5-5.0]	15.8 [14.5-16.0]; 3.80 [2.5-4.0] $	16.0 [15.5-17.0]; 4.00 [3.5-5.0] $
**V_z/F**	L	17.1 (16.0)	20.5 (13.5)	16.2 (15.9)	16.5 (13.1)	21.6 (31.0)	35.6 (17.7)	46.2 (26.8)	84.2 (12.9)	43.4 (15.1)	38.2 (9.62)	72.0 (12.1)

Data are geometric means and coefficients of variation [%] unless indicated otherwise. # data are median [range]; $ t_{max} after 2nd dose (dose splitting). Dark-green shading highlights 2-period data. <u>Abbreviations:</u> AUC, area under the concentration–time curve from time zero to infinity after single-dose administration; AUC/D, AUC divided by dose; AUC(0-x), AUC from time zero to x hours post dose; AUC_{norm}, AUC divided by dose per kg body weight; CL/F, total body clearance of drug calculated after extravascular administration (apparent oral clearance); C_{max}, maximum observed drug concentration after single-dose administration; C_{max}/D, C_{max} divided by dose; MRT, mean residence time for extravascular administration; N, number of evaluable participants; $t_{1/2}$, half-life associated with the terminal slope; t_{max}, time to maximum concentration; V_z/F, apparent volume of distribution during terminal phase after extravascular administration.

4.2 SAD study: Assessment of dose proportionality of zabedosertib 15–480 mg taken as tablets under fasting conditions

Parameter (Unit)	Dose zabedosertib (mg)	N	Point estimate (geom. LS means)	Geometric 90% confidence interval	*P* #
AUC/D (h/L)	15	5	2.1400	1.5707, 2.9157	<0.0001
	30	5	2.3175	1.7010, 3.1575	
	60	6	1.7833	1.3446, 2.3650	
	120	6	0.9886	0.7454, 1.3111	
	240	5	0.7581	0.5564, 1.0329	
	480	6	0.3909	0.2947, 0.5184	
C_{max}/D (1/L)	15	5	0.0751	0.0637, 0.0887	<0.0001
	30	5	0.0640	0.0543, 0.0756	
	60	6	0.0485	0.0417, 0.0564	
	120	6	0.0275	0.0236, 0.0320	
	240	5	0.0185	0.0157, 0.0218	
	480	6	0.0097	0.0083, 0.0113	

Results of analysis of variance models. # p-value of F-statistics. Abbreviations: AUC/D, area under the concentration–time curve from time zero to infinity divided by dose; C_{max}/D, maximum observed drug concentration divided by dose; LS, least squares.

4.3 SAD study: Geometric mean unbound fraction (fu) of zabedosertib in plasma [%] after single oral administration of 5 mg to 480 mg zabedosertib and placebo under fasting conditions

Dose (mg)	N	Predose	3 h	4 h	5 h	24 h	72 h	96 h
5 (liquid)	6	2.48 (75.2)	2.32 (42.1)	-	-	2.74 (69.2)	-	2.12 (74.0)
15 (liquid)	6	2.58 (66.2)	3.01 (25.9)	-	-	2.52 (50.1)	-	2.33 (51.3)
30	6	2.44 (52.0)	3.66 (34.3)	-	-	3.75 (48.1)	-	3.00 (46.7)
60	6	2.97 (49.8)	4.57 (31.5)	-	-	3.34 (36.6)	-	1.83 (67.7)
120	6	1.75 (19.4)	3.51 (33.0)	-	-	2.68 (14.9)	-	1.91 (18.5)
240	5	1.52 (40.7)	3.43 (21.3)	4.52 (24.7)	6.33 (36.4)	3.27 (21.3)	-	1.96 (35.1)
480	6	1.44 (55.6)	5.55 (40.7)	-	-	3.09 (47.9)	-	2.02 (56.6)
Placebo	14	2.22 (50.7)	2.43 (61.5)	-	-	2.15 (44.6)	1.63 (14.3) #	1.97 (52.3) ##

Dose (mg)	N	Predose	4 h	5 h	15 h $	16 h $	17 h $	36 h $	84 h $
240 split	5	3.21 (39.0) €	6.90 (12.9) €	7.35 (21.7) €	7.53 (10.8)	7.48 (19.1)	7.69 (31.6)	3.90 (23.7)	2.81 (25.2)€
480 split	6	2.97 (41.6)	-	-	8.12 (25.1)	8.25 (13.4)	8.68 (17.9)	5.11 (22.2)	3.54 (23.9)
Placebo	4	2.23 (29.5)	-	-	4.00 (88.1)	-	-	3.48 (24.7)	2.41 (14.8)

Data are geometric means and coefficients of variation [%] in parentheses. # n = 2; ## n = 12; $ 3 h, 4 h, 5 h, 24 h and 72 h after the last dose (split dosing); € n = 4.

4.4 SAD study: Relationship between zabedosertib plasma concentration and *ex-vivo*-determined unbound fraction of zabedosertib – individual data of all zabedosertib- and placebo-treated study participants

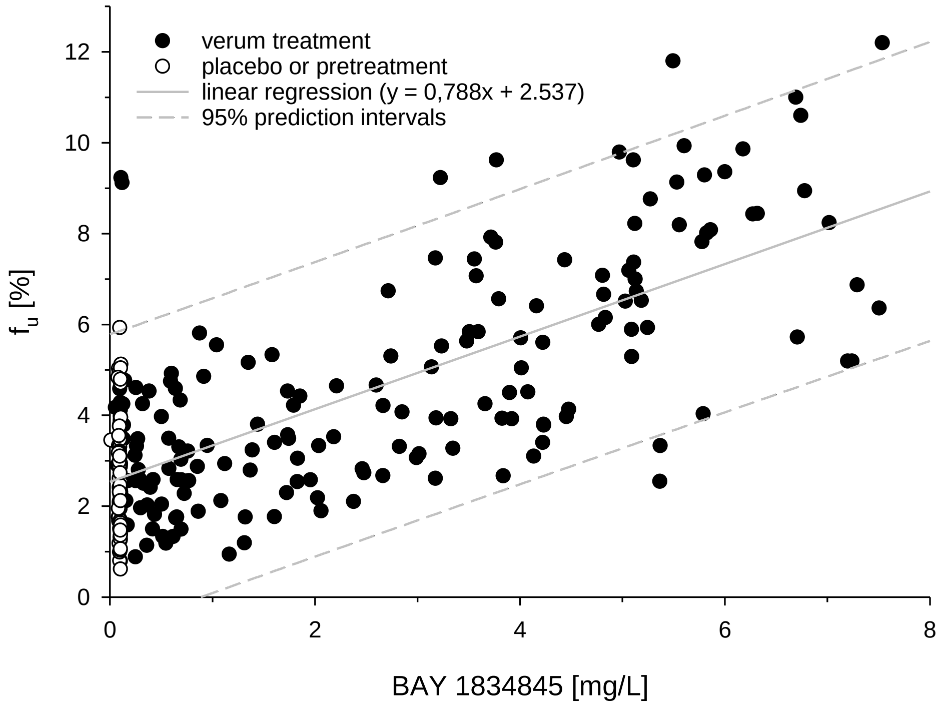

<u>Abbreviations:</u> BAY 1834845, zabedosertib; unbound fraction of zabedosertib.

4.5 MAD study, single-dose period: PK parameters of zabedosertib (unbound)

		QD dose groups		BID dose groups		
		**45 mg**	**120 mg**	**60 mg**	**120 mg**	**200 mg**
**Parameter**	**Unit**	N=7 #	N=8	N=8	N=8	N=8
C_{max}	mg/L	0.0899 (12.8)	0.184 (25.2)	0.125 (14.1)	0.216 (19.9)	0.251 (22.8)
C_{max}/D	1/L	0.00200 (12.8)	0.00153 (25.2)	0.00208 (14.1)	0.00180 (19.9)	0.00126 (22.8)
t_{max} ##	H	3.0 [1.0 - 5.0]	4.5 [3.0 - 6.0]	5.0 [2.0 - 5.0]	3.0 [1.0 - 6.0]	4.4 [4.0 – 7.7]
AUC	mg·h/L	2.18 (30.0)	5.04 (45.7)	4.26 (27.8)	6.14 (19.0)	6.74 (27.0)
AUC/D	h/L	0.0484 (30.0)	0.0420 (45.7)	0.0711 (27.8)	0.0512 (19.0)	0.0337 (27.0)
AUC(0-12)	mg·h/L	0.790 (11.6)	1.60 (21.7)	1.13 (12.5)	1.99 (18.7)	2.09 (22.2)
AUC(0-12)/D	h/L	0.0176 (11.6)	0.0134 (21.7)	0.0188 (12.5)	0.0166 (18.7)	0.0104 (22.2)
AUC(0-24)	mg·h/L	1.31 (16.8)	2.77 (27.4)	2.00 (7.90)	3.36 (15.7)	3.55 (21.8)
AUC(0-24)/D	h/L	0.0290 (16.8)	0.0231 (27.4)	0.0333 (7.90)	0.0280 (15.7)	0.0177 (21.8)
$t_{1/2}$	H	19.2 (30.5)	22.8 (28.4)	28.8 (26.0)	25.0 (14.1)	24.4 (25.7)
CL/F	L/h	20.6 (30.0)	23.8 (45.7)	14.1 (27.8)	19.5 (19.0)	29.7 (27.0)
V_z/F	L	571 (12.6)	782 (20.6)	584 (10.6)	704 (17.4)	1040 (17.4)

Data are geometric means and coefficients of variation [%] unless otherwise indicated. The concentration of unbound zabedosertib was calculated on the basis of the total concentration of zabedosertib and the fraction of unbound zabedosertib.

1 participant discontinued the study prematurely before single-dose administration in period 2.

median [range]. Abbreviations: AUC, area under the concentration–time curve from time zero to infinity after single-dose administration; AUC/D, AUC divided by dose; AUC(0-x), AUC from time zero to x hours post dose; BID, twice daily; CL/F, total body clearance of drug calculated after extravascular administration (apparent oral clearance); C_{max}, maximum observed drug concentration after single-dose administration; C_{max}/D, C_{max} divided by dose; N, number of evaluable participants; QD, once daily; $t_{1/2}$, half-life associated with the terminal slope; t_{max}, time to maximum concentration; Vz/F, apparent volume of distribution during terminal phase after extravascular administration.

4.6 MAD study, multiple-dose period: Geometric mean zabedosertib plasma concentration–time curves (±SD) obtained during repeated administration of zabedosertib tablets under fed conditions

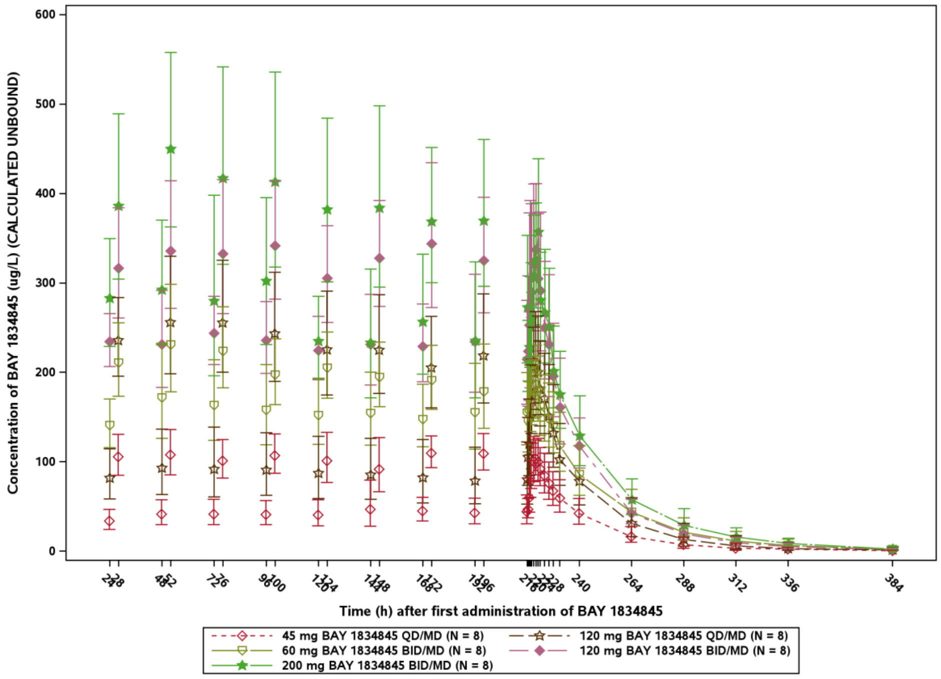

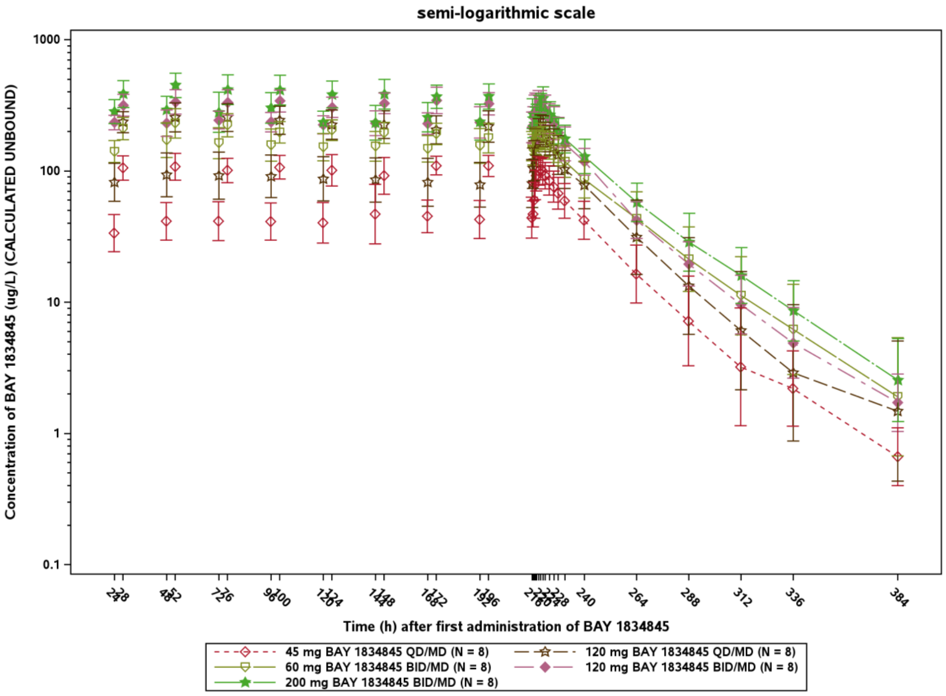

Data are geometric means and standard deviations. Top: linear scale; bottom: semi-logarithmic scale. On days 1 to 9, samples were taken predose (C_{trough}) and 4 h postdose (~C_{max}). Abbreviations: BAY 1834845, zabedosertib; BID, twice daily; QD, once daily; MD, multiple-dose administration. For abbreviations of pharmacokinetic parameters, see the list of abbreviations in Section 6 of the manuscript.

4.7 MAD study, multiple-dose period: PK parameters of zabedosertib (total) obtained after repeated oral administration of zabedosertib to healthy young men

		QD treatment		BID treatment		
		45 mg	120 mg	60 mg	120 mg	200 mg
**Parameter**	**Unit**	N=8	N=8	N=8	N=8	N=8
$AUC(0\text{-}12)_{md}$	mg·h/L	27.7 (30.4)	42.4 (20.2)	46.5 (23.7)	62.2 (19.4)	63.5 (15.8)
$AUC(0\text{-}24)_{md}$	mg·h/L	47.4 (34.2)	72.0 (24.4)	93.0 (23.7) €	124 (19.4) €	127 (15.8) €[e]
$AUC(0\text{-}24)_{md}/D$	h/L	1.05 (34.2)	0.600 (24.4)	0.775 (23.7) €	0.518 (19.4) €	0.317 (15.8) €
$C_{max,md}$	mg/L	2.81 (29.3)	4.18 (16.5)	4.29 (22.0)	6.01 (16.8)	6.19 (12.3)
$t_{max,md}$ #	h	3.5 [1.5 - 5.0]	4.0 [2.0 - 5.0]	4.0 [1.5 - 5.0]	3.9 [1.0 – 5.8]	4.5 (0 - 5.0)
$t_{1/2,md}$	h	21.8 (28.0)	23.1 (29.5)	29.5 (25.2)	26.5 (19.4)	28.4 (19.5)
$C_{av,md}$	mg/L	1.98 (34.2)	3.00 (24.4)	3.87 (23.7)	5.18 (19.4)	5.29 (15.8)
CL_{md}/F	L/h	0.949 (34.2)	1.67 (24.4)	1.29 (23.7)	1.93 (19.4)	3.15 (15.8)
$V_{z,md}/F$	L	29.8 (11.1)	55.6 (9.43)	54.9 (10.2)	73.4 (9.31)	129 (12.0)
PTF	%	72.8 (19.3)	66.2 (31.7)	18.8 (30.6)	30.3 (26.9)	30.3 (36.2)
R_AAUC §		1.26 (19.0)	1.04 (12.5)	1.42 (16.1)	1.28 (11.7)	1.31 (11.4)
R_AC_{max} §		1.19 (16.5)	1.05 (13.1)	1.27 (17)	1.24 (12.7)	1.19 (12.3)
R_{LIN} §		0.700 (24.7)	0.499 (30.3)	0.322 (22.8)	0.330 (19.8)	0.314 (20.0)

Data are geometric means and coefficients of variation [%] unless indicated otherwise. # Median (minimum – maximum). € 2*AUC(0-12); § Day 10 in Period 1/Period 2 (all groups except the 200 mg BID group); Day 10 in Period 2/Period 1 (200 mg BID group). Abbreviations:.

$AUC(0\text{-}x)_{md}$, area under the concentration–time curve from time zero to x hours post dose after repeated administration; $AUC(0\text{-}x)_{md}/D$, $AUC(0\text{-}x)_{md}$ divided by dose; BID, twice daily; $C_{av,md}$, average concentration within a dosing interval after multiple dosing; CL_{md}/F, total body clearance of drug calculated after extravascular application; $C_{max,md}$, maximum drug concentration in plasma after repeated administration; md, multiple-dose administration; D, daily dose (e.g., 120 mg for 60 mg BID); PTF, peak-trough fluctuation; QD, once daily; R_AAUC, accumulation ratio calculated from $AUC\tau$; R_AC_{max}, accumulation ratio calculated from; τ, dosing interval; R_{LIN}, linearity factor of pharmacokinetics after repeated administration of identical doses; $t_{1/2,md}$, half-life associated with terminal slope; $t_{max,md}$, time to reach maximum drug concentration; $V_{z,md}/F$, apparent volume of distribution during terminal phase after extravascular administration.

4.8 FE/abs.BA study: Mean [$^{13}C_6$]-zabedosertib plasma concentration–time curves obtained during and after a 15-minute intravenous infusion of 0.1 mg [$^{13}C_6$]-zabedosertib

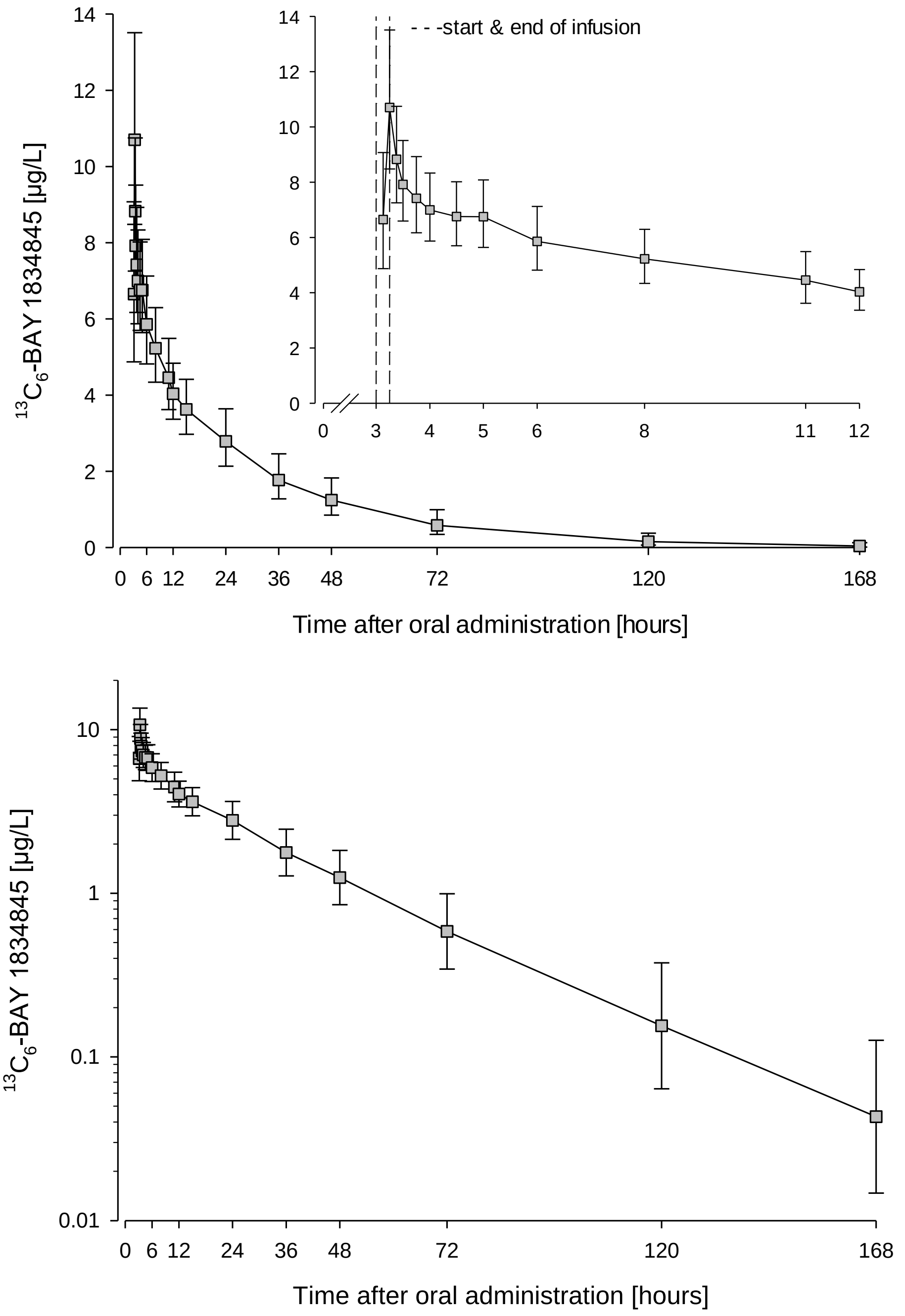

Data are geometric means and standard deviations. Top: linear scale; bottom: semi-logarithmic scale. N = 10 participants. Limit of quantification: 0.01 µg/L. The infusion started 3 hours after oral administration of a 120 mg zabedosertib tablet in fasted state. Abbreviations: BAY 1834845, zabedosertib.

4.9 SAD study: Assessment of the impact of drug formulation, concomitant food intake and dose splitting

A

Dose	Parameter	Treatment ratio	Point estimate geom. LS means	90% confidence interval	Geom. CV%
15 mg	AUC	solid/liquid #	1.2448	1.0195, 1.5199	14.9
	C_{max}	solid/liquid #	1.6156	1.2445, 2.0972	19.5

B

Dose	Parameter	Treatment ratio	Point estimate geom. LS means	90% confidence interval
240 mg (tablet)	AUC	Split fed / fasted	1.4709	1.0121, 2.1375
		Fed / fasted	1.2637	0.8553, 1.8672
		Split fed / fed #	1.1639	0.8009, 1.6914
	C_{max}	Split fed / fasted	1.3184	1.1033, 1.5754
		Fed / fasted	1.3484	1.1195, 1.6240
		Split fed / fed #	0.9778	0.8183, 1.1684

C

Dose	Parameter	Treatment ratio	Point estimate geom. LS mean	90% confidence interval	Geom. CV%
480 mg (tablet)	AUC	Split fed / fasted	1.3918	1.0620, 1.8238	26.3
	C_{max}	Split fed / fasted	1.4182	1.2171, 1.6525	14.7

intraindividual comparison. Abbreviations: AUC, area under the concentration–time curve from time zero to infinity after single-dose administration; C_{max}, maximum observed drug concentration after single-dose administration.
